# Global, regional, and national burden of depressive disorders among women of childbearing age, 1990–2021: a joinpoint regression analysis for the Global Burden of Disease Study 2021

**DOI:** 10.3389/fpubh.2025.1566240

**Published:** 2025-05-15

**Authors:** Lulu Yang, Yuting Luo, Qiuhan Bi, Binbin Luo

**Affiliations:** Department of Science and Education, The Second People's Hospital of Hefei, Hefei Hospital Affiliated to Anhui Medical University, Hefei, Anhui, China

**Keywords:** depressive disorders, women of childbearing age, Global Burden of Disease Study, joinpoint regression analysis, epidemiology

## Abstract

**Background:**

Depressive disorders, as a mental disorder, are prevalent among women, particularly women of childbearing age (WCBA). However, comprehensive epidemiological studies on this population appear to be limited globally. To investigate the longitudinal epidemiology of depressive disorders among women aged 15–49 years worldwide.

**Method:**

Estimates of annual incidence, prevalence, and disability-adjusted life years (DALYs) of depressive disorders for seven age groups (15–49 years) of women were extracted from the 2021 Global Burden of Disease Study, including 95% uncertainty intervals (UI). Age-standardized incidence rates (ASIR), age-standardized prevalence rates (ASPR), and age-standardized DALYs rates (ASDR) of depressive disorders in this population were estimated using direct age-standardization. Joinpoint regression analysis was employed to assess temporal trends in depressive disorders among this cohort from 1990 to 2021.

**Result:**

From 1990 to 2021, ASIR, ASPR, and ASDR of depressive disorders among WCBA increased globally. Regionally, incidence, prevalence, and DALYs rates rose in high-income areas such as North America, while a decline was observed in East Asia. Among 204 countries, Greenland, the United States of America, and Greece reported the highest ASIR, ASPR, and ASDR in 2021, with Mexico exhibiting the fastest increase. Additionally, from 2019 to 2021, most countries and regions worldwide experienced a continuous rise in the burden of depressive disorders among WCBA.

**Conclusion:**

Our study provides accurate age-standardized estimates, facilitating effective comparisons across different regions. We found a sharp increase in the global burden of depressive disorders among WCBA in almost all countries and regions after 2019, highlighting an urgent need for governments to develop flexible health policies to mitigate the escalating threat of depressive disorders in this demographic.

## Highlights

- This study provides age-standardized estimates of the epidemiological indicators of the burden, facilitating effective comparisons across different regions.
- The burden of depressive disorders among WCBA, including age-standardized prevalence, incidence, and DALYs rate, is rising in most areas, especially in high-income regions after 2019.
- Our findings would highlight an urgent need for governments to develop flexible health policies to mitigate the escalating threat of depressive disorders in this demographic.

## Introduction

Depressive disorders are a common mental disorder and a leading cause of disability worldwide, accounting for 37.3% of disability-adjusted life years (DALYs) due to mental disorders in 2019 (1). Women are nearly twice as likely as men to experience depressive disorders, and this disparity persists during the childbearing years (2, 3). Pregnancy, in the context of depressive disorders, is a challenging event and may be associated with adverse pregnancy outcomes such as preterm birth, low birth weight, and maternal suicide (4). Therefore, it is crucial to elucidate the epidemiology of depressive disorders among women of childbearing age (WCBA) comprehensively. However, the epidemiology of depressive disorders in this demographic has been investigated in very limited geographic regions, mostly within specific countries using national health system data, lacking age-standardized processes to track and compare the longitudinal burden of depressive disorders among WCBA globally (5, 6). This limitation hinders further cross-regional and cross-national comparisons. Undoubtedly, providing global estimates of age-standardized rates (ASR) of depressive disorders among WCBA over the past decades could fill gaps in disease statistics, enhancing the understanding of the evolution of depressive disorders in this demographic both globally and in specific geographical locations. This information can inform policy decisions and resource prioritization for this critical yet inadequately addressed mental health condition. This limitation hinders further cross-regional and cross-national comparisons. Undoubtedly, providing global estimates of age-standardized rates (ASR) of depressive disorders among WCBA over the past decades could fill gaps in disease statistics, enhancing the understanding of the evolution of depressive disorders in this demographic both globally and in specific geographical locations. This information can inform policy decisions and resource prioritization for this critical yet inadequately addressed mental health condition. Based on evidence of rising mental health burdens globally (7, 8), geographic disparities in healthcare access (9), and emerging reports on the mental health impacts of the COVID-19 pandemic (10), we hypothesized that the global ASR of depressive disorders among WCBA would increase from 1990 to 2021, with accelerated growth after 2019 due to pandemic-related stressors (11, 12). Heterogeneous trends in disease burden would emerge across countries and regions, may reflect differences in healthcare system resilience and prioritization of mental health services (13).

In this study, we extracted data from the Global Burden of Disease (GBD) Study 2021 and calculated age-standardized incidence rates (ASIR), age-standardized prevalence rates (ASPR), and age-standardized DALYs rates (ASDR) of depressive disorders among WCBA from 1990 to 2021, further analyzing their temporal trends at global, regional, and national levels over the past 30 years.

## Method

### Study population

WCBA exhibit strong reproductive capability and experience cyclical hormonal changes. The World Health Organization defines WCBA as those between 15 and 49 years old (14).

### Data sources

The methodological details of the GBD Study 2021 have been previously published. These studies comprehensively detail health burdens associated with 371 diseases and injuries and 88 risk factors across 204 countries and territories (15, 16). We retrieved estimates of incidence, prevalence, and DALYs for depressive disorders, along with their 95% uncertainty intervals (UI), for seven age groups (15–19, 20–24, 25–29, 30–34, 35–39, 40–44, 45–49 years) from 1990 to 2021 using the Global Health Data Exchange (GHDx) query tool.^1^ These uncertainty intervals are based on the 25th and 975th ranked values of 1,000 independent estimates according to GBD algorithms. Depressive disorders are classified using the ICD-10 and include two major types: major depressive disorder (MDD) and dysthymia. MDD is defined as a mood disorder characterized by one or more major depressive episodes, while dysthymia is a chronic form of depressive disorder with less severe symptoms but greater persistence than MDD (17).

### Joinpoint regression analysis

To estimate ASIR, ASPR, and ASDR of depressive disorders among WCBA, direct age standardization was employed. This method assumes that the distribution of incidence rates is a weighted sum of independent Poisson random variables, and all rates are expressed per 100,000 population (18). Joinpoint regression analysis was used to assess trends in ASIR, ASPR, and ASDR. This analysis method identifies points where significant changes in trends occur, segmenting the overall trend accordingly. Each segment’s epidemiological trend is evaluated by calculating the relative annual percentage change (APC) and 95% confidence interval (CI) (19). The average annual percentage change (AAPC) was also calculated for an overall assessment, encompassing the entire study period from 1990 to 2021. An increasing trend is inferred if the lower limit of the 95% CI of the APC or AAPC estimates exceeds zero. Conversely, a decreasing trend is indicated if the upper limit of the 95% CI is below zero. Otherwise, the trend is considered stable over time (19, 20).

### Statistical analysis

The environment for joinpoint regression analysis was configured using the “configr” package in R version 4.3.0. The “ggplot2” package was used for data visualization, and the “epitools” package was employed for calculating age-standardized rates. All analyses were conducted using R software (v.4.3.0).^2^

### Ethical approval

Ethical approval and consent to participate were not necessary for this study.

## Results

### Depressive disorders among WCBA: global level

**Figure 1:**
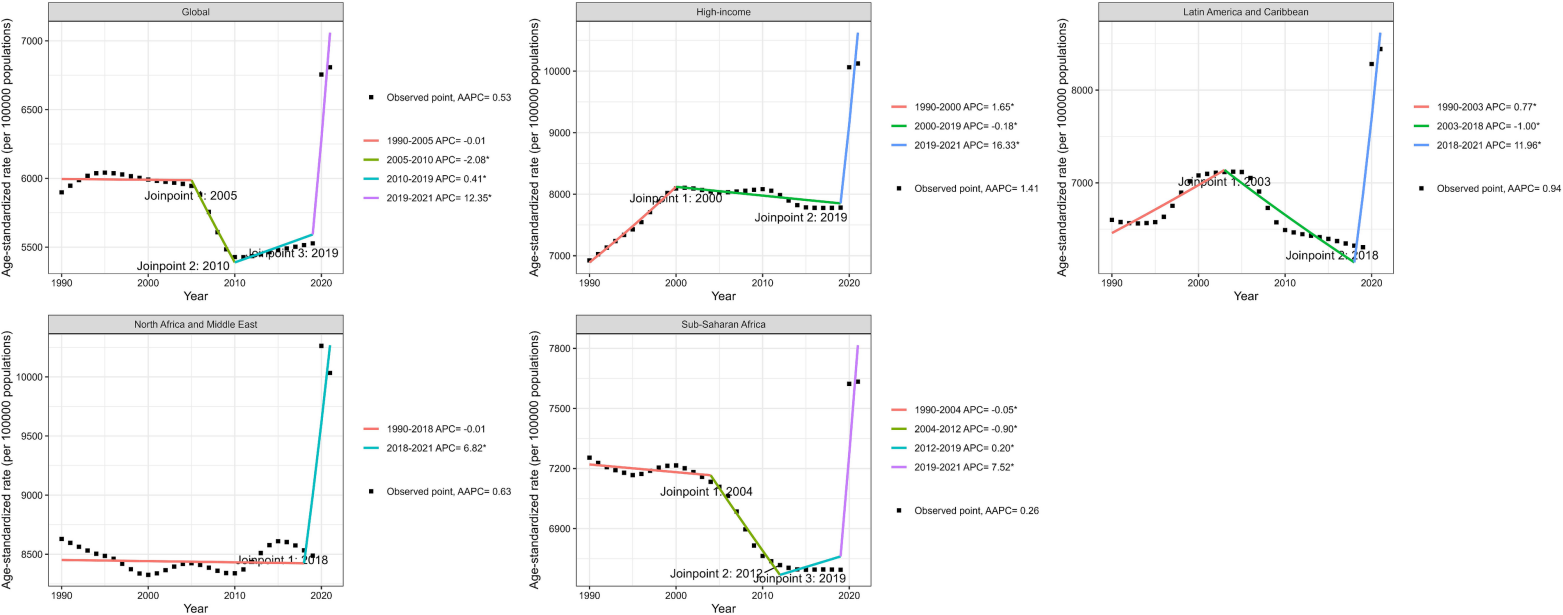
Joinpoint regression analysis of age-standardized incidence rate of depression among women of childbearing age at the global and four regions (High income, Latin America and Caribbean, North Africa and Middle East, Sub-Saharan Africa) from 1990 to 2021. *p*-value *p < 0.05.

In 1990, the global ASIR of depressive disorders among WCBA was 5898.5 cases per 100,000 population (95% UI: 4486.52 to 7740.66). By 2021, the ASIR had risen to 6808.01 cases per 100,000 population (95% UI: 5049.99 to 9106.66), indicating an increase of 909.51 cases per 100,000 over the period (AAPC = 0.53, 95% CI: 0.39 to 0.67%; *p* < 0.001) (Table 1; Figure 1). Additionally, the ASPR was 5545.28 cases per 100,000 population (95% UI: 4447.47 to 6858.89) in 1990 and increased to 6173.45 cases per 100,000 population (95% UI: 4883.27 to 7781.73) in 2021, reflecting an increase of 628.17 cases per 100,000 (AAPC = 0.39, 95% CI: 0.27 to 0.5%; *p* < 0.001) (Table 1; Figure 1). Moreover, the ASDR was 948.86 per 100,000 population (95% UI: 617.06 to 1369.93) in 1990, which rose to 1073.5 per 100,000 population (95% UI: 686.73 to 1562.48) in 2021, representing an increase of 124.64 per 100,000 (AAPC = 0.45, 95%CI: 0.33 to 0.58%; *p* < 0.001) (Table 1; Figure 1).

**Table 1:** ASIR, ASPR and ASDR of depression among women of childbearing age in 1990 and 2021, and change from 1990 to 2021 at the global and regional level.

Location	ASIR, per 100,000 (95% UI)		AAPC (95% CI)	ASPR, per 100,000 (95% UI)		AAPC (95% CI)	ASDR, per 100,000 (95% UI)		AAPC (95% CI)
	1990	2021		1990	2021		1990	2021	
Global	5898.5 (4486.52 to 7740.66)	6808.01 (5049.99 to 9106.66)	0.53 (0.39 to 0.67)	5545.28 (4447.47 to 6858.89)	6173.45 (4883.27 to 7781.73)	0.39 (0.27 to 0.5)	948.86 (617.06 to 1369.93)	1073.5 (686.73 to 1562.48)	0.45 (0.33 to 0.58)
Central Europe, eastern Europe, and central Asia	4909.81 (3613.25 to 6576.65)	6081.61 (4367.75 to 8244.82)	0.73 (0.65 to 0.82)	4755.93 (3778.43 to 5963.83)	5526.69 (4297.43 to 7026.99)	0.51 (0.45 to 0.57)	801.32 (512.48 to 1163.65)	962.53 (610.95 to 1414.42)	0.62 (0.55 to 0.7)
Central Asia	4676.28 (3367.02 to 6381.95)	5623.36 (3891.23 to 7918.02)	0.7 (0.58 to 0.82)	4607.34 (3546.52 to 5930.09)	5231.16 (3920.92 to 6874.97)	0.48 (0.4 to 0.56)	772.01 (488.55 to 1132.2)	903.45 (561.45 to 1363.66)	0.6 (0.51 to 0.69)
Central Europe	4115.36 (3043.59 to 5505.79)	4795.85 (3393.82 to 6608.56)	0.56 (0.45 to 0.67)	4241.09 (3332.5 to 5343.85)	4677.25 (3608.8 to 6022.43)	0.36 (0.29 to 0.43)	695.87 (442.07 to 1011.41)	788.38 (492.87 to 1163.38)	0.46 (0.37 to 0.55)
Eastern Europe	5392.25 (3903.27 to 7297.88)	6990.81 (4956.63 to 9586.01)	0.85 (0.68 to 1.02)	5063.54 (3985.84 to 6407.4)	6116.21 (4742.5 to 7829.81)	0.62 (0.49 to 0.74)	864.27 (549.26 to 1266.81)	1083.11 (684.77 to 1608.23)	0.74 (0.58 to 0.9)
High income	6925.13 (5475.65 to 8753.68)	10124.57 (7726.81 to 13220.18)	1.41 (1.24 to 1.57)	6244.01 (5129.85 to 7559.57)	8315.11 (6625.31 to 10424.26)	1.05 (1.02 to 1.08)	1104.19 (735.81 to 1572.03)	1538.48 (1014.95 to 2231.33)	1.22 (1.13 to 1.3)
Australasia	8977.64 (6898.68 to 11536.06)	10269.64 (7057.12 to 14369.31)	0.5 (0.44 to 0.56)	7599.59 (6055.54 to 9386.46)	8462.14 (6257.29 to 11300.83)	0.4 (0.36 to 0.45)	1380.17 (896.99 to 1991.52)	1563.09 (962.3 to 2373.22)	0.46 (0.41 to 0.51)
High-income Asia Pacific	3838.31 (2978.91 to 4945.44)	5010.7 (3763.15 to 6511.04)	0.93 (0.79 to 1.07)	3508.02 (2868.29 to 4262.88)	4293.05 (3428.65 to 5331.89)	0.7 (0.57 to 0.83)	618.37 (403.62 to 885.93)	783.79 (508.07 to 1134.18)	0.82 (0.7 to 0.94)
High-income North America	7145.82 (5538.1 to 9178.7)	12689.95 (9828.58 to 16139.08)	1.99 (1.56 to 2.42)	7007.87 (5700.86 to 8531.26)	10443.59 (8434.35 to 12835.72)	1.4 (1.27 to 1.54)	1193.29 (788.61 to 1716.28)	1929.21 (1284.02 to 2772.95)	1.65 (1.37 to 1.92)
Southern Latin America	6983.96 (5277.99 to 9228.68)	8133.97 (5867.86 to 11029.02)	0.63 (0.21 to 1.05)	5733.18 (4480.59 to 7315.73)	6509.67 (4896.55 to 8513.81)	0.57 (0.45 to 0.69)	1063.1 (688.88 to 1563.55)	1221.46 (760.65 to 1814.09)	0.56 (0.18 to 0.95)
Western Europe	8121.24 (6445.97 to 10229.83)	10094.71 (7338.27 to 13758.87)	0.9 (0.71 to 1.09)	6951.88 (5732.39 to 8402.04)	8254.89 (6326.11 to 10733.02)	0.72 (0.56 to 0.88)	1258.72 (836.26 to 1781.26)	1527.48 (969.93 to 2272.7)	0.71 (0.45 to 0.97)
Latin America and Caribbean	6600.56 (4912.9 to 8789.19)	8443.17 (6199.99 to 11,353)	0.94 (0.51 to 1.37)	5511.68 (4306.39 to 6977.88)	6724.81 (5176.69 to 8655.52)	0.75 (0.4 to 1.09)	996.28 (634.13 to 1463.93)	1245.14 (779.25 to 1833.11)	0.84 (0.46 to 1.22)
Andean Latin America	4,737 (3296.37 to 6625.44)	6320.57 (4292.05 to 9087.82)	1.1 (0.52 to 1.67)	4327.74 (3255.28 to 5700.01)	5361.92 (3933.81 to 7199.44)	0.8 (0.39 to 1.23)	751.79 (460.41 to 1140.63)	966.63 (579.65 to 1490.12)	0.95 (0.45 to 1.45)
Caribbean	7697.77 (5552.01 to 10488.7)	8029.7 (5444.45 to 11483.95)	0.21 (0.13 to 0.29)	6320.15 (4798.37 to 8195.97)	6502.68 (4706.8 to 8863.19)	0.15 (0.1 to 0.2)	1161.78 (729.68 to 1756.61)	1195.95 (719.21 to 1842.41)	0.16 (0.1 to 0.22)
Central Latin America	5119.69 (3689.12 to 6999.46)	7882.55 (5720.17 to 10666.28)	1.69 (1.38 to 2.01)	4465.04 (3447.45 to 5739.42)	6293.78 (4790.95 to 8147.72)	1.34 (1.09 to 1.6)	791.2 (499.51 to 1175.67)	1168.22 (724.13 to 1736.5)	1.52 (1.25 to 1.78)
Tropical Latin America	8304.3 (6295.63 to 10868.09)	9756.89 (7276.88 to 12821.9)	0.74 (0.3 to 1.19)	6672.9 (5284.8 to 8352.26)	7633.97 (5948.15 to 9648.14)	0.61 (0.25 to 0.98)	1225.46 (794.24 to 1783.06)	1419.51 (900.1 to 2073.83)	0.66 (0.26 to 1.06)
North Africa and Middle East	8629.46 (6216.24 to 11853.62)	10032.79 (6876.71 to 14305.05)	0.63 (0.33 to 0.93)	7378.92 (5652.37 to 9576.45)	8318.57 (6135.59 to 11121.61)	0.49 (0.39 to 0.58)	1323.9 (828.06 to 1967.04)	1515.15 (916.91 to 2301.13)	0.57 (0.3 to 0.83)
South Asia	7116.76 (5321.38 to 9440.27)	7283.76 (5421.29 to 9654.43)	0.19 (0.08 to 0.31)	6252.32 (4,948 to 7775.32)	6390.39 (5049.98 to 8025.78)	0.16 (0.06 to 0.25)	1088.15 (696.41 to 1593.93)	1118.19 (713.21 to 1625.21)	0.19 (0.09 to 0.3)
Southeast Asia, East Asia, and Oceania	4092.56 (3124.22 to 5324.9)	3319.25 (2454.66 to 4388.12)	−0.63 (−0.9 to − 0.36)	4476.61 (3619.35 to 5478.52)	3951.58 (3163.68 to 4890.79)	−0.39 (−0.54 to − 0.24)	721.1 (471.43 to 1041.13)	612.17 (397.14 to 880.17)	−0.5 (−0.7 to − 0.31)
East Asia	4412.52 (3367.36 to 5732.14)	2956.12 (2227.57 to 3840.52)	−1.15 (−1.52 to − 0.78)	4689.83 (3804.73 to 5717.47)	3,700 (2994.89 to 4515.91)	−0.77 (−0.91 to − 0.64)	766.37 (500.81 to 1106.86)	560.51 (365.8 to 799.17)	−0.96 (−1.25 to − 0.66)
Oceania	3968.59 (2766.94 to 5579.09)	4125.32 (2738.85 to 6006.85)	0.1 (0.01 to 0.19)	4395.87 (3303.6 to 5788.5)	4495.59 (3337.06 to 5970.94)	0.06 (0 to 0.11)	698.73 (437.04 to 1054.93)	720.59 (430.31 to 1107.37)	0.08 (0.01 to 0.15)
Southeast Asia	3196.08 (2353.81 to 4271.57)	3829.96 (2729.29 to 5227.5)	0.59 (0.54 to 0.64)	3875.31 (3052.48 to 4886.27)	4294.28 (3358.87 to 5457.74)	0.33 (0.3 to 0.36)	593.89 (383.57 to 865.93)	683.77 (434.95 to 1007.98)	0.45 (0.41 to 0.49)
Sub-Saharan Africa	7254.2 (5252.42 to 9910.81)	7633.94 (5462.37 to 10414.09)	0.26 (0.18 to 0.33)	6831.1 (5330.11 to 8666.86)	7064.42 (5454.68 to 8999.95)	0.17 (0.13 to 0.22)	1154.41 (731.81 to 1686.19)	1209.2 (760.47 to 1776.78)	0.2 (0.03 to 0.38)
Central sub-Saharan Africa	10640.71 (7408.07 to 15062.94)	11270.02 (7523.17 to 16274.56)	0.27 (0.06 to 0.49)	8977.33 (6650.3 to 11919.06)	9386.69 (6800.91 to 12768.75)	0.21 (0.04 to 0.37)	1597.25 (989.08 to 2430.71)	1695.2 (994.94 to 2610.73)	0.28 (0.09 to 0.47)
Eastern sub-Saharan Africa	7375.7 (5319.95 to 10067.85)	7985.77 (5630.58 to 11024.96)	0.3 (0.23 to 0.37)	7087.75 (5520.1 to 9011.64)	7444.18 (5696.51 to 9554.62)	0.18 (0.13 to 0.23)	1188.39 (751.52 to 1736.98)	1273.15 (789.45 to 1889.91)	0.26 (0.2 to 0.32)
Southern sub-Saharan Africa	6761.59 (5111.67 to 8866.45)	8722.61 (6487.11 to 11549.81)	1.01 (0.71 to 1.32)	6388.08 (5117.99 to 7859.14)	7672.13 (6067.56 to 9620.79)	0.73 (0.51 to 0.94)	1080.34 (703.43 to 1562.11)	1329.3 (856.5 to 1943.43)	0.81 (0.58 to 1.04)
Western sub-Saharan Africa	6321.17 (4550.59 to 8648.31)	6114.54 (4369.47 to 8446.05)	−0.01 (−0.3 to 0.28)	6101.55 (4749.55 to 7767.06)	5969.48 (4614.72 to 7618.2)	0 (−0.2 to 0.19)	1017.39 (639.5 to 1494.49)	996.09 (626.15 to 1467.43)	0.02 (−0.23 to 0.27)

ASIR, age-standardized incidence rate; ASPR, age-standardized prevalence rate; ASDR, age-standardized DALYs rate; SDI, sociodemographic index; UI, uncertainty interval; AAPC, average annual percent change; CI, confidence interval.

### Depressive disorders among WCBA: regional level

In 2021, regional studies revealed that among the seven global super-regions, the highest ASIR of depressive disorders among WCBA was observed in high-income regions, with 10,124.57 cases per 100,000 population (95% UI: 7726.81 to 13220.18), followed by North Africa and the Middle East, also with 10,032.79 cases per 100,000 population (95% UI: 6876.71 to 14305.05) (Table 1).

In contrast, the lowest ASIR were observed in Southeast Asia, East Asia, and Oceania, with 3,319.25 cases per 100,000 population (95% UI: 2454.66 to 4388.12). Geographically, the highest ASIR was in High-income North America, with 12,689.95 cases per 100,000 population (95%UI: 9828.58 to 16139.08), followed by Central Sub-Saharan Africa with 11,270.02 cases per 100,000 population (95%UI: 7523.17 to 16274.56) (Table 1). In contrast, Southeast Asia, East Asia, and Oceania exhibited significantly lower ASIR, including East Asia with 2,956.12 cases per 100,000 population (95% UI: 2227.57 to 3840.52), Oceania with 4,125.32 cases per 100,000 population (95% UI: 2738.85 to 6006.85), and Southeast Asia with 3,829.96 cases per 100,000 population (95% UI: 2729.29 to 5227.5) (Table 1).

From 1990 to 2021, the changes in ASIR varied across different regions. An increasing trend was observed in Central Europe, Eastern Europe, and Central Asia (AAPC = 0.73, 95% CI: 0.65 to 0.82%; *p* < 0.001), High-income regions (AAPC = 1.41, 95%CI: 1.24 to 1.57%; *p* < 0.001), Latin America and the Caribbean (AAPC = 0.94, 95%CI: 0.51 to 1.37%; *p* < 0.001), North Africa and the Middle East (AAPC = 0.63, 95% CI: 0.51 to 1.37%; *p* < 0.001), South Asia (AAPC = 0.19, 95% CI: 0.08 to 0.31%; *p* < 0.001), and Sub-Saharan Africa (AAPC = 0.26, 95% CI: 0.18 to 1.33%; *p* < 0.001) (Table 1). In contrast, a decreasing trend was observed in Southeast Asia, East Asia, and Oceania (AAPC = −0.63, 95% CI: −0.9% to −0.36%; *p* < 0.001) (Table 1). Among the 21 regions, the most significant increasing trend in ASIR was in High-income North America (AAPC = 1.99, 95% CI: 1.56–2.42%; *p* < 0.001), followed by Central Latin America (AAPC = 1.69, 95% CI: 1.38 to 2.01%; *p* < 0.001), and Andean Latin America (AAPC = 1.1, 95% CI: 0.52 to 1.67%; *p* < 0.001) (Table 1). Only East Asia exhibited a decreasing trend (AAPC = −1.15, 95% CI: −1.52% to −0.78%; *p* < 0.001) (Table 1).

The ASPR of depressive disorders among WCBA was highest in North Africa and the Middle East at 8,318.57 per 100,000 population (95% UI: 6135.59 to 11121.61), followed by High-income regions at 8,315.11 per 100,000 population (95% UI: 6625.31 to 10424.26). The lowest ASPR was observed in Southeast Asia, East Asia, and Oceania, at 3,951.58 per 100,000 population (95% UI: 3163.68 to 4890.79) (Table 1). Over the past 30 years, ASPR has consistently increased in all global super-regions except Southeast Asia, East Asia, and Oceania. The most significant increase was seen in High-income regions (AAPC = 1.05, 95% CI: 1.02 to 1.08%; *p* < 0.001) (Table 1). Geographically, in 2021, the highest ASPR was in High-income North America, with 10,443.59 cases per 100,000 population (95% UI: 8434.35 to 12835.72), followed by Central Sub-Saharan Africa with 9,386.69 cases per 100,000 population (95% UI: 6800.91 to 12768.75). East Asia exhibited the most significant decreasing trend in ASPR from 1990 to 2021 (AAPC = −0.77, 95% CI: −0.91% to −0.64%; *p* < 0.001) (Table 1).

Over the past 30 years, ASDR for depressive disorders among WCBA also showed an increasing trend in all global super-regions except Southeast Asia, East Asia, and Oceania. In 2021, the highest ASDR was observed in High-income regions, with 1,538.48 per 100,000 population (95% UI: 1014.95 to 2231.33), and the lowest in Southeast Asia, East Asia, and Oceania, with 612.17 per 100,000 population (95% UI: 397.14 to 880.17) (Table 1). The most significant increase over 30 years was in High-income North America (AAPC = 1.65, 95% CI: 1.37 to 1.92%; *p* < 0.001), with an ASDR of 1,929.21 per 100,000 population in 2021 (95% UI: 1284.02 to 2772.95). Central Latin America also showed a notable increase (AAPC = 1.52, 95%CI: 1.25 to 1.78%; *p* < 0.001), with an ASDR of 1,168.22 per 100,000 population in 2021 2021 (95% UI: 724.13 to 1736.5) (Table 1). Conversely, the lowest ASDR was in East Asia, with 560.51 per 100,000 population (95% UI: 365.8 to 799.17), followed by Southeast Asia with 683.77 per 100,000 population (95% UI: 434.95 to 1007.98), and Oceania with 720.59 per 100,000 population (95% UI: 430.31 to 1107.37) (Table 1).

### Depressive disorders among WCBA: national level

**Figure 2:**
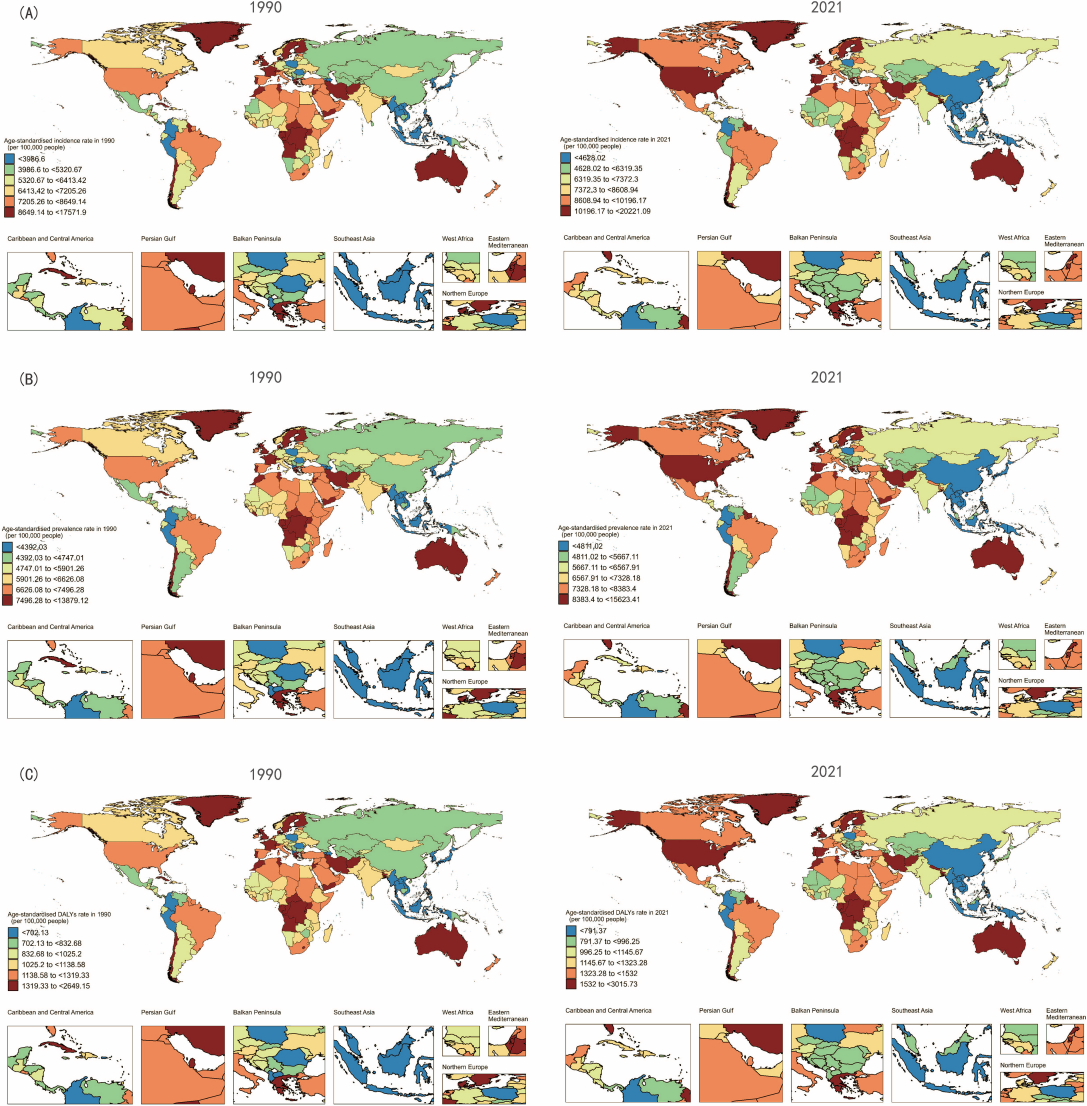
Maps showing **(A)** age-standardized incidence rate, **(B)** age-standardized prevalence rate and **(C)** age-standardized disability-adjusted life years rate of depression among women of childbearing age aged 15–49 years, in 204 countries and territories, between 1990 and 2021.

**Figure 3:**
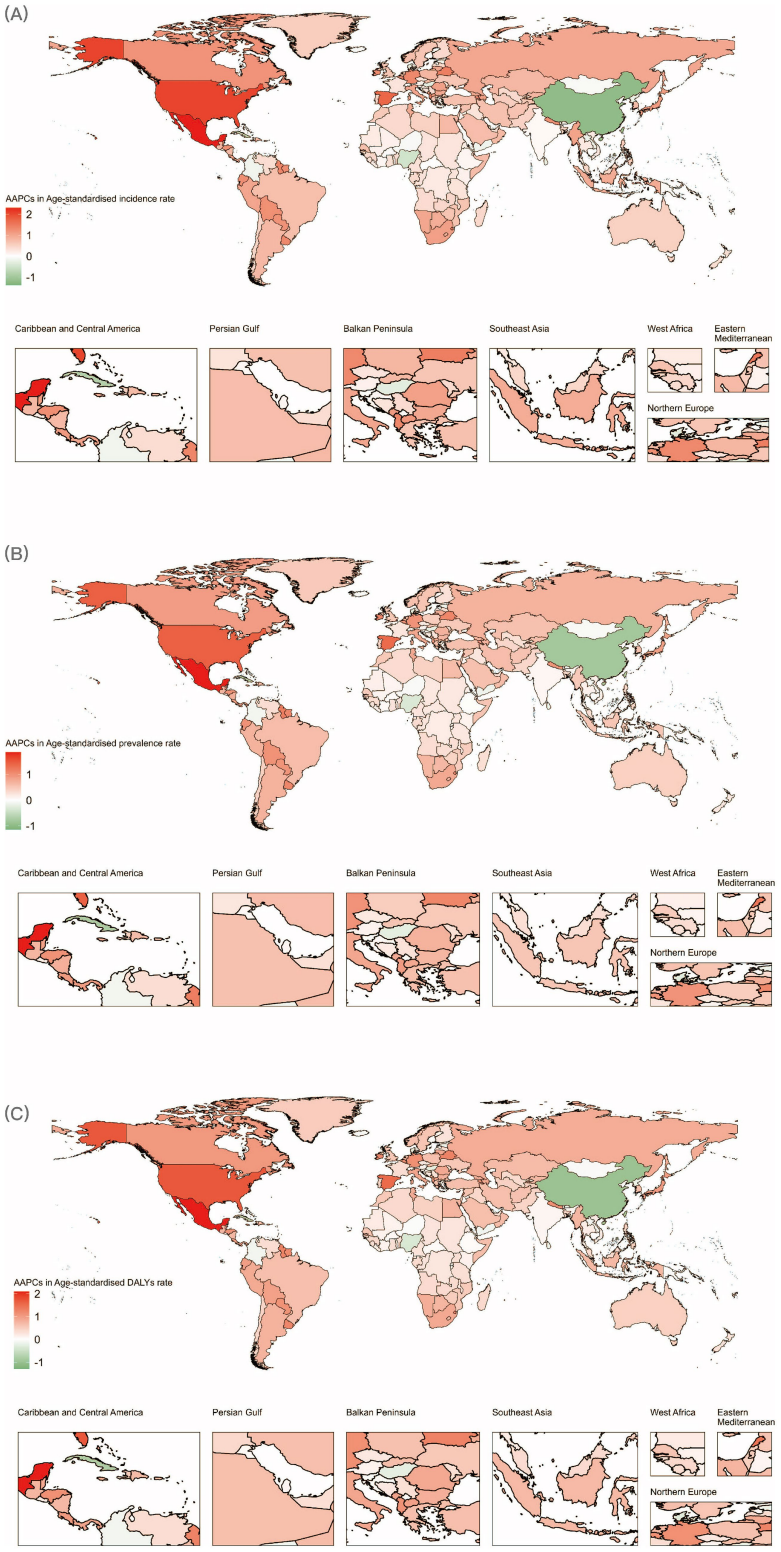
World map of AAPCs in **(A)** age-standardized incidence rate, **(B)** age-standardized prevalence rate and **(C)** age-standardized disability-adjusted life years rate of depression among women of childbearing age from 1990 to 2019. AAPC, average annual percentage change.

In 2021, the highest ASIR of depressive disorders among WCBA were reported in Greenland (20,221.09 per 100,000 population; 95% UI: 13654.57 to 28734.58), Lesotho (13,845.53 per 100,000 population; 95% UI: 9036.27 to 20293.29), Greece (13,841.31 per 100,000 population; 95% UI: 8548.39 to 21193.92), Guyana (13,100.09 per 100,000 population; 95% UI: 8472.65 to 19238.32), and the United States of America (13,072.89 per 100,000 population; 95% UI: 10148.83 to 16567.53) (Supplementary Table 1; Figure 2A). Conversely, the lowest ASIR was observed in Myanmar (2,717.48 per 100,000 population; 95% UI: 1709.34 to 4182.68), China (2,947.48 per 100,000 population; 95% UI: 2221.51 to 3825.84), Taiwan (Province of China) (3,058.11 per 100,000 population; 95% UI: 2055.78 to 4421.2), and the Democratic People’s Republic of Korea (3,255.02 per 100,000 population; 95% UI: 2157.79 to 4737.78) (Supplementary Table 1; Figure 2A). From 1990 to 2021, different countries exhibited varying changes in ASIR. Notably, Mexico (AAPC = 2.31, 95% CI: 2.15 to 2.47%; *p* < 0.001) and the United States of America (AAPC = 2.05, 95% CI: 1.55 to 2.55%; *p* < 0.001) had the most significant relative increases in ASIR (Supplementary Table 1; Figure 3A).

The national distribution of ASPR is detailed in Figure 3B and Supplementary Table 2. The highest ASPR in 2021 was in Greenland (15,623.41 per 100,000 population; 95% UI: 11143.73 to 21322.5), Lesotho (11,151.88 per 100,000 population; 95% UI: 7761.43 to 15442.99), and Greece (10,839 per 100,000 population; 95% UI: 7231.31 to 15717.3) (Supplementary Table 2; Figure 2B). Conversely, the lowest ASPR was observed in Brunei Darussalam (3,155.59 per 100,000 population; 95% UI: 2225.67 to 4444.72) and Colombia (3,221.5 per 100,000 population; 95% UI: 2316.72 to 4351.35) (Supplementary Table 2; Figure 2B). The increasing trend in ASPR from 1990 to 2021 was most evident in Mexico (AAPC = 1.86, 95% CI: 1.74 to 1.99%; *p* < 0.001), the United States of America (AAPC = 1.44, 95% CI: 1.27 to 1.61%; *p* < 0.001), and Spain (AAPC = 1.35, 95% CI: 1.04 to 1.67%; *p* < 0.001). The most significant decreasing trend was observed in Singapore (AAPC = −1.14, 95% CI: −1.29% to −1%; *p* < 0.001) (Supplementary Table 2; Figure 3B).

Regarding ASDR, in 2021, the highest rates were reported in Greenland (3,015.73 per 100,000 population; 95% UI: 1803.63 to 4729.04), Greece (2,062.84 per 100,000 population; 95% UI: 1154.22 to 3385.78), and Lesotho (2,030.26 per 100,000 population; 95% UI: 1190.98 to 3097.24) (Supplementary Table 3; Figure 2C). Conversely, the lowest ASDR was observed in Myanmar (525.32 per 100,000 population; 95% UI: 310.25 to 821.76), Colombia (547.77 per 100,000 population; 95% UI: 316.78 to 861.78), and Brunei Darussalam (550.2 per 100,000 population; 95% UI: 307.92 to 886.51) (Supplementary Table 3; Figure 2C). The increasing trend in ASDR from 1990 to 2021 was most pronounced in Mexico (AAPC = 2.09, 95% CI: 1.95 to 2.23%; *p* < 0.001), the United States of America (AAPC = 1.7, 95% CI: 1.37 to 2.03%; *p* < 0.001), and Spain (AAPC = 1.52, 95% CI: 1.24 to 1.81%; *p* < 0.001). Notably, Singapore exhibited a decreasing trend in ASDR (AAPC = −1.29, 95% CI: −1.56% to −1.02%; *p* < 0.001) (Supplementary Table 3; Figure 3C).

Moreover, over the past 30 years, the changes in ASIR, ASPR, and ASDR have varied across different countries and periods (Supplementary Tables 7–9). However, it is noteworthy that after 2019, a significant increase in the burden of depressive disorders has been observed in most countries and regions worldwide (Supplementary Tables 4–9).

### Temporal joinpoint analysis

Analysis of the four global super-regions with the highest global ASIR revealed a declining trend in high-income regions from 2001 to 2019 (relative APC = −0.18, 95% CI: −0.25% to −0.11%, *p* < 0.001), followed by a significant increase from 2019 to 2021 (relative APC = 16.33, 95% CI: 13.53 to 19.2%) (Supplementary Table 4; Figure 1). Similarly, a steady decline was seen in Latin America and the Caribbean from 2003 to 2018 (relative APC = −1%; 95%CI: −1.33% to −0.66%; *p* < 0.001) (Supplementary Table 4; Figure 1). In North Africa and the Middle East, a stable trend was observed from 1990 to 2018 (relative APC = −0.01, 95% CI: −0.1 to 0.08%, *p* > 0.05), followed by a significant increase from 2018 to 2021 (relative APC = 6.82, 95% CI: 3.53 to 10.22%; *p* < 0.001) (Supplementary Table 4; Figure 1). In contrast, a turning point was noted in Sub-Saharan Africa in 2019, with ASIR increasing (relative APC = 7.52, 95% CI: 6.54–8.51%; *p* < 0.001) (Supplementary Table 4; Figure 1). The overall trends for ASPR and ASDR in depressive disorders among WCBA were consistent with ASIR, showing a significant increase in all global super-regions after 2019 (Supplementary Tables 5, 6; Supplementary Figures 1, 2).

## Discussion

This study presents the burden of depressive disorders among WCBA globally across seven global super-regions, 21 regions, and 204 countries. It analyzes temporal trends and reveals differences between regions and countries. The results underscore a rapid increase in depressive disorders among WCBA in most regions worldwide after 2019. These results highlight the urgency for regions and countries to implement proactive measures to increase mental health care coverage and address the disease burden of depressive disorders among WCBA.

Compared to 2019, the global burden of depressive disorders among WCBA surged, reversing the downward trend seen over the past 30 years due to efforts to improve women’s socio-economic status. This reversal is likely linked to the COVID-19 pandemic, which spread rapidly worldwide from early 2020. Despite urgent public health and social measures taken to curb the spread of COVID-19, past studies have shown that populations experiencing fear during catastrophic events have a significantly higher risk of depressive disorders (21). Women, as a vulnerable group, are more prone to depressive disorders if they have difficulty regulating their emotions after trauma (22). Additionally, WCBA face unique mental health risks due to inherent gender conditions, hormonal patterns, immune system changes, and metabolism, leading to conditions such as pubertal depressive disorders, premenstrual syndrome, perinatal depression, and perimenopausal depression (23). Socioeconomic and sociocultural inequalities also increase the risk of depression (24). Although awareness of depressive disorders among women has increased over recent decades, our analysis indicates that understanding, diagnosing, and treating depressive disorders among WCBA remains inadequate post-COVID-19. With the potential socio-economic and cultural impacts of COVID-19, this issue is expected to worsen. While the COVID-19 pandemic undoubtedly amplified depressive disorder burdens globally, our findings reveal pre-existing and region-specific vulnerabilities. In high-income regions, rising burdens may reflect the paradoxical effects of socioeconomic development, where increased workforce participation among women coexists with unmet childcare support and workplace discrimination (25, 26). Conversely, in low- and middle-income countries, systemic underinvestment in mental health infrastructure—coupled with cultural stigma—likely perpetuates underdiagnosis and untreated chronic cases (27). In North Africa and the Middle East, socioeconomic disadvantage, gender discrimination, and unemployment are frequently reported risk factors for depressive symptoms among women (28). In addition, poor obstetric conditions, adverse birth, and infant health outcomes exacerbate the depressive disorders burden (29). Women aged 15–49 undergo significant physiological events such as sexual maturation, pregnancy, and childbirth. Women during this period might be more affected by female-specific risk factors than men. In low-income regions, poor marital relationships and economic deprivation are significant risk factors for both the onset and persistence of depressive disorders and should be primary targets for economic health interventions (30).

In contrast, China, representing East Asia, showed the largest decrease in ASIR. Early in the pandemic, China integrated psychological crisis intervention into its overall epidemic control measures, and women received increased family support (31, 32). Singapore exhibited the largest decrease in ASDR, attributed to substantial increases in healthcare spending, including the expansion and upgrading of public hospital facilities and long-term mental health services (33). These interventions contrast sharply with North Africa and the Middle East, where rigid gender roles and limited mental health funding exacerbate burdens. Furthermore, pre-pandemic trends suggest that regions with robust social safety nets experienced slower increases, whereas areas with fragmented systems (e.g., Latin America) faced compounding stressors (34). Mexico’s post-2019 surge, for example, mirrors pre-existing disparities in rural healthcare access, magnified by pandemic-related service disruptions (35). Our study also found considerable variability in the ASIR, ASPR, and age-standardized mortality rates across 204 countries and regions, indicating substantial heterogeneity in the burden of depressive disorders among WCBA worldwide. Therefore, it is imperative to implement immediate and concrete actions across healthcare systems. To effectively address this challenge, we propose the establishment of specialized mental health clinics in both urban and rural areas, along with telemedicine programs for underserved regions. Concurrently, treatment subsidy initiatives should be introduced for WCBA in low- and middle-income areas, while integrating routine mental health screening into prenatal and postnatal care (36). Anti-stigma campaigns should be promoted, workplace inequalities addressed, and digital health monitoring systems alongside regional registries implemented to enhance surveillance. Emphasis should be placed on post-pandemic mental health safeguards, with implementation following a defined timeline: surveillance systems and training programs within 2 years, comprehensive care pathways within 5 years, and measurable reductions in prevalence rates in high-burden regions beyond 5 years. This integrated approach combines targeted policy interventions with dedicated research initiatives, addressing the complex challenges identified in our study by considering both biological and social determinants in the management of depressive disorders among WCBA (10).

## Strengths and limitations

A major strength of this study is the precise estimation of age-standardized indicators for depressive disorders among WCBA, compensating for the heterogeneity of age structure and eliminating the confounding effect of age across different geographic areas. This finding facilitates effective comparisons and lays the foundation for further scientific research.

Several limitations should be noted. First, due to the poor mental health record systems in underdeveloped countries, the burden of depressive disorders in the GBD study might be underestimated. Second, the data lacked information on prenatal depression, postpartum depression, and menopausal depression. Future GBD research should include information to facilitate such classifications. Although GBD modeling techniques aim to standardize data, the extracted data may heavily rely on model-based estimates, which could impact accuracy.

## Conclusion

This study provides age-standardized estimates of the epidemiological indicators of the burden of depressive disorders among WCBA, facilitating effective comparisons across different regions. We found that the burden of depressive disorders among WCBA globally showed an increasing trend from 1990 to 2021. Notably, after 2019, there was a sharp increase in the burden of depressive disorders among WCBA in almost all countries and regions worldwide. Governments urgently need to develop flexible health policies and improve healthcare systems to mitigate the growing threat of depressive disorders among WCBA.

## Data Availability

The original contributions presented in the study are included in the article/Supplementary material, further inquiries can be directed to the corresponding author.

## Footnotes

^1^ https://vizhub.healthdata.org/gbd-results/

^2^ http://www.r-project.org
