# Supplementary material for: Global, regional, and national burden of depressive disorders among women of childbearing age, 1990–2021: a joinpoint regression analysis for the Global Burden of Disease Study 2021
Source: Front Public Health. 2025 May 15;13:1566240. doi: 10.3389/fpubh.2025.1566240 (PMC12119534; doi:10.3389/fpubh.2025.1566240)

Supplementary Material

Supplementary Table 1. Age-standardized incidence rate of depressive disorders in 1990 and 2021 for women of childbearing age in 204 countries, with AAPCs from 1990 and 2021. Abbreviations: ASIR, age-standardized incidence rate; UI, uncertainty interval; AAPC, average annual percent change; CI, confidence interval.

Supplementary Table 2. Age-standardized prevalence rate of depressive disorders in 1990 and 2021 for women of childbearing age in 204 countries, with AAPCs from 1990 and 2021. Abbreviations: ASPR, age-standardized prevalence rate; UI, uncertainty interval; AAPC, average annual percent change; CI, confidence interval.

Supplementary Table 3. Age-standardized DALYs rate of depressive disorders in 1990 and 2021 for women of childbearing age in 204 countries, with AAPCs from 1990 and 2021. Abbreviations: ASDR, age-standardized DALYs rate; UI, uncertainty interval; AAPC, average annual percent change; CI, confidence interval.

Supplementary Table 4. APCs of 7 super regions in ASIR of depressive disorders in WCBA. Abbreviations: WCBA, women of childbearing age; APC, annual percentage change; CI, confidence interval; ASIR, age-standardized incidence rate.

Supplementary Table 5. APCs of 7 super regions in ASPR of depressive disorders in WCBA. Abbreviations: WCBA, women of childbearing age; APC, annual percentage change; CI, confidence interval; ASPR, age-standardized prevalence rate.

Supplementary Table 6. APCs of 7 super regions in ASDR of depressive disorders in WCBA. Abbreviations: WCBA, women of childbearing age; APC, annual percentage change; CI, confidence interval; ASDR, age-standardized DALYs rate.

Supplementary Table 7. APCs of 204 countries in ASIR of depressive disorders in WCBA. Abbreviations: WCBA, women of childbearing age; APC, annual percentage change; CI, confidence interval; ASIR, age-standardized incidence rate.

Supplementary Table 8. APCs of 204 countries in ASPR of depressive disorders in WCBA. Abbreviations: WCBA, women of childbearing age; APC, annual percentage change; CI, confidence interval; ASPR, age-standardized prevalence rate.

Supplementary Table 9. APCs of 204 countries in ASDR of depressive disorders in WCBA. Abbreviations: WCBA, women of childbearing age; APC, annual percentage change; CI, confidence interval; ASDR, age-standardized DALYs rate.

Supplementary Fig. 1 Joinpoint regression analysis of age-standardized prevalence rate of depression among women of childbearing age at the global and four regions (High income, Latin America and Caribbean, North Africa and Middle East, Sub-Saharan Africa) from 1990 to 2021. P-value *P < 0.05.

Supplementary Fig. 2 Joinpoint regression analysis of age-standardized DALYs rate of depression among women of childbearing age at the global and four regions (High income, Latin America and Caribbean, North Africa and Middle East, Sub-Saharan Africa) from 1990 to 2021. P-value *P < 0.05.

Supplementary Table 1. Age-standardized incidence rate of depressive disorders in 1990 and 2021 for women of childbearing age in 204 countries, with AAPCs from 1990 and 2021. Abbreviations: ASIR, age-standardized incidence rate; UI, uncertainty interval; AAPC, average annual percent change; CI, confidence interval.

| location | ASIR, per 100,000 (95% UI) | | AAPC (95% CI) |
| --- | --- | --- | --- |
|  | 1990 | 2021 |  |
| Afghanistan | 9789.34 (6680.56 to 14037.36) | 11768.52 (7634.29 to 17317.93) | 0.67 (0.59 to 0.75) |
| Albania | 3628.64 (2460.52 to 5160.27) | 5277.31 (3397.48 to 8023.34) | 1.37 (1.21 to 1.53) |
| Algeria | 8078.23 (5309.81 to 11859.45) | 8896.84 (5535.07 to 13853.61) | 0.42 (0.18 to 0.67) |
| American Samoa | 2984.33 (2082.83 to 4181.73) | 3386.91 (2191.41 to 5058) | 0.38 (0.26 to 0.5) |
| Andorra | 7509.87 (5147.37 to 10737.52) | 9421.87 (5898.04 to 14408.85) | 0.91 (0.5 to 1.33) |
| Angola | 11259.98 (7705.44 to 16274.44) | 12452.72 (7995.46 to 18644.24) | 0.39 (0.24 to 0.55) |
| Antigua and Barbuda | 5203.16 (3422.64 to 7679.03) | 6740.22 (4173.29 to 10343.99) | 0.9 (0.86 to 0.94) |
| Argentina | 5470.55 (3809.14 to 7735.45) | 6744.09 (4777.34 to 9312.56) | 0.8 (0.4 to 1.2) |
| Armenia | 3837.79 (2592.65 to 5513.56) | 5478.11 (3438.51 to 8360.33) | 1.35 (1.16 to 1.54) |
| Australia | 9273.68 (7075.4 to 11894.28) | 10655.35 (7063.86 to 15206.69) | 0.53 (0.43 to 0.62) |
| Austria | 7151.91 (5102.17 to 9834.51) | 7277.52 (4763.02 to 10693.65) | 0.15 (-0.06 to 0.37) |
| Azerbaijan | 3824.06 (2578.15 to 5542.45) | 5198.02 (3254.37 to 7833.49) | 1.08 (0.98 to 1.18) |
| Bahamas | 5246.74 (3457.88 to 7674.4) | 7015 (4370.79 to 10942.42) | 1.13 (0.9 to 1.37) |
| Bahrain | 10136.38 (6938.12 to 14275.73) | 10918.27 (6995.23 to 16393.05) | 0.31 (0.23 to 0.4) |
| Bangladesh | 9216.86 (6415.51 to 12894.38) | 10285.19 (6747.07 to 15097.48) | 0.5 (0.41 to 0.58) |
| Barbados | 5192.45 (3424.9 to 7610.32) | 7171.97 (4471.91 to 11152.53) | 1.06 (0.98 to 1.14) |
| Belarus | 6148.13 (4292.32 to 8445.64) | 9514.3 (6209.17 to 13916) | 1.45 (1.4 to 1.49) |
| Belgium | 6460.3 (5320.32 to 7835.35) | 8949.29 (5908.1 to 13159.12) | 1.27 (0.68 to 1.87) |
| Belize | 5320.68 (3534.05 to 7855.26) | 6906.33 (4397.18 to 10526.6) | 1.09 (0.85 to 1.33) |
| Benin | 6513.85 (4473.65 to 9429.81) | 7187.46 (4625.16 to 10903.52) | 0.37 (0.31 to 0.43) |
| Bermuda | 6369.58 (4427.23 to 9078.89) | 7062.48 (4310.56 to 10801.62) | 0.39 (0.34 to 0.44) |
| Bhutan | 6797.43 (4688.33 to 9657.02) | 6236.3 (3945.5 to 9425.16) | -0.18 (-0.32 to -0.03) |
| Bolivia (Plurinational State of) | 6681.26 (4629.36 to 9516.46) | 8903.55 (5755.43 to 13307.36) | 1.25 (1.1 to 1.41) |
| Bosnia and Herzegovina | 5496.67 (3850.86 to 7710.13) | 5889.63 (3630.26 to 8890.22) | 0.31 (0.2 to 0.41) |
| Botswana | 6407.73 (4409.33 to 9157.88) | 8683.77 (5618.04 to 13054.18) | 0.92 (0.8 to 1.05) |
| Brazil | 8350.53 (6337.44 to 10915.98) | 9772.37 (7286.12 to 12828.54) | 0.73 (0.28 to 1.18) |
| Brunei Darussalam | 2817.26 (1889.74 to 4092.98) | 3383.89 (2135.05 to 5255.84) | 0.65 (0.56 to 0.73) |
| Bulgaria | 4551.81 (3257.62 to 6321.82) | 6120.75 (3942.92 to 9174.25) | 0.98 (0.91 to 1.05) |
| Burkina Faso | 6251.96 (4319.15 to 8845.19) | 6407.15 (4148.46 to 9659.26) | 0.06 (-0.36 to 0.47) |
| Burundi | 8439.76 (5696.39 to 11941.38) | 8064.43 (5114.14 to 12279.66) | -0.09 (-0.15 to -0.02) |
| Cabo Verde | 6286.69 (4261.78 to 9110.39) | 8533.65 (5407.55 to 13037.59) | 1.08 (0.85 to 1.3) |
| Cambodia | 4413.87 (3060.6 to 6257.73) | 4485.28 (2941.86 to 6604.57) | 0 (-0.11 to 0.1) |
| Cameroon | 6859.32 (4690 to 9873.78) | 7532.16 (4803.66 to 11355.52) | 0.33 (0.3 to 0.36) |
| Canada | 6505.12 (4953.6 to 8609.49) | 9096.42 (6066.77 to 13261.55) | 1.21 (1.09 to 1.33) |
| Central African Republic | 11184.32 (7597.35 to 16123.72) | 11824.85 (7626.16 to 17910.14) | -0.01 (-0.11 to 0.1) |
| Chad | 8105.08 (5520.02 to 11813.21) | 8575.24 (5462.63 to 12744.3) | 0.3 (0.09 to 0.52) |
| Chile | 10548.91 (8335.82 to 13280.88) | 11524.79 (7463.88 to 17023.93) | 0.48 (0.36 to 0.6) |
| China | 4461.64 (3407.01 to 5789.15) | 2947.48 (2221.51 to 3825.84) | -1.19 (-1.57 to -0.8) |
| Colombia | 3661.16 (2483.83 to 5326.32) | 3409.81 (2207.17 to 5071.72) | -0.15 (-0.6 to 0.3) |
| Comoros | 6036.27 (4136.44 to 8642.49) | 6884.57 (4351.6 to 10431.66) | 0.5 (0.43 to 0.58) |
| Congo | 11166.76 (7617.65 to 16003.27) | 12064.56 (7724.91 to 18394.7) | 0.31 (-0.06 to 0.68) |
| Cook Islands | 4014.99 (2590.55 to 6145.59) | 4628.32 (2799.75 to 7367.31) | 0.44 (0.36 to 0.51) |
| Costa Rica | 5320.67 (3544.46 to 7734.65) | 7234.43 (4580.38 to 10979.93) | 1.14 (1 to 1.28) |
| Coted'Ivoire | 5652.57 (3902.16 to 8024.77) | 5891.69 (3820.1 to 8793.18) | 0.09 (0.01 to 0.17) |
| Croatia | 5395.2 (3842.86 to 7337.58) | 5421.75 (3568.93 to 8063.19) | 0.1 (0.05 to 0.15) |
| Cuba | 10320.08 (7465.85 to 13883.76) | 8038.92 (5199.22 to 12075.83) | -0.82 (-0.92 to -0.71) |
| Cyprus | 6183.27 (4051.53 to 9080) | 7549.47 (4646.92 to 11685.18) | 0.7 (0.62 to 0.79) |
| Czechia | 5290.94 (3769.49 to 7317.59) | 5708.58 (3643.19 to 8498.6) | 0.3 (0.21 to 0.4) |
| Democratic People's Republic of Korea | 3501.89 (2507.49 to 4848.56) | 3255.02 (2157.79 to 4737.78) | -0.23 (-0.25 to -0.21) |
| Democratic Republic of the Congo | 10403.1 (7184.81 to 14830.23) | 10731.63 (6910.47 to 15791.67) | 0.24 (0.01 to 0.47) |
| Denmark | 9606.6 (6916.83 to 13124.53) | 8551.74 (5513.98 to 12668.25) | -0.3 (-0.36 to -0.24) |
| Djibouti | 6352.84 (4257.51 to 9306.99) | 7351.61 (4708.6 to 11019.99) | 0.51 (0.35 to 0.68) |
| Dominica | 5219.59 (3420.48 to 7749) | 6889.17 (4269.82 to 10616.38) | 0.95 (0.89 to 1.01) |
| Dominican Republic | 6930.5 (4621.28 to 10139.31) | 8246.8 (5106.45 to 12731.05) | 0.77 (0.32 to 1.22) |
| Ecuador | 5771.56 (3941.76 to 8155.76) | 7747.17 (4998.46 to 11570.55) | 1.26 (1.16 to 1.37) |
| Egypt | 7183.65 (4729.47 to 10597.53) | 8949.98 (5581.89 to 13627.29) | 0.78 (0.63 to 0.93) |
| El Salvador | 7718.14 (5361.58 to 10947.37) | 8065.43 (5232.67 to 11962.43) | 0.21 (-0.15 to 0.58) |
| Equatorial Guinea | 11358.42 (7768.2 to 16336.75) | 12062.3 (7744.19 to 18264.46) | 0.29 (0.06 to 0.52) |
| Eritrea | 7242.49 (4979.64 to 10269.15) | 7719.67 (4963.5 to 11714.45) | 0.21 (0.2 to 0.22) |
| Estonia | 8014.39 (5701.91 to 11007.15) | 7753.19 (4983.9 to 11625.24) | -0.16 (-0.24 to -0.08) |
| Eswatini | 6330.13 (4348.34 to 8892.48) | 10352.94 (6655.63 to 15376.45) | 1.58 (1.46 to 1.71) |
| Ethiopia | 6923.76 (4968.39 to 9447.47) | 7061.36 (4998.41 to 9814.81) | 0.11 (0.02 to 0.2) |
| Fiji | 3548.9 (2521.57 to 5002.91) | 4334.15 (2868.97 to 6392.57) | 0.6 (0.57 to 0.63) |
| Finland | 10400.92 (7451.35 to 13989.86) | 11190.88 (7432.99 to 16114.85) | 0.43 (-0.23 to 1.09) |
| France | 9517.04 (7864.16 to 11387.59) | 10135.1 (6704.6 to 14817.77) | 0.3 (-0.12 to 0.72) |
| Gabon | 10100.05 (6913.27 to 14598.17) | 11365.03 (7322.95 to 16744.55) | 0.42 (0.3 to 0.53) |
| Gambia | 11604.87 (7945.32 to 16567.55) | 12222.79 (7783.47 to 18335.83) | 0.23 (0.18 to 0.29) |
| Georgia | 4325.25 (2920.33 to 6246.26) | 5874.04 (3647.32 to 8910.94) | 1.01 (0.97 to 1.04) |
| Germany | 5674.77 (4418.2 to 7317.31) | 8403.43 (5429.43 to 12482.42) | 1.33 (0.76 to 1.91) |
| Ghana | 6510.17 (4359.81 to 9314.59) | 7137.41 (4447.38 to 10872.54) | 0.35 (0.19 to 0.52) |
| Greece | 10634.79 (6957.54 to 15659.78) | 13841.31 (8548.39 to 21193.92) | 0.67 (-0.01 to 1.35) |
| Greenland | 17571.9 (12718.38 to 24179.26) | 20221.09 (13654.57 to 28734.58) | 0.57 (0.43 to 0.7) |
| Grenada | 5330.07 (3503.38 to 7898.55) | 6865.95 (4278.57 to 10291.78) | 0.86 (0.81 to 0.9) |
| Guam | 3661.74 (2585.44 to 5047.96) | 4543.96 (3017.32 to 6716.16) | 0.77 (0.67 to 0.87) |
| Guatemala | 6870.7 (4672.41 to 10034.64) | 8574.38 (5450.88 to 13128.48) | 0.8 (0.43 to 1.16) |
| Guinea | 6690.14 (4604.89 to 9637.47) | 7618.44 (4912.78 to 11554.82) | 0.44 (0.31 to 0.57) |
| Guinea-Bissau | 6929.13 (4759.79 to 9953.43) | 7824.75 (5028.24 to 11786.72) | 0.44 (0.27 to 0.61) |
| Guyana | 8716.48 (6036.83 to 12097.62) | 13100.09 (8472.65 to 19238.32) | 1.37 (1.31 to 1.43) |
| Haiti | 6954.94 (4699.64 to 10190.86) | 8115.46 (5156.15 to 12135.23) | 0.55 (0.44 to 0.67) |
| Honduras | 5198.19 (3443.86 to 7546.01) | 7256.81 (4544.9 to 11043.44) | 1.26 (1.16 to 1.37) |
| Hungary | 5556.22 (4005.17 to 7576.77) | 5083.24 (3306.8 to 7396.93) | -0.28 (-0.32 to -0.24) |
| Iceland | 6547.03 (4613.74 to 9058.2) | 6380.2 (4063.05 to 9601.82) | -0.07 (-0.35 to 0.2) |
| India | 6893.72 (5182.5 to 9090.56) | 6820.95 (5105.71 to 9002.1) | 0.08 (-0.08 to 0.23) |
| Indonesia | 2760.81 (2002.69 to 3712.98) | 3651.76 (2605.14 to 5012.05) | 0.94 (0.87 to 1.01) |
| Iran (Islamic Republic of) | 10405.37 (7334.13 to 14572.62) | 12267.94 (8681.08 to 16991.17) | 0.7 (0.42 to 0.98) |
| Iraq | 7812.54 (5417.05 to 10859.07) | 8548.05 (5492.56 to 12589.97) | 0.31 (-0.82 to 1.45) |
| Ireland | 8048.58 (5916.86 to 10727.34) | 11604.98 (7585.28 to 16917.48) | 1.32 (1.05 to 1.59) |
| Israel | 8700.12 (5974.04 to 12387.57) | 9945.83 (6227.13 to 15043.32) | 0.54 (0.43 to 0.64) |
| Italy | 7670.64 (5484.23 to 10487.13) | 9911.36 (6986.7 to 13625.15) | 1 (0.89 to 1.12) |
| Jamaica | 5283.87 (3458.7 to 7839.95) | 7060.54 (4306.67 to 11057.66) | 0.95 (0.9 to 1) |
| Japan | 3813.61 (2946.08 to 4923.21) | 5301.71 (4040.35 to 6907.97) | 1.14 (0.88 to 1.39) |
| Jordan | 9330.6 (6291.26 to 13609.56) | 9579.58 (6047 to 14577.74) | 0.13 (0.05 to 0.21) |
| Kazakhstan | 4914.52 (3505.51 to 6784.91) | 6190.95 (4086.88 to 9019.6) | 0.8 (0.44 to 1.16) |
| Kenya | 7277.69 (5431.17 to 9613.68) | 7469.66 (5523.54 to 9951.2) | 0.16 (-0.1 to 0.42) |
| Kiribati | 3709.89 (2633.98 to 5178.69) | 3727.57 (2460.32 to 5539.51) | -0.01 (-0.07 to 0.05) |
| Kuwait | 8037.4 (5333.48 to 11776.6) | 7980.21 (4940.59 to 12300.8) | 0.09 (-0.18 to 0.35) |
| Kyrgyzstan | 5176.8 (3625.47 to 7112.21) | 6360.5 (4180.36 to 9348.04) | 0.83 (0.69 to 0.97) |
| Lao People's Democratic Republic | 3406.51 (2362.09 to 4849.49) | 3293.3 (2158.67 to 4914.31) | -0.18 (-0.94 to 0.58) |
| Latvia | 6769.86 (4788.94 to 9267.69) | 8041.18 (5092.84 to 12129.28) | 0.45 (0.23 to 0.67) |
| Lebanon | 7984.27 (5623.06 to 11231.11) | 12984.07 (8110.04 to 19808.95) | 1.55 (1.4 to 1.69) |
| Lesotho | 10431.48 (7174.22 to 14915.14) | 13845.53 (9036.27 to 20293.29) | 0.89 (0.84 to 0.94) |
| Liberia | 8638.94 (5917.77 to 12363.72) | 9029.02 (5790.19 to 13612.88) | 0.23 (0.19 to 0.27) |
| Libya | 8321.52 (5514.64 to 12123.78) | 9530.68 (6032.16 to 14295.7) | 0.53 (0.28 to 0.78) |
| Lithuania | 7019.96 (4977.25 to 9660.25) | 9518.64 (6285.07 to 13931.49) | 0.98 (0.92 to 1.05) |
| Luxembourg | 8021 (5623.06 to 10956.24) | 7499.55 (5088.38 to 10686.36) | -0.12 (-0.38 to 0.14) |
| Madagascar | 6979.22 (4796.06 to 9984.12) | 7814.61 (4930.72 to 11745.92) | 0.44 (0.33 to 0.54) |
| Malawi | 6179.38 (4247.09 to 8743.22) | 6750.94 (4362.82 to 10269.48) | 0.28 (0.25 to 0.31) |
| Malaysia | 3868.68 (2687.28 to 5472.37) | 4749.72 (3011.71 to 7175.43) | 0.45 (-0.3 to 1.21) |
| Maldives | 5867.65 (4135.11 to 8166.81) | 4893.22 (3202.39 to 7172.02) | -0.55 (-0.61 to -0.48) |
| Mali | 5366.71 (3699.57 to 7640.57) | 5498.16 (3586.76 to 8394.82) | 0.15 (0.07 to 0.23) |
| Malta | 6190.68 (4044.57 to 9018.07) | 7393 (4558.34 to 11357.36) | 0.78 (0.51 to 1.05) |
| Marshall Islands | 3483.05 (2442.79 to 4889.65) | 3802.65 (2472.15 to 5630.04) | 0.26 (0.19 to 0.33) |
| Mauritania | 4938.37 (3414.09 to 6975.67) | 5227.11 (3351.12 to 7927.7) | 0.24 (0.14 to 0.34) |
| Mauritius | 7459.72 (5226.81 to 10244.85) | 8044.69 (5198.57 to 11932.65) | 0.34 (0.04 to 0.63) |
| Mexico | 5309.93 (3887.06 to 7090.62) | 9961.29 (7358.15 to 13268.44) | 2.31 (2.15 to 2.47) |
| Micronesia (Federated States of) | 3611.13 (2537.51 to 5100.37) | 3756.49 (2456.97 to 5529.23) | 0.1 (0.03 to 0.17) |
| Monaco | 8343.53 (5297.83 to 12744.18) | 10882.45 (6591.3 to 17163.88) | 0.93 (0.9 to 0.97) |
| Mongolia | 6603.4 (4636.14 to 9251.83) | 6569.04 (4380.85 to 9668.5) | -0.07 (-0.19 to 0.05) |
| Montenegro | 4457.71 (3129.26 to 6135.65) | 6178.86 (4032.62 to 9178.51) | 1.14 (1.08 to 1.2) |
| Morocco | 9975.05 (6841.02 to 14254.01) | 11687.82 (7437.82 to 17385.89) | 0.6 (0.24 to 0.97) |
| Mozambique | 6790.43 (4628.44 to 9801.32) | 8365.67 (5330.74 to 12793.46) | 0.66 (0.64 to 0.69) |
| Myanmar | 1950.68 (1317.23 to 2785.01) | 2717.48 (1709.34 to 4182.68) | 1.04 (0.66 to 1.42) |
| Namibia | 5240.43 (3653.09 to 7433.1) | 7330.27 (4689.58 to 11169.36) | 1 (0.58 to 1.42) |
| Nauru | 3978.8 (2557.41 to 6015.18) | 4611.02 (2812.46 to 7356.6) | 0.44 (0.37 to 0.51) |
| Nepal | 7201.36 (5030.5 to 10032.1) | 10351.69 (6669.64 to 15459.54) | 1.24 (0.71 to 1.78) |
| Netherlands | 7614.49 (6269.91 to 9258.52) | 9183.95 (5916.85 to 13526.73) | 0.8 (0.59 to 1) |
| New Zealand | 7508.81 (5254.85 to 10418.74) | 8307.46 (5853.54 to 11713.79) | 0.36 (0.22 to 0.51) |
| Nicaragua | 5945.15 (4051.73 to 8539.81) | 7424.67 (4783.63 to 11102.99) | 0.78 (0.4 to 1.16) |
| Niger | 6569.2 (4518.36 to 9432.87) | 6471.06 (4176.29 to 9624.15) | -0.08 (-0.13 to -0.02) |
| Nigeria | 6209.15 (4451.97 to 8515.75) | 5211.87 (3755.48 to 7077.52) | -0.48 (-0.75 to -0.22) |
| Niue | 3987.89 (2568.47 to 6023.01) | 4604.14 (2832.58 to 7206.48) | 0.45 (0.37 to 0.53) |
| North Macedonia | 4078.19 (2879.48 to 5734.23) | 5686.53 (3650.17 to 8496.58) | 1.17 (1.02 to 1.32) |
| Northern Mariana Islands | 2837.51 (1926.52 to 4122.79) | 3773.04 (2445.55 to 5680.18) | 0.92 (0.83 to 1.01) |
| Norway | 7096.98 (5126.21 to 9628.44) | 9022.03 (6330.77 to 12417.36) | 0.73 (0.36 to 1.11) |
| Oman | 7675.64 (5065.46 to 11239.05) | 9332.76 (5734.78 to 14444.06) | 0.78 (0.71 to 0.85) |
| Pakistan | 6849.92 (4856.6 to 9368.96) | 7492.72 (5179.46 to 10439.18) | 0.49 (0.13 to 0.85) |
| Palau | 3996.71 (2590.4 to 6079.69) | 4626.5 (2824.34 to 7360.24) | 0.44 (0.36 to 0.53) |
| Palestine | 11149.71 (7414.35 to 16287.39) | 12928.12 (8113.95 to 19762.78) | 0.59 (0.45 to 0.73) |
| Panama | 5044.94 (3372.39 to 7421.16) | 6560.25 (4096.23 to 10164.68) | 1.01 (0.56 to 1.46) |
| Papua New Guinea | 4149.45 (2849.66 to 5923.22) | 4124.73 (2666.96 to 6188.47) | -0.05 (-0.13 to 0.04) |
| Paraguay | 6337.03 (4306.93 to 9189.43) | 9064.1 (5787.36 to 13516.77) | 1.23 (1.13 to 1.33) |
| Peru | 3703.19 (2510.32 to 5289.96) | 4791.79 (3044.95 to 7222.38) | 1 (0.31 to 1.7) |
| Philippines | 3851.5 (2769.33 to 5258.1) | 4480.85 (3170.63 to 6115.64) | 0.59 (0.48 to 0.69) |
| Poland | 2716.88 (1965.12 to 3662.61) | 3416.86 (2419.65 to 4657.49) | 0.81 (0.72 to 0.91) |
| Portugal | 11409.26 (7885.53 to 15717.8) | 13053.77 (8371.18 to 19763.49) | 0.51 (-0.39 to 1.42) |
| Puerto Rico | 4185.4 (2850.09 to 5938.85) | 5188.72 (3271.74 to 7898.84) | 0.79 (0.47 to 1.11) |
| Qatar | 9037.63 (6098.97 to 12807.31) | 9152.24 (5647.75 to 14060.42) | 0.1 (-0.16 to 0.37) |
| Republic of Korea | 3683.99 (2810.92 to 4832.65) | 4521.32 (3067.71 to 6360.41) | 0.77 (0.62 to 0.91) |
| Republic of Moldova | 5732.93 (4052.84 to 7835.31) | 6324.9 (4077.36 to 9560.63) | 0.41 (0.31 to 0.52) |
| Romania | 3947.8 (2726.37 to 5528.55) | 5298.94 (3426.71 to 7924.2) | 1.06 (0.93 to 1.18) |
| Russian Federation | 4727.57 (3387.74 to 6445.91) | 6361.07 (4493.17 to 8707.37) | 0.99 (0.52 to 1.45) |
| Rwanda | 9457.59 (6421.01 to 13497.28) | 9386.64 (5892.59 to 14061.05) | -0.04 (-0.15 to 0.06) |
| Saint Kitts and Nevis | 6955.74 (4418.5 to 10699.38) | 8676.35 (5159.86 to 13903.22) | 0.81 (0.73 to 0.89) |
| Saint Lucia | 5354.36 (3564.16 to 7881.92) | 7524.26 (4697.34 to 11492.27) | 1.11 (1.04 to 1.18) |
| Saint Vincent and the Grenadines | 5325.84 (3529.55 to 7832.03) | 7082.98 (4448.9 to 10887) | 0.96 (0.91 to 1) |
| Samoa | 3425.13 (2400.27 to 4799.25) | 3630.01 (2377.9 to 5408.67) | 0.15 (0.07 to 0.22) |
| San Marino | 8390.97 (5344.07 to 12913.41) | 11099.34 (6632.41 to 17766.93) | 1.14 (1.09 to 1.19) |
| Sao Tome and Principe | 5710.88 (3842.38 to 8204.18) | 6264.1 (3964.62 to 9505.49) | 0.36 (0.22 to 0.5) |
| Saudi Arabia | 7763.08 (5080.87 to 11454.47) | 9053.13 (5688.05 to 13810.61) | 0.74 (0.36 to 1.12) |
| Senegal | 5386.08 (3702.25 to 7792.83) | 6525.31 (4229.93 to 9856.89) | 0.7 (0.52 to 0.89) |
| Serbia | 4818.57 (3448.93 to 6586.16) | 5346.2 (3447.84 to 8088.42) | 0.4 (0.31 to 0.5) |
| Seychelles | 2884.06 (1979.3 to 4071.33) | 3800.17 (2360.31 to 5814.33) | 0.88 (0.81 to 0.94) |
| Sierra Leone | 6419.11 (4353.51 to 9153.53) | 7101.63 (4585.67 to 10802.79) | 0.38 (0.3 to 0.45) |
| Singapore | 6469.95 (5068.27 to 8369.84) | 4201.22 (2848.65 to 6054.3) | -1.38 (-1.65 to -1.1) |
| Slovakia | 4431.95 (3104.06 to 6192.74) | 5749.76 (3655.23 to 8819.82) | 0.79 (0.49 to 1.08) |
| Slovenia | 6561.32 (4769.27 to 8999.98) | 6308.26 (4157.48 to 9356.59) | -0.02 (-0.18 to 0.13) |
| Solomon Islands | 3980.18 (2756.94 to 5673.71) | 4193.92 (2732.19 to 6280.51) | 0.14 (0.1 to 0.18) |
| Somalia | 7410.09 (5042.15 to 10535.81) | 9310.93 (5904.35 to 14002.06) | 0.75 (0.67 to 0.83) |
| South Africa | 7295.43 (5599.36 to 9443.19) | 9495.58 (7127.29 to 12514.1) | 1.07 (0.69 to 1.45) |
| South Sudan | 7054.65 (4875.64 to 10077.4) | 7674.61 (4909.01 to 11744.75) | 0.36 (0.19 to 0.54) |
| Spain | 8145.56 (6669.62 to 9948.59) | 12605.74 (8310.49 to 18246.4) | 1.72 (1.28 to 2.17) |
| Sri Lanka | 4957.52 (3618.11 to 6622.27) | 4659.68 (3111.22 to 6723.81) | -0.24 (-0.47 to -0.01) |
| Sudan | 8271.09 (5572.58 to 11964.18) | 9078.98 (5777.34 to 13814.97) | 0.29 (-0.01 to 0.6) |
| Suriname | 8501.93 (5895.97 to 11805.7) | 12614.94 (8313.4 to 18575.15) | 1.28 (1.16 to 1.41) |
| Sweden | 9978.83 (7452.53 to 12938.8) | 12110.76 (8562.56 to 16591.38) | 0.64 (0.25 to 1.04) |
| Switzerland | 9290.65 (6759.2 to 12489.64) | 9228.46 (6015.91 to 13664.3) | 0 (-0.14 to 0.15) |
| Syrian Arab Republic | 7631.03 (5002 to 11298.28) | 9123.93 (5670.47 to 14052.13) | 0.69 (0.57 to 0.81) |
| Taiwan (Province of China) | 2504.37 (1791.38 to 3473.82) | 3058.11 (2055.78 to 4421.2) | 0.65 (0.59 to 0.71) |
| Tajikistan | 4360.19 (3000.41 to 6207) | 5232.75 (3328.68 to 7858.75) | 0.62 (0.51 to 0.74) |
| Thailand | 3562.37 (2525.34 to 4880.04) | 3877.74 (2561.76 to 5761.46) | 0.25 (0.21 to 0.28) |
| Timor-Leste | 3964.39 (2757.97 to 5664.41) | 3924.64 (2548.53 to 5841.05) | -0.07 (-0.13 to 0) |
| Togo | 6636.3 (4567.39 to 9372.69) | 7031.19 (4487.14 to 10429.25) | 0.23 (0.07 to 0.39) |
| Tokelau | 3996.78 (2578.95 to 6027.39) | 4603.13 (2804.37 to 7457.53) | 0.43 (0.35 to 0.51) |
| Tonga | 3271.03 (2299.93 to 4530.76) | 3547.69 (2334.96 to 5312.05) | 0.24 (0.17 to 0.3) |
| Trinidad and Tobago | 7448.38 (5149.74 to 10461.98) | 9624.7 (6190.97 to 14576.45) | 0.76 (0.65 to 0.88) |
| Tunisia | 9488.41 (6296.39 to 13749) | 12741.92 (7963.54 to 19504.28) | 0.96 (0.88 to 1.04) |
| Turkey | 8160.31 (6424.61 to 10285.35) | 9883.9 (6161.24 to 15273.07) | 0.7 (0.41 to 1) |
| Turkmenistan | 4653.35 (3256.96 to 6530) | 5372.26 (3414.06 to 8168.81) | 0.55 (0.5 to 0.61) |
| Tuvalu | 3967.59 (2565.93 to 5996.1) | 4556.94 (2778.74 to 7096.99) | 0.42 (0.35 to 0.48) |
| Uganda | 10712.63 (7403.84 to 15122.87) | 11746.52 (7523.46 to 17565.04) | 0.31 (0.14 to 0.47) |
| Ukraine | 6846.73 (4854.54 to 9453.38) | 8414.91 (5606.07 to 12112.71) | 0.64 (0.39 to 0.88) |
| United Arab Emirates | 7854.95 (5369.6 to 11338.88) | 8526.54 (5378.82 to 13047.89) | 0.38 (0.34 to 0.42) |
| United Kingdom | 9315.39 (6767.09 to 12626.22) | 10342.25 (7445.83 to 14054.11) | 0.63 (0.2 to 1.05) |
| United Republic of Tanzania | 7010.35 (4829.99 to 10099.83) | 7641.62 (4866.57 to 11461.41) | 0.31 (0.27 to 0.34) |
| United States of America | 7213.07 (5590.43 to 9278.62) | 13072.89 (10148.83 to 16567.53) | 2.05 (1.55 to 2.55) |
| United States Virgin Islands | 5403.05 (3589.21 to 7989.07) | 6931.32 (4399.08 to 10377.58) | 0.87 (0.72 to 1.02) |
| Uruguay | 5923.73 (4073.14 to 8441.73) | 8980.01 (5900.93 to 13210.89) | 1.34 (0.92 to 1.76) |
| Uzbekistan | 4829.59 (3391.91 to 6726.56) | 5359.03 (3477.34 to 8010.52) | 0.42 (0.26 to 0.58) |
| Vanuatu | 3906.05 (2730.67 to 5552.27) | 4111.52 (2671 to 6173.34) | 0.14 (0.06 to 0.22) |
| Venezuela (Bolivarian Republic of) | 5389.74 (3626.29 to 7838.34) | 6080.45 (3869.75 to 9279.43) | 0.39 (0.23 to 0.56) |
| Viet Nam | 3323.51 (2345.88 to 4579.71) | 3611.83 (2367.49 to 5348.51) | 0.26 (-0.01 to 0.53) |
| Yemen | 10228.77 (6884.55 to 14719.87) | 10178.37 (6386.74 to 15268.16) | -0.15 (-0.21 to -0.09) |
| Zambia | 5724.12 (3958.31 to 8132.4) | 6663.73 (4313.99 to 9954.08) | 0.46 (0.4 to 0.52) |
| Zimbabwe | 4168.63 (2827.54 to 5980.68) | 5148.42 (3311.47 to 7791.5) | 0.66 (0.43 to 0.89) |

Supplementary Table 2. Age-standardized prevalence rate of depressive disorders in 1990 and 2021 for women of childbearing age in 204 countries, with AAPCs from 1990 and 2021. Abbreviations: ASPR, age-standardized prevalence rate; UI, uncertainty interval; AAPC, average annual percent change; CI, confidence interval.

| location | ASPR, per 100,000 (95% UI) | | | AAPC (95% CI) |
| --- | --- | --- | --- | --- |
|  | 1990 | 2021 | |  |
| Afghanistan | 8161.04 (5956.71 to 11094.95) | | 9473.15 (6571.78 to 13211.84) | 0.54 (0.49 to 0.6) |
| Albania | 3918.81 (2962.84 to 5149.96) | | 5007.92 (3617.29 to 6901.25) | 0.89 (0.8 to 0.98) |
| Algeria | 7005.5 (5041.97 to 9552.43) | | 7546.09 (5247.49 to 10831.37) | 0.32 (0.13 to 0.51) |
| American Samoa | 3731.67 (2814.92 to 4927.84) | | 3996.43 (2949.59 to 5364.64) | 0.2 (0.15 to 0.26) |
| Andorra | 6544.43 (4837.52 to 8820.05) | | 7814.89 (5386.18 to 11110.14) | 0.71 (0.39 to 1.02) |
| Angola | 9394.68 (6839.44 to 12691.61) | | 10169.56 (7161.37 to 14313.7) | 0.31 (0.18 to 0.43) |
| Antigua and Barbuda | 4617.43 (3310.74 to 6290.62) | | 5631.91 (3898.74 to 8051.56) | 0.68 (0.65 to 0.72) |
| Argentina | 4681.76 (3429.54 to 6247.08) | | 5550.91 (4107.02 to 7319.81) | 0.69 (0.59 to 0.8) |
| Armenia | 4046.77 (3028.82 to 5329.02) | | 5121.94 (3639.68 to 7025.7) | 0.89 (0.77 to 1.01) |
| Australia | 7791.5 (6192.07 to 9651.28) | | 8716.12 (6286.32 to 11906.4) | 0.43 (0.35 to 0.52) |
| Austria | 6285.7 (4767.85 to 8148.48) | | 6358.81 (4527.09 to 8813.61) | 0.11 (-0.05 to 0.27) |
| Azerbaijan | 4036.1 (3029.66 to 5331.07) | | 4936.89 (3508.75 to 6702.87) | 0.71 (0.65 to 0.76) |
| Bahamas | 4648.31 (3365.79 to 6328.94) | | 5811.59 (3979.1 to 8392.37) | 0.87 (0.7 to 1.05) |
| Bahrain | 8410.9 (6115.19 to 11345.09) | | 8908.9 (6171.2 to 12654.85) | 0.25 (0.18 to 0.32) |
| Bangladesh | 7697.68 (5733.9 to 10271.28) | | 8358.88 (5873.79 to 11584.21) | 0.38 (0.32 to 0.45) |
| Barbados | 4613.06 (3325.78 to 6230.84) | | 5921.62 (4096.1 to 8496.49) | 0.81 (0.75 to 0.87) |
| Belarus | 5577.87 (4215.05 to 7324.49) | | 7778.96 (5494.84 to 10748.15) | 1.09 (1.06 to 1.13) |
| Belgium | 5663.05 (4763.32 to 6750.3) | | 7323.57 (5213.02 to 10119.69) | 0.98 (0.49 to 1.47) |
| Belize | 4699.72 (3428.77 to 6406.75) | | 5750.78 (4000.53 to 8113.72) | 0.84 (0.66 to 1.02) |
| Benin | 6242.35 (4681.58 to 8315.2) | | 6679.72 (4723.81 to 9185.65) | 0.26 (0.21 to 0.3) |
| Bermuda | 5419.36 (4001.2 to 7334.36) | | 5853.94 (3977.25 to 8303.55) | 0.29 (0.25 to 0.33) |
| Bhutan | 6043.72 (4506.91 to 7999.12) | | 5671.33 (4036.64 to 7737.32) | -0.15 (-0.25 to -0.06) |
| Bolivia (Plurinational State of) | 5634.17 (4172.42 to 7586.39) | | 7081.79 (4907.28 to 10012.75) | 0.99 (0.86 to 1.12) |
| Bosnia and Herzegovina | 5181.06 (3906.61 to 6834.62) | | 5407.21 (3804.32 to 7547.03) | 0.19 (0.12 to 0.26) |
| Botswana | 6172.09 (4572.13 to 8123.19) | | 7665.49 (5420.01 to 10613.12) | 0.64 (0.56 to 0.73) |
| Brazil | 6702.03 (5315.13 to 8376.01) | | 7641.53 (5955.81 to 9640.5) | 0.61 (0.24 to 0.97) |
| Brunei Darussalam | 2776.01 (2047.61 to 3716.01) | | 3155.59 (2225.67 to 4444.72) | 0.45 (0.39 to 0.51) |
| Bulgaria | 4534.24 (3455.69 to 5897.07) | | 5548.22 (3972.58 to 7631.19) | 0.66 (0.61 to 0.7) |
| Burkina Faso | 6067.23 (4568.33 to 8017.89) | | 6163.96 (4461.83 to 8494.81) | 0.03 (-0.26 to 0.33) |
| Burundi | 7765.25 (5749.57 to 10234.09) | | 7498.07 (5367.72 to 10333.36) | -0.08 (-0.12 to -0.03) |
| Cabo Verde | 6087.41 (4541.52 to 8070.1) | | 7559.99 (5257.1 to 10573.62) | 0.77 (0.61 to 0.92) |
| Cambodia | 4714.04 (3537.5 to 6241.97) | | 4749.02 (3444.96 to 6340.18) | -0.01 (-0.06 to 0.04) |
| Cameroon | 6474.24 (4837.29 to 8612.99) | | 6911.45 (4951.74 to 9536.96) | 0.23 (0.21 to 0.25) |
| Canada | 6193.06 (4895.3 to 7724.02) | | 7941.77 (5673.3 to 10818.18) | 0.89 (0.81 to 0.98) |
| Central African Republic | 9340.04 (6828.01 to 12650.73) | | 9760.41 (6770.57 to 13773.02) | 0.21 (-0.03 to 0.44) |
| Chad | 7317.49 (5421.07 to 9701.62) | | 7618.46 (5360.17 to 10588.78) | 0.22 (0.06 to 0.38) |
| Chile | 8208.27 (6700.05 to 10044.34) | | 8851.05 (6015.54 to 12584.37) | 0.41 (0.3 to 0.52) |
| China | 4724.65 (3835.87 to 5756.45) | | 3695.55 (2997.95 to 4502.63) | -0.8 (-0.94 to -0.66) |
| Colombia | 3396 (2489.58 to 4533.77) | | 3221.5 (2316.72 to 4351.35) | -0.1 (-0.52 to 0.31) |
| Comoros | 6166.41 (4633.37 to 8092.71) | | 6712.16 (4820.32 to 9217.44) | 0.32 (0.27 to 0.37) |
| Congo | 9332.96 (6819.95 to 12565.17) | | 9901.89 (6909.19 to 14048.29) | 0.24 (-0.05 to 0.53) |
| Cook Islands | 4421.24 (3213.12 to 6079.56) | | 4829.23 (3382.31 to 6852.3) | 0.27 (0.22 to 0.32) |
| Costa Rica | 4580.57 (3320.17 to 6272.15) | | 5853.77 (4024.79 to 8431.54) | 0.91 (0.8 to 1.02) |
| Coted'Ivoire | 5666.46 (4259.8 to 7468.69) | | 5818.12 (4207.91 to 7870.6) | 0.06 (0.01 to 0.11) |
| Croatia | 5097.65 (3871.23 to 6589.63) | | 5091.08 (3663.41 to 6921.08) | 0.06 (0.02 to 0.1) |
| Cuba | 8104.51 (6125.94 to 10408.93) | | 6489.07 (4551.95 to 9194.29) | -0.73 (-0.84 to -0.62) |
| Cyprus | 5651.6 (4108.64 to 7645.1) | | 6564.33 (4557.58 to 9240.27) | 0.53 (0.46 to 0.59) |
| Czechia | 5025.83 (3856.61 to 6527.53) | | 5283.08 (3746.89 to 7221.84) | 0.2 (0.15 to 0.25) |
| Democratic People's Republic of Korea | 4032.36 (3045.06 to 5281.03) | | 3860.68 (2843.12 to 5159.24) | -0.14 (-0.15 to -0.13) |
| Democratic Republic of the Congo | 8817.25 (6517.12 to 11732.38) | | 9030.64 (6365.71 to 12452.1) | 0.18 (0 to 0.36) |
| Denmark | 7940.24 (6004.47 to 10378.62) | | 7208.71 (5079.54 to 10014.49) | -0.26 (-0.31 to -0.21) |
| Djibouti | 6380.83 (4737.52 to 8529.92) | | 7035.72 (5080.09 to 9588.88) | 0.35 (0.24 to 0.46) |
| Dominica | 4628.86 (3324.74 to 6368.69) | | 5728.76 (3931.79 to 8219.2) | 0.73 (0.68 to 0.77) |
| Dominican Republic | 5789.03 (4162.6 to 7952.77) | | 6654.06 (4482.49 to 9610.36) | 0.61 (0.25 to 0.96) |
| Ecuador | 5042.96 (3709.29 to 6701.42) | | 6352.78 (4417.6 to 8814.19) | 0.98 (0.9 to 1.07) |
| Egypt | 6400.57 (4615.66 to 8776.5) | | 7572.92 (5215.41 to 10767.38) | 0.6 (0.5 to 0.71) |
| El Salvador | 6240.84 (4582.15 to 8369.01) | | 6439.69 (4518.43 to 9026.33) | 0.16 (-0.14 to 0.46) |
| Equatorial Guinea | 9461.27 (6958.47 to 12840.23) | | 9912.19 (6898.55 to 13934.88) | 0.22 (0.04 to 0.41) |
| Eritrea | 6970.63 (5218.42 to 9192) | | 7272.31 (5240.24 to 9943.64) | 0.14 (0.13 to 0.15) |
| Estonia | 6844.37 (5186.94 to 8852.13) | | 6615.42 (4696.72 to 9277.4) | -0.14 (-0.21 to -0.08) |
| Eswatini | 6121.72 (4567.95 to 8071.72) | | 8757.22 (6163.63 to 12204.74) | 1.15 (1.06 to 1.24) |
| Ethiopia | 6870.39 (5436.31 to 8641.01) | | 6820.36 (5345.69 to 8728.26) | -0.02 (-0.14 to 0.1) |
| Fiji | 4112.11 (3103.6 to 5366.98) | | 4632.31 (3382.45 to 6256.51) | 0.36 (0.34 to 0.37) |
| Finland | 8707.44 (6584.75 to 11219.88) | | 9212.66 (6578.93 to 12522.56) | 0.27 (-0.18 to 0.72) |
| France | 7926.84 (6749.16 to 9273.48) | | 8290.03 (5877.04 to 11549.82) | 0.23 (-0.11 to 0.57) |
| Gabon | 8616.27 (6333.64 to 11704.2) | | 9434.54 (6589.3 to 13076.96) | 0.32 (0.22 to 0.41) |
| Gambia | 9685.39 (7133.19 to 13140.04) | | 10074.23 (6948.41 to 14192.82) | 0.18 (0.14 to 0.22) |
| Georgia | 4366.77 (3269.12 to 5794.01) | | 5382.96 (3780.63 to 7474.98) | 0.68 (0.66 to 0.71) |
| Germany | 5195.82 (4205.49 to 6427.45) | | 6976.31 (4886.2 to 9769.63) | 0.97 (0.75 to 1.2) |
| Ghana | 6241.52 (4625.72 to 8243) | | 6650.32 (4702 to 9257.98) | 0.24 (0.13 to 0.36) |
| Greece | 8687.44 (6132.18 to 12095.36) | | 10839 (7231.31 to 15717.3) | 0.59 (-0.11 to 1.3) |
| Greenland | 13879.12 (10446.14 to 18267.87) | | 15623.41 (11143.73 to 21322.5) | 0.48 (0.36 to 0.59) |
| Grenada | 4705.98 (3413.9 to 6434.89) | | 5719.57 (3944.64 to 8043.12) | 0.66 (0.62 to 0.69) |
| Guam | 4190.19 (3155.91 to 5473.5) | | 4779.61 (3539.59 to 6475.18) | 0.47 (0.42 to 0.52) |
| Guatemala | 5624.88 (4093.61 to 7757.94) | | 6760.51 (4587.59 to 9864.61) | 0.66 (0.36 to 0.95) |
| Guinea | 6362.05 (4757.1 to 8436.84) | | 6965.24 (4991.99 to 9575.81) | 0.31 (0.22 to 0.4) |
| Guinea-Bissau | 6516.84 (4885.46 to 8628.18) | | 7102.2 (5076.76 to 9768.35) | 0.31 (0.19 to 0.43) |
| Guyana | 7016.57 (5142.12 to 9320.16) | | 9955.92 (6789.44 to 14124.61) | 1.17 (1.12 to 1.22) |
| Haiti | 5809.79 (4222.6 to 7986.28) | | 6571.49 (4534.8 to 9263.48) | 0.44 (0.35 to 0.53) |
| Honduras | 4492.03 (3261.49 to 6099.22) | | 5847.55 (3945.2 to 8426.18) | 1 (0.91 to 1.08) |
| Hungary | 5199.55 (3998.77 to 6687.35) | | 4858.84 (3531.86 to 6601.04) | -0.22 (-0.24 to -0.19) |
| Iceland | 5892.28 (4427.92 to 7715.35) | | 5775.34 (4108.17 to 7922.58) | -0.06 (-0.25 to 0.14) |
| India | 6094.34 (4842.23 to 7573.36) | | 6089.46 (4834.93 to 7583.28) | 0.08 (-0.05 to 0.2) |
| Indonesia | 3576.57 (2846.85 to 4412.35) | | 4171.85 (3305.93 to 5211.81) | 0.51 (0.48 to 0.55) |
| Iran (Islamic Republic of) | 8687.9 (6609.36 to 11421.04) | | 9946.24 (7478.66 to 13076.52) | 0.6 (0.38 to 0.82) |
| Iraq | 6821.52 (5080.75 to 9021.33) | | 7296.48 (5148.34 to 10031.24) | 0.23 (-0.66 to 1.13) |
| Ireland | 6832.45 (5305.29 to 8776.64) | | 9232.8 (6425.69 to 12915.92) | 1.1 (0.88 to 1.32) |
| Israel | 7464.54 (5521.41 to 10066.31) | | 8291.44 (5709.1 to 11701.82) | 0.42 (0.34 to 0.51) |
| Italy | 6614.35 (5081.03 to 8546.8) | | 8119.67 (6171.39 to 10628.79) | 0.8 (0.71 to 0.89) |
| Jamaica | 4671.98 (3348.89 to 6391.81) | | 5842.13 (3952.44 to 8479.08) | 0.73 (0.69 to 0.77) |
| Japan | 3468.73 (2827.1 to 4217.42) | | 4470.53 (3597.43 to 5528.13) | 0.86 (0.64 to 1.08) |
| Jordan | 7843.86 (5686.08 to 10697.4) | | 8009.33 (5516.44 to 11383.63) | 0.1 (0.04 to 0.17) |
| Kazakhstan | 4771.17 (3635.65 to 6190.99) | | 5622.33 (4020.04 to 7619.8) | 0.57 (0.32 to 0.81) |
| Kenya | 6953.88 (5566.11 to 8609.85) | | 7078.98 (5616.55 to 8791.86) | 0.11 (-0.07 to 0.29) |
| Kiribati | 4222.57 (3184.24 to 5514.52) | | 4227.31 (3106.89 to 5661.27) | -0.01 (-0.04 to 0.02) |
| Kuwait | 6984 (5081.74 to 9490.11) | | 6941.63 (4802.15 to 9923.38) | 0.06 (-0.14 to 0.27) |
| Kyrgyzstan | 4940.59 (3735.47 to 6425.39) | | 5720.35 (4166.74 to 7747.17) | 0.59 (0.49 to 0.68) |
| Lao People's Democratic Republic | 4025.18 (3029.25 to 5310.1) | | 3949.06 (2833.5 to 5309.72) | -0.1 (-0.46 to 0.27) |
| Latvia | 5993 (4542.69 to 7784.78) | | 6786.38 (4695.81 to 9508.96) | 0.32 (0.15 to 0.49) |
| Lebanon | 6940.77 (5231.96 to 9161.73) | | 10271.29 (6980.06 to 14684.74) | 1.24 (1.13 to 1.36) |
| Lesotho | 8911.22 (6561.44 to 11927.34) | | 11151.88 (7761.43 to 15442.99) | 0.7 (0.66 to 0.74) |
| Liberia | 7674.22 (5678.95 to 10214.06) | | 7922.18 (5596.75 to 10983.49) | 0.17 (0.14 to 0.2) |
| Libya | 7172.3 (5188.27 to 9769.09) | | 7971.99 (5483.81 to 11190.4) | 0.41 (0.21 to 0.61) |
| Lithuania | 6167.58 (4691.38 to 7988.77) | | 7787.36 (5552.11 to 10697.02) | 0.75 (0.71 to 0.79) |
| Luxembourg | 6885.99 (5199.92 to 8912.08) | | 6519.65 (4705.45 to 8736.3) | -0.1 (-0.29 to 0.08) |
| Madagascar | 6794.29 (5108.18 to 8896.58) | | 7337.4 (5206.42 to 10019.73) | 0.3 (0.23 to 0.37) |
| Malawi | 6259.73 (4716.13 to 8219.04) | | 6629.25 (4796.02 to 9092.15) | 0.18 (0.16 to 0.2) |
| Malaysia | 4336.82 (3262.1 to 5732.85) | | 4926.06 (3524.42 to 6801.28) | 0.28 (-0.23 to 0.79) |
| Maldives | 5700.83 (4319.36 to 7448.84) | | 5022.42 (3679.51 to 6787.41) | -0.39 (-0.44 to -0.34) |
| Mali | 5469.8 (4097.45 to 7160.73) | | 5556.05 (4028.46 to 7549.73) | 0.1 (0.05 to 0.14) |
| Malta | 5655 (4105.5 to 7569.99) | | 6460.13 (4417.9 to 9093.35) | 0.58 (0.38 to 0.78) |
| Marshall Islands | 4068.15 (3052.7 to 5347.1) | | 4276.88 (3151.72 to 5790) | 0.15 (0.11 to 0.18) |
| Mauritania | 5184.76 (3917.54 to 6753.6) | | 5369.97 (3848.09 to 7274.17) | 0.15 (0.09 to 0.21) |
| Mauritius | 6844.27 (5119.4 to 9000.83) | | 7219.25 (5050.8 to 10015.54) | 0.24 (0.04 to 0.44) |
| Mexico | 4637.34 (3646.08 to 5827.81) | | 7715.07 (5925.12 to 9853.58) | 1.86 (1.74 to 1.99) |
| Micronesia (Federated States of) | 4154.85 (3124.09 to 5481.64) | | 4247.19 (3079 to 5684.35) | 0.05 (0.02 to 0.09) |
| Monaco | 7105.33 (4948.62 to 10173.53) | | 8801.84 (5812.19 to 13023.26) | 0.75 (0.72 to 0.78) |
| Mongolia | 5909.26 (4418.33 to 7840.87) | | 5876.91 (4292.91 to 7984.16) | -0.05 (-0.15 to 0.04) |
| Montenegro | 4468.7 (3395.44 to 5778.55) | | 5594.84 (4009.04 to 7678.43) | 0.78 (0.74 to 0.82) |
| Morocco | 8280.93 (6022.98 to 11130.09) | | 9413.66 (6509.44 to 13375.75) | 0.49 (0.19 to 0.79) |
| Mozambique | 6672.59 (4998.43 to 8870.43) | | 7694.96 (5457.42 to 10741.32) | 0.45 (0.43 to 0.47) |
| Myanmar | 3038.99 (2227.21 to 4071.1) | | 3547.4 (2569.11 to 4866.6) | 0.48 (0.35 to 0.62) |
| Namibia | 5392.35 (4072.58 to 6989.77) | | 6751.54 (4851.62 to 9290.51) | 0.66 (0.42 to 0.91) |
| Nauru | 4396.55 (3180.91 to 5999.79) | | 4816.41 (3388.03 to 6775.79) | 0.27 (0.22 to 0.33) |
| Nepal | 6319.78 (4740.51 to 8288.59) | | 8382.29 (5941.69 to 11747.11) | 0.96 (0.55 to 1.38) |
| Netherlands | 6497.93 (5494.51 to 7707.65) | | 7570.26 (5307 to 10543.85) | 0.65 (0.42 to 0.89) |
| New Zealand | 6649.54 (5082.32 to 8670.44) | | 7170.46 (5454.64 to 9445.48) | 0.22 (0.1 to 0.33) |
| Nicaragua | 5020.86 (3664.99 to 6768.68) | | 6002.92 (4189.99 to 8436.37) | 0.72 (0.59 to 0.84) |
| Niger | 6280.96 (4669.63 to 8357.49) | | 6210.13 (4455.68 to 8373.37) | -0.05 (-0.09 to -0.01) |
| Nigeria | 6013.29 (4723.45 to 7601.28) | | 5370.11 (4240.97 to 6735.03) | -0.31 (-0.49 to -0.14) |
| Niue | 4402.8 (3205.54 to 6006.57) | | 4811.17 (3409.1 to 6742.24) | 0.27 (0.21 to 0.32) |
| North Macedonia | 4212.1 (3174.62 to 5495.47) | | 5268.12 (3788.47 to 7298.18) | 0.78 (0.7 to 0.87) |
| Northern Mariana Islands | 3630.19 (2692.02 to 4829.83) | | 4251.56 (3083.98 to 5793.78) | 0.51 (0.46 to 0.55) |
| Norway | 6318.72 (4950.06 to 8053.16) | | 7608.04 (5756.43 to 9921.45) | 0.57 (0.28 to 0.86) |
| Oman | 6735.03 (4845.95 to 9216.07) | | 7833.77 (5325.34 to 11218.86) | 0.61 (0.55 to 0.66) |
| Pakistan | 6100.59 (4704.38 to 7811.43) | | 6517.31 (4935.7 to 8469.33) | 0.36 (0.1 to 0.62) |
| Palau | 4407.96 (3204.87 to 6030.75) | | 4826.81 (3409.88 to 6899.34) | 0.27 (0.22 to 0.32) |
| Palestine | 9100.7 (6491.1 to 12584.52) | | 10277.83 (6930.18 to 14929.98) | 0.48 (0.37 to 0.6) |
| Panama | 4392.73 (3183.57 to 5981.72) | | 5395.97 (3681.27 to 7822.94) | 0.89 (0.82 to 0.97) |
| Papua New Guinea | 4518.51 (3378.97 to 5973.14) | | 4495.91 (3290.56 to 6042.97) | -0.03 (-0.08 to 0.02) |
| Paraguay | 5429.79 (3976.46 to 7357.19) | | 7246.51 (4996.63 to 10266.51) | 0.97 (0.94 to 1) |
| Peru | 3623.59 (2726.33 to 4777.3) | | 4326.45 (3086.66 to 5972.09) | 0.69 (0.23 to 1.15) |
| Philippines | 4292.3 (3402.25 to 5336.24) | | 4717.28 (3719.95 to 5923.79) | 0.36 (0.3 to 0.43) |
| Poland | 3309.44 (2637.74 to 4118.42) | | 3768.22 (2979.24 to 4743.7) | 0.46 (0.41 to 0.51) |
| Portugal | 9233.97 (6735.82 to 12244.32) | | 10290.15 (7057.91 to 14864.56) | 0.41 (-0.34 to 1.17) |
| Puerto Rico | 3937.23 (2949.59 to 5173.6) | | 4597.24 (3237.36 to 6479.5) | 0.57 (0.32 to 0.81) |
| Qatar | 7654.96 (5588.14 to 10243.05) | | 7725.64 (5302.87 to 11057.09) | 0.08 (-0.13 to 0.28) |
| Republic of Korea | 3461.74 (2772.86 to 4331.01) | | 4026.54 (2964.06 to 5347.61) | 0.56 (0.46 to 0.66) |
| Republic of Moldova | 5299.15 (4023.06 to 6882.72) | | 5658.67 (4028.6 to 7918.53) | 0.28 (0.2 to 0.35) |
| Romania | 4122.32 (3108.37 to 5365.33) | | 5007.59 (3576.2 to 6893.41) | 0.7 (0.63 to 0.77) |
| Russian Federation | 4627.58 (3629.68 to 5837.66) | | 5707.72 (4430.71 to 7299.01) | 0.69 (0.37 to 1.02) |
| Rwanda | 8455.27 (6258.64 to 11311.39) | | 8388.95 (5935.54 to 11523.39) | -0.04 (-0.12 to 0.04) |
| Saint Kitts and Nevis | 5793.83 (4053.69 to 8305.12) | | 6928.16 (4511.5 to 10486.86) | 0.65 (0.59 to 0.72) |
| Saint Lucia | 4722.17 (3429.43 to 6454.14) | | 6155.71 (4205 to 8787.37) | 0.86 (0.8 to 0.91) |
| Saint Vincent and the Grenadines | 4702.28 (3403.75 to 6408.06) | | 5864.13 (4051.94 to 8360.45) | 0.74 (0.7 to 0.77) |
| Samoa | 4029.77 (3031.11 to 5291.7) | | 4162.41 (3033 to 5632.38) | 0.08 (0.04 to 0.12) |
| San Marino | 7139.52 (4988.51 to 10259.24) | | 8946.73 (5864.98 to 13464.18) | 0.92 (0.88 to 0.96) |
| Sao Tome and Principe | 5714.05 (4262.85 to 7553.91) | | 6075.64 (4346.15 to 8380.46) | 0.24 (0.14 to 0.33) |
| Saudi Arabia | 6810.69 (4864.3 to 9359.5) | | 7648.16 (5349.08 to 10796.64) | 0.57 (0.27 to 0.86) |
| Senegal | 5486.23 (4128.3 to 7245.08) | | 6228.02 (4523.47 to 8537.94) | 0.46 (0.35 to 0.58) |
| Serbia | 4705.65 (3594.91 to 6122.06) | | 5038.12 (3671.39 to 6845.51) | 0.27 (0.21 to 0.32) |
| Seychelles | 3674.58 (2725.73 to 4878) | | 4287.93 (3067.42 to 5926.56) | 0.49 (0.44 to 0.54) |
| Sierra Leone | 6179.68 (4614.61 to 8149.14) | | 6629.06 (4764.6 to 9098.54) | 0.26 (0.21 to 0.31) |
| Singapore | 5203.52 (4232.63 to 6438.46) | | 3606.56 (2616.5 to 4926.4) | -1.14 (-1.29 to -1) |
| Slovakia | 4441.6 (3374.08 to 5771.1) | | 5302.25 (3755.64 to 7385.86) | 0.53 (0.35 to 0.7) |
| Slovenia | 5893.26 (4535.72 to 7593.75) | | 5689.24 (4103.92 to 7863.84) | -0.04 (-0.16 to 0.08) |
| Solomon Islands | 4401.39 (3297.39 to 5818.95) | | 4537.54 (3288.4 to 6163.01) | 0.09 (0.04 to 0.14) |
| Somalia | 7080.69 (5296.96 to 9316.49) | | 8315.82 (5955.29 to 11565.86) | 0.53 (0.47 to 0.58) |
| South Africa | 6732.72 (5435.78 to 8220.37) | | 8172.99 (6482.16 to 10191.87) | 0.78 (0.51 to 1.06) |
| South Sudan | 6845.59 (5136.28 to 8961.27) | | 7251.93 (5204.28 to 9963.97) | 0.25 (0.13 to 0.37) |
| Spain | 7141.16 (6027.33 to 8454.34) | | 10080.65 (7191.22 to 13906.82) | 1.35 (1.04 to 1.67) |
| Sri Lanka | 5086.27 (3904.56 to 6484.13) | | 4880.87 (3620.89 to 6510.98) | -0.16 (-0.28 to -0.04) |
| Sudan | 7146.75 (5220.06 to 9665.18) | | 7666.66 (5352.25 to 10923.16) | 0.22 (-0.02 to 0.46) |
| Suriname | 6880.8 (5081.41 to 9217.2) | | 9634.94 (6677.4 to 13576.8) | 1.09 (1 to 1.18) |
| Sweden | 8325.68 (6611.75 to 10327.75) | | 9788.27 (7402.5 to 12801.83) | 0.53 (0.22 to 0.85) |
| Switzerland | 7868.4 (6025.62 to 10087.87) | | 7790.18 (5538.6 to 10719.25) | -0.02 (-0.12 to 0.08) |
| Syrian Arab Republic | 6707.95 (4813.56 to 9332.52) | | 7698.29 (5258.78 to 11019.25) | 0.53 (0.44 to 0.62) |
| Taiwan (Province of China) | 3367.15 (2521.06 to 4454.95) | | 3733.87 (2745.59 to 4964.46) | 0.34 (0.31 to 0.36) |
| Tajikistan | 4396.39 (3297.78 to 5779.88) | | 4963.11 (3555.74 to 6810.47) | 0.41 (0.35 to 0.47) |
| Thailand | 4140.21 (3133.53 to 5398.56) | | 4331.09 (3136.61 to 5814.41) | 0.13 (0.11 to 0.15) |
| Timor-Leste | 4409.52 (3291.9 to 5828.59) | | 4372.59 (3153.07 to 5934.71) | -0.05 (-0.09 to -0.01) |
| Togo | 6322.23 (4733.42 to 8291.83) | | 6578.96 (4703.07 to 8915.26) | 0.16 (0.05 to 0.27) |
| Tokelau | 4407.47 (3205.68 to 6053.07) | | 4810.25 (3374.56 to 6783.44) | 0.27 (0.22 to 0.31) |
| Tonga | 3925.36 (2949.2 to 5133.14) | | 4107.9 (3016.77 to 5543.87) | 0.13 (0.1 to 0.16) |
| Trinidad and Tobago | 6156.31 (4570.54 to 8206.78) | | 7588.87 (5193.26 to 10928.96) | 0.62 (0.53 to 0.71) |
| Tunisia | 7966.21 (5710.86 to 10839.64) | | 10137.08 (6805.74 to 14762.34) | 0.79 (0.72 to 0.85) |
| Turkey | 6970.57 (5676.27 to 8509.34) | | 8134.31 (5530.83 to 11763.83) | 0.57 (0.33 to 0.8) |
| Turkmenistan | 4595.53 (3477.08 to 5991.68) | | 5060.38 (3651.09 to 6976.25) | 0.37 (0.33 to 0.4) |
| Tuvalu | 4388.53 (3205.45 to 6035) | | 4779.75 (3357.58 to 6663.9) | 0.26 (0.22 to 0.29) |
| Uganda | 9321.19 (6889.08 to 12408.22) | | 9981.02 (7023.93 to 14031.7) | 0.23 (0.09 to 0.37) |
| Ukraine | 6006.83 (4615.79 to 7780.39) | | 7034.41 (5116.13 to 9520.52) | 0.48 (0.31 to 0.66) |
| United Arab Emirates | 6837.54 (5048.56 to 9208.2) | | 7275.73 (5046.09 to 10312.98) | 0.29 (0.25 to 0.32) |
| United Kingdom | 7770.81 (6002.83 to 10046.36) | | 8440.8 (6445.34 to 10953.38) | 0.5 (0.17 to 0.84) |
| United Republic of Tanzania | 6814.34 (5131.83 to 8989.1) | | 7220.83 (5204.08 to 9811.39) | 0.21 (0.19 to 0.23) |
| United States of America | 7095.39 (5775.34 to 8638.87) | | 10709.46 (8679.46 to 13101.25) | 1.44 (1.27 to 1.61) |
| United States Virgin Islands | 4758.19 (3487.77 to 6458.62) | | 5768.45 (4052.16 to 8138.47) | 0.67 (0.56 to 0.78) |
| Uruguay | 4990.91 (3668.8 to 6729.79) | | 7051.84 (4892.92 to 9902.71) | 1.11 (0.77 to 1.44) |
| Uzbekistan | 4709.36 (3575.42 to 6127.57) | | 5055.67 (3614.66 to 6908.7) | 0.28 (0.18 to 0.39) |
| Vanuatu | 4352.89 (3269.17 to 5749.01) | | 4485.32 (3255.22 to 6103.53) | 0.08 (0.03 to 0.13) |
| Venezuela (Bolivarian Republic of) | 4625.77 (3388.4 to 6277.05) | | 5081.86 (3503.33 to 7236.73) | 0.3 (0.17 to 0.43) |
| Viet Nam | 3966.03 (2997.7 to 5212.13) | | 4154.23 (3032.14 to 5565.92) | 0.14 (0.02 to 0.26) |
| Yemen | 8471.75 (6141.85 to 11477.68) | | 8425.26 (5765.38 to 11941.14) | -0.13 (-0.18 to -0.08) |
| Zambia | 5958.26 (4503.02 to 7831.82) | | 6563.27 (4744.54 to 8880.47) | 0.28 (0.23 to 0.33) |
| Zimbabwe | 4675.85 (3472.13 to 6133.84) | | 5310.4 (3841.37 to 7203.78) | 0.4 (0.26 to 0.53) |

Supplementary Table 3. Age-standardized DALYs rate of depressive disorders in 1990 and 2021 for women of childbearing age in 204 countries, with AAPCs from 1990 and 2021. Abbreviations: ASDR, age-standardized DALYs rate; UI, uncertainty interval; AAPC, average annual percent change; CI, confidence interval.

| location | ASDR, per 100,000 (95% UI) | | AAPC (95% CI) |
| --- | --- | --- | --- |
|  | 1990 | 2021 |  |
| Afghanistan | 1467.19 (872 to 2288.33) | 1738.49 (1006.75 to 2735.55) | 0.62 (0.58 to 0.67) |
| Albania | 629.52 (383.85 to 953.55) | 857.6 (508.91 to 1375.79) | 1.12 (1.01 to 1.23) |
| Algeria | 1251.56 (733.49 to 1957.49) | 1362.78 (756.91 to 2220.47) | 0.39 (0.16 to 0.61) |
| American Samoa | 563.6 (342.18 to 846.96) | 615.2 (365.8 to 948.5) | 0.26 (0.18 to 0.33) |
| Andorra | 1177.68 (703.29 to 1798.15) | 1437.56 (815.97 to 2304.14) | 0.81 (0.45 to 1.18) |
| Angola | 1699.88 (1018.64 to 2642.69) | 1864.14 (1045.86 to 2993.97) | 0.37 (0.25 to 0.49) |
| Antigua and Barbuda | 811.61 (472.89 to 1268.19) | 1021.56 (578.3 to 1691.03) | 0.8 (0.76 to 0.84) |
| Argentina | 846.26 (515.48 to 1294.36) | 1024.7 (616.35 to 1540.41) | 0.77 (0.69 to 0.85) |
| Armenia | 657.37 (395.32 to 1000.41) | 883.79 (507.39 to 1416.46) | 1.12 (0.97 to 1.28) |
| Australia | 1422.72 (917.32 to 2070.04) | 1616.63 (973.34 to 2487.3) | 0.48 (0.41 to 0.56) |
| Austria | 1121.09 (690.69 to 1667.13) | 1134.93 (662.58 to 1779.43) | 0.12 (-0.07 to 0.31) |
| Azerbaijan | 654.65 (391.23 to 1001.4) | 843.98 (478.28 to 1354.82) | 0.89 (0.81 to 0.97) |
| Bahamas | 817.06 (478.2 to 1258.82) | 1057.73 (596.04 to 1720.37) | 1 (0.8 to 1.19) |
| Bahrain | 1539.52 (934.76 to 2361.57) | 1639.21 (947.15 to 2608.56) | 0.27 (0.2 to 0.35) |
| Bangladesh | 1391.32 (839.25 to 2093.55) | 1532.24 (868.79 to 2434.64) | 0.44 (0.37 to 0.52) |
| Barbados | 812.42 (476.49 to 1270.26) | 1081.02 (605.98 to 1761.89) | 0.93 (0.86 to 1.01) |
| Belarus | 974.97 (595.94 to 1469.08) | 1431.07 (835.54 to 2214.49) | 1.27 (1.23 to 1.31) |
| Belgium | 1009.85 (684.18 to 1407.79) | 1348.66 (779.7 to 2084.89) | 1.14 (0.53 to 1.75) |
| Belize | 828.47 (492.22 to 1295.77) | 1044.23 (600.22 to 1655.07) | 0.87 (0.59 to 1.15) |
| Benin | 1045.08 (633.76 to 1571.76) | 1142.83 (675.38 to 1814.83) | 0.33 (0.29 to 0.37) |
| Bermuda | 983.4 (589.81 to 1515.3) | 1074.18 (595.5 to 1751.46) | 0.33 (0.29 to 0.37) |
| Bhutan | 1050.61 (631.11 to 1595.83) | 979.32 (563.36 to 1572.56) | -0.16 (-0.28 to -0.04) |
| Bolivia (Plurinational State of) | 1019.53 (612.55 to 1579.54) | 1318.08 (748.8 to 2095.14) | 0.97 (0.44 to 1.49) |
| Bosnia and Herzegovina | 895.74 (545.55 to 1346.58) | 940.57 (538.15 to 1517.89) | 0.23 (0.15 to 0.31) |
| Botswana | 1039.27 (635.89 to 1567.97) | 1333.69 (773.21 to 2042.27) | 0.75 (0.66 to 0.85) |
| Brazil | 1231.4 (799.38 to 1789.95) | 1420.96 (899.62 to 2073.59) | 0.65 (0.25 to 1.06) |
| Brunei Darussalam | 470.92 (281.98 to 733.24) | 550.2 (307.92 to 886.51) | 0.55 (0.48 to 0.61) |
| Bulgaria | 758.91 (467.95 to 1135.85) | 969.02 (564.4 to 1524.96) | 0.8 (0.75 to 0.86) |
| Burkina Faso | 1013.42 (619.8 to 1525.61) | 1039.72 (612.19 to 1631.59) | 0.07 (-0.37 to 0.52) |
| Burundi | 1339.96 (814.25 to 2019.6) | 1287.92 (741.1 to 2039.59) | -0.08 (-0.13 to -0.04) |
| Cabo Verde | 1028.45 (612.48 to 1568.92) | 1336.91 (775.81 to 2161.69) | 0.93 (0.75 to 1.1) |
| Cambodia | 763.07 (464.57 to 1162.5) | 776.41 (466.92 to 1211.89) | 0.01 (-0.06 to 0.08) |
| Cameroon | 1095.17 (667.7 to 1674) | 1190.77 (698.59 to 1857.28) | 0.28 (0.21 to 0.35) |
| Canada | 1065.83 (691.49 to 1550.91) | 1428.52 (854.37 to 2219.08) | 1.05 (0.96 to 1.13) |
| Central African Republic | 1674.02 (1007.82 to 2593.02) | 1769.99 (1038.09 to 2776.21) | 0.04 (-0.05 to 0.14) |
| Chad | 1272.41 (766.24 to 1940.55) | 1337.68 (782.91 to 2132.62) | 0.27 (0.08 to 0.47) |
| Chile | 1572.71 (1043.02 to 2249.21) | 1701.85 (965.04 to 2644.47) | 0.43 (0.31 to 0.54) |
| China | 773.43 (505.65 to 1117.2) | 559.4 (365.96 to 798.15) | -0.99 (-1.3 to -0.68) |
| Colombia | 582.78 (350.21 to 901.87) | 547.77 (316.78 to 861.78) | -0.12 (-0.54 to 0.31) |
| Comoros | 1011.33 (619.12 to 1526.9) | 1129.22 (646.41 to 1814.21) | 0.42 (0.36 to 0.48) |
| Congo | 1681.48 (1018.17 to 2598.25) | 1805.21 (1035.47 to 2895.4) | 0.29 (-0.06 to 0.63) |
| Cook Islands | 709.18 (424.01 to 1121.37) | 793.46 (455.68 to 1295.49) | 0.34 (0.28 to 0.4) |
| Costa Rica | 823.22 (491.17 to 1284.25) | 1084.8 (615.86 to 1761.8) | 1.02 (0.91 to 1.14) |
| Coted'Ivoire | 921.39 (561.26 to 1388.74) | 964.1 (567.59 to 1484.87) | 0.12 (0.06 to 0.19) |
| Croatia | 878.31 (539.66 to 1306.08) | 876.05 (522.28 to 1367.84) | 0.06 (0.02 to 0.1) |
| Cuba | 1534.42 (957.34 to 2325.6) | 1199.67 (714.01 to 1926.6) | -0.81 (-0.89 to -0.72) |
| Cyprus | 991.89 (581.75 to 1537.79) | 1180.53 (661.48 to 1886.54) | 0.62 (0.53 to 0.7) |
| Czechia | 859.96 (527.06 to 1273.36) | 915.75 (530.33 to 1426.95) | 0.24 (0.17 to 0.32) |
| Democratic People's Republic of Korea | 635.08 (391.56 to 954.41) | 598.54 (362.73 to 917.84) | -0.19 (-0.21 to -0.17) |
| Democratic Republic of the Congo | 1558.58 (960.4 to 2387.84) | 1619 (943.67 to 2520.76) | 0.26 (0.04 to 0.47) |
| Denmark | 1463.71 (906.25 to 2179.38) | 1309.14 (762.47 to 2039.56) | -0.3 (-0.35 to -0.24) |
| Djibouti | 1059.39 (629.87 to 1622.07) | 1196.74 (682.66 to 1878.92) | 0.44 (0.31 to 0.58) |
| Dominica | 812.35 (477.43 to 1269.04) | 1039.42 (579.63 to 1722.24) | 0.84 (0.78 to 0.9) |
| Dominican Republic | 1055.59 (618.97 to 1652.7) | 1232.75 (677.03 to 2026.13) | 0.7 (0.28 to 1.12) |
| Ecuador | 902.99 (537.95 to 1388.48) | 1173.73 (653.94 to 1865.42) | 0.98 (0.53 to 1.43) |
| Egypt | 1125 (667.63 to 1763.04) | 1369.8 (774.82 to 2203.67) | 0.76 (0.57 to 0.95) |
| El Salvador | 1164.36 (709.18 to 1813.91) | 1208.36 (691.37 to 1956.69) | 0.2 (-0.14 to 0.55) |
| Equatorial Guinea | 1698.5 (1018.79 to 2617.19) | 1805.21 (1052.66 to 2908.53) | 0.34 (0.13 to 0.55) |
| Eritrea | 1171.87 (710.22 to 1765.77) | 1246.45 (704.25 to 1933.11) | 0.2 (0.18 to 0.23) |
| Estonia | 1239.55 (764.46 to 1867.28) | 1191.72 (697.84 to 1885.33) | -0.17 (-0.24 to -0.11) |
| Eswatini | 1033.43 (627.29 to 1554.84) | 1553.33 (875.69 to 2481.72) | 1.3 (1.15 to 1.45) |
| Ethiopia | 1137.4 (728.61 to 1664.3) | 1148.5 (732.79 to 1712.3) | 0.04 (-0.1 to 0.18) |
| Fiji | 641.77 (396.82 to 971.49) | 750.91 (449.61 to 1191.12) | 0.47 (0.44 to 0.5) |
| Finland | 1606.86 (1001.22 to 2396.11) | 1708.73 (1024.58 to 2629.22) | 0.28 (-0.07 to 0.64) |
| France | 1459.28 (976.07 to 2010.51) | 1531.95 (905.21 to 2395.1) | 0.35 (-0.06 to 0.75) |
| Gabon | 1533.72 (904.24 to 2347.78) | 1704.85 (977.61 to 2716.02) | 0.38 (0.28 to 0.48) |
| Gambia | 1762.72 (1063.05 to 2718.53) | 1841.43 (1031.13 to 2981.19) | 0.2 (0.16 to 0.25) |
| Georgia | 726.46 (440.78 to 1112.51) | 936.64 (532.88 to 1474.95) | 0.83 (0.8 to 0.87) |
| Germany | 902.45 (592.66 to 1288.35) | 1274.94 (747.14 to 1997.45) | 1.14 (0.8 to 1.49) |
| Ghana | 1049.66 (628.56 to 1620.04) | 1139.9 (670.8 to 1859.62) | 0.31 (0.17 to 0.46) |
| Greece | 1623.44 (947.09 to 2553.56) | 2062.84 (1154.22 to 3385.78) | 0.65 (-0.13 to 1.44) |
| Greenland | 2649.15 (1661.73 to 3999.62) | 3015.73 (1803.63 to 4729.04) | 0.52 (0.4 to 0.65) |
| Grenada | 828.37 (490 to 1288.19) | 1037.84 (578.58 to 1689.75) | 0.76 (0.71 to 0.82) |
| Guam | 663.24 (410.03 to 996.9) | 785.89 (467.25 to 1229.99) | 0.61 (0.55 to 0.67) |
| Guatemala | 1021.94 (599.12 to 1598.76) | 1258.66 (708.61 to 2081.29) | 0.75 (0.41 to 1.09) |
| Guinea | 1074.62 (644.84 to 1631.12) | 1203.7 (703.87 to 1906.31) | 0.39 (0.28 to 0.5) |
| Guinea-Bissau | 1106.37 (658.64 to 1703.15) | 1228.17 (714.31 to 1941.24) | 0.39 (0.24 to 0.53) |
| Guyana | 1298.86 (788.41 to 1973.55) | 1895.95 (1108.9 to 3047.55) | 1.27 (1.21 to 1.33) |
| Haiti | 1041.64 (616.97 to 1618.25) | 1198.04 (679.13 to 1892.76) | 0.54 (0.42 to 0.66) |
| Honduras | 800.06 (471.89 to 1247.01) | 1077.42 (594.07 to 1748.12) | 1.13 (1.04 to 1.21) |
| Hungary | 894.94 (558.36 to 1335.05) | 827.54 (481.44 to 1304.46) | -0.25 (-0.29 to -0.21) |
| Iceland | 1041.24 (639.11 to 1568.62) | 1015.97 (584.09 to 1595.77) | -0.06 (-0.3 to 0.18) |
| India | 1054.95 (676.64 to 1547.89) | 1055.26 (677.78 to 1533.98) | 0.1 (-0.04 to 0.23) |
| Indonesia | 532.25 (344.5 to 770.85) | 658.51 (423.78 to 966.01) | 0.71 (0.66 to 0.76) |
| Iran (Islamic Republic of) | 1577.37 (978.55 to 2367.72) | 1834.46 (1140.72 to 2763.83) | 0.64 (0.37 to 0.92) |
| Iraq | 1205.45 (737.55 to 1828.13) | 1299.1 (763.76 to 2065.02) | 0.4 (-0.7 to 1.5) |
| Ireland | 1241.29 (789.85 to 1817.8) | 1732.99 (1018.68 to 2725.72) | 1.22 (1.04 to 1.39) |
| Israel | 1359.92 (822.57 to 2058.08) | 1531.79 (869.04 to 2497.5) | 0.48 (0.38 to 0.58) |
| Italy | 1190.02 (751.5 to 1764.83) | 1504.31 (939.71 to 2244.91) | 0.91 (0.8 to 1.02) |
| Jamaica | 824.1 (479.6 to 1295.03) | 1066.16 (607.18 to 1771.42) | 0.84 (0.78 to 0.9) |
| Japan | 612.69 (400.09 to 882.52) | 823.3 (534.89 to 1193.15) | 1.01 (0.79 to 1.23) |
| Jordan | 1422.61 (847.59 to 2214.94) | 1453.17 (823.97 to 2363.84) | 0.1 (0.04 to 0.17) |
| Kazakhstan | 806.73 (503.8 to 1201.82) | 986.53 (600.61 to 1546.09) | 0.69 (0.4 to 0.98) |
| Kenya | 1176.36 (756.14 to 1700.31) | 1201.6 (768.1 to 1750.34) | 0.14 (-0.08 to 0.36) |
| Kiribati | 660.78 (409.32 to 997.41) | 662.21 (401.28 to 1019.98) | -0.01 (-0.05 to 0.03) |
| Kuwait | 1249.42 (743.55 to 1963.29) | 1238.26 (707.21 to 2039.42) | 0.07 (-0.17 to 0.31) |
| Kyrgyzstan | 842.06 (522.24 to 1261.41) | 1007.32 (584.26 to 1592.94) | 0.72 (0.61 to 0.83) |
| Lao People's Democratic Republic | 622.23 (386.6 to 939.84) | 611.16 (364.08 to 954.39) | -0.11 (-0.62 to 0.41) |
| Latvia | 1061.16 (645.2 to 1586.76) | 1226.57 (706.52 to 1942.91) | 0.35 (0.1 to 0.6) |
| Lebanon | 1230.55 (755.33 to 1879.96) | 1912.82 (1065.68 to 3096.8) | 1.43 (1.32 to 1.53) |
| Lesotho | 1609.8 (971.82 to 2494.39) | 2030.26 (1190.98 to 3097.24) | 0.72 (0.64 to 0.81) |
| Liberia | 1315.2 (793.79 to 2000.77) | 1374.76 (799.69 to 2231.4) | 0.22 (0.19 to 0.25) |
| Libya | 1286.4 (761.52 to 1992.69) | 1441.79 (817.77 to 2316.73) | 0.46 (0.22 to 0.69) |
| Lithuania | 1097.91 (673.43 to 1640.1) | 1433.12 (837.56 to 2256.8) | 0.87 (0.82 to 0.92) |
| Luxembourg | 1246.01 (762.66 to 1860.18) | 1168.23 (707.73 to 1772.61) | -0.13 (-0.34 to 0.08) |
| Madagascar | 1137.18 (687.78 to 1732.3) | 1256 (710.34 to 1984.8) | 0.39 (0.3 to 0.47) |
| Malawi | 1010.89 (623.6 to 1520.44) | 1101.29 (650.84 to 1702.3) | 0.27 (0.24 to 0.3) |
| Malaysia | 691.13 (414.66 to 1057.23) | 816.09 (473.13 to 1285.97) | 0.37 (-0.32 to 1.06) |
| Maldives | 970.42 (594.3 to 1448.5) | 831.27 (496.82 to 1295.22) | -0.47 (-0.52 to -0.41) |
| Mali | 887.5 (539.34 to 1342.8) | 908.43 (536.91 to 1423.78) | 0.14 (0.08 to 0.2) |
| Malta | 991.75 (589.19 to 1538.78) | 1156.06 (663.49 to 1851.95) | 0.67 (0.45 to 0.9) |
| Marshall Islands | 632.55 (391.48 to 966.19) | 673.24 (405.18 to 1050.55) | 0.18 (0.13 to 0.23) |
| Mauritania | 834.26 (515.6 to 1253.9) | 878.72 (507.13 to 1389.44) | 0.21 (0.13 to 0.28) |
| Mauritius | 1215.71 (741.38 to 1821.32) | 1294.63 (744.65 to 2084.36) | 0.28 (0.06 to 0.5) |
| Mexico | 820.2 (523.33 to 1199.61) | 1454.88 (916.9 to 2159.53) | 2.09 (1.95 to 2.23) |
| Micronesia (Federated States of) | 650.15 (400.23 to 979.88) | 670.81 (390.51 to 1038.46) | 0.08 (0.03 to 0.12) |
| Monaco | 1293.02 (751.98 to 2093.35) | 1639.59 (897.03 to 2738.86) | 0.83 (0.8 to 0.87) |
| Mongolia | 1043.44 (633.03 to 1586.33) | 1039.75 (618.61 to 1636.19) | -0.05 (-0.15 to 0.05) |
| Montenegro | 747.27 (458.22 to 1117.09) | 978.59 (576.16 to 1530.96) | 0.95 (0.84 to 1.06) |
| Morocco | 1511.08 (907.1 to 2320.25) | 1739.34 (1003.43 to 2759.08) | 0.55 (0.21 to 0.89) |
| Mozambique | 1097.73 (665.55 to 1680.78) | 1293.72 (768.37 to 2060.99) | 0.53 (0.5 to 0.55) |
| Myanmar | 416.13 (251.59 to 638.2) | 525.32 (310.25 to 821.76) | 0.73 (0.5 to 0.95) |
| Namibia | 877.49 (533.86 to 1318.29) | 1157.85 (669.71 to 1851.8) | 0.82 (0.51 to 1.13) |
| Nauru | 701.29 (422.39 to 1095.26) | 788.37 (443.93 to 1305.02) | 0.35 (0.29 to 0.42) |
| Nepal | 1098.82 (672.85 to 1673.78) | 1534.59 (906.14 to 2434.23) | 1.14 (0.64 to 1.64) |
| Netherlands | 1179.2 (784.28 to 1650.97) | 1398.57 (807.42 to 2229.3) | 0.73 (0.53 to 0.92) |
| New Zealand | 1169.55 (728.07 to 1767.46) | 1290.62 (805.58 to 1966.62) | 0.3 (0.17 to 0.42) |
| Nicaragua | 909.41 (539.7 to 1404.09) | 1113.63 (653.62 to 1800.36) | 0.72 (0.36 to 1.09) |
| Niger | 1058.33 (645.02 to 1611.95) | 1051.01 (625.44 to 1651.39) | -0.03 (-0.08 to 0.01) |
| Nigeria | 997.89 (634.6 to 1471.88) | 871.35 (555.81 to 1280.77) | -0.36 (-0.58 to -0.15) |
| Niue | 706.18 (422 to 1107.19) | 788.15 (455.45 to 1262.85) | 0.33 (0.26 to 0.4) |
| North Macedonia | 690.13 (421.05 to 1042.75) | 911.98 (528.85 to 1417.11) | 0.97 (0.86 to 1.09) |
| Northern Mariana Islands | 543.14 (328.16 to 834.07) | 674.72 (401.05 to 1060.01) | 0.7 (0.62 to 0.78) |
| Norway | 1125.07 (717.53 to 1660.69) | 1393 (871.14 to 2074.45) | 0.66 (0.31 to 1) |
| Oman | 1193.29 (701.01 to 1866.32) | 1421.74 (791.39 to 2311.35) | 0.69 (0.63 to 0.75) |
| Pakistan | 1057.58 (665.51 to 1584.7) | 1142.53 (701.64 to 1737.27) | 0.43 (0.12 to 0.75) |
| Palau | 705.29 (419.82 to 1112.42) | 791.69 (454.18 to 1308.2) | 0.35 (0.28 to 0.41) |
| Palestine | 1681.85 (978.89 to 2632.86) | 1923.44 (1079.19 to 3141.52) | 0.54 (0.41 to 0.66) |
| Panama | 782.89 (465.81 to 1229.65) | 989.08 (556.7 to 1628.88) | 0.91 (0.5 to 1.32) |
| Papua New Guinea | 722.75 (443.96 to 1107.48) | 720.32 (417.67 to 1122.6) | -0.03 (-0.09 to 0.03) |
| Paraguay | 972.39 (579.48 to 1503.74) | 1343.83 (769.79 to 2143.43) | 1.11 (1.03 to 1.19) |
| Peru | 605.36 (363.55 to 924) | 752.59 (442.61 to 1212.13) | 0.85 (0.29 to 1.42) |
| Philippines | 679.43 (435.85 to 993.71) | 769.43 (491.31 to 1131.03) | 0.48 (0.39 to 0.56) |
| Poland | 499.46 (321.65 to 726.06) | 597.77 (382.01 to 880.95) | 0.63 (0.56 to 0.71) |
| Portugal | 1730.88 (1063.47 to 2626.8) | 1946.39 (1095.03 to 3084.17) | 0.45 (-0.31 to 1.23) |
| Puerto Rico | 672.53 (404.73 to 1029.11) | 808.74 (451.79 to 1271.11) | 0.67 (0.38 to 0.96) |
| Qatar | 1383.85 (822.49 to 2144.33) | 1399.2 (802.71 to 2286.54) | 0.09 (-0.14 to 0.33) |
| Republic of Korea | 601.41 (383.34 to 870.88) | 720.17 (440.65 to 1098.91) | 0.66 (0.53 to 0.8) |
| Republic of Moldova | 916.18 (562.71 to 1379.99) | 992.76 (573.7 to 1587.68) | 0.33 (0.23 to 0.43) |
| Romania | 670.54 (404.87 to 1007.41) | 856.96 (508.46 to 1351.34) | 0.88 (0.79 to 0.97) |
| Russian Federation | 773.08 (490.02 to 1137.32) | 997.99 (627.67 to 1482.82) | 0.85 (0.43 to 1.26) |
| Rwanda | 1487.51 (894.63 to 2289.63) | 1476.32 (846.76 to 2363.8) | -0.04 (-0.13 to 0.06) |
| Saint Kitts and Nevis | 1050.86 (609 to 1696.13) | 1291.13 (711.21 to 2163.94) | 0.75 (0.68 to 0.82) |
| Saint Lucia | 829.52 (492.42 to 1279.8) | 1126.35 (635.25 to 1846.24) | 0.99 (0.93 to 1.06) |
| Saint Vincent and the Grenadines | 828.44 (485.17 to 1278.63) | 1066.26 (601.21 to 1723.3) | 0.84 (0.8 to 0.88) |
| Samoa | 626.76 (387.9 to 945.57) | 653.55 (382.13 to 1025.33) | 0.1 (0.04 to 0.16) |
| San Marino | 1301.22 (750.49 to 2101.54) | 1672.22 (907.89 to 2805.44) | 1.02 (0.97 to 1.08) |
| Sao Tome and Principe | 950.92 (569.11 to 1440.35) | 1027.46 (602.74 to 1647.28) | 0.31 (0.19 to 0.43) |
| Saudi Arabia | 1205.81 (714.05 to 1890.17) | 1383.82 (815.81 to 2199.79) | 0.67 (0.32 to 1.01) |
| Senegal | 891.09 (542.03 to 1342.26) | 1048.46 (607.3 to 1634.75) | 0.59 (0.46 to 0.73) |
| Serbia | 796.24 (490.11 to 1180.62) | 865.36 (505.35 to 1361.97) | 0.32 (0.26 to 0.39) |
| Seychelles | 552.99 (337.79 to 837.63) | 682.31 (394.42 to 1089.03) | 0.66 (0.62 to 0.71) |
| Sierra Leone | 1037.48 (627.24 to 1568.41) | 1136 (657.17 to 1822.36) | 0.34 (0.28 to 0.4) |
| Singapore | 990.39 (641.14 to 1422.4) | 660.4 (391.27 to 1022.37) | -1.29 (-1.56 to -1.02) |
| Slovakia | 739.37 (455.71 to 1115.25) | 920.91 (529.73 to 1478.06) | 0.66 (0.43 to 0.9) |
| Slovenia | 1044.23 (647.99 to 1571.23) | 999.97 (598.32 to 1596.62) | -0.04 (-0.17 to 0.08) |
| Solomon Islands | 702.28 (427.37 to 1070.82) | 730.11 (435.28 to 1137.36) | 0.12 (0.04 to 0.19) |
| Somalia | 1188.74 (721.15 to 1795.98) | 1453.96 (837.71 to 2312.22) | 0.66 (0.6 to 0.73) |
| South Africa | 1151.3 (756.68 to 1661.71) | 1429.31 (920.11 to 2093.27) | 0.88 (0.58 to 1.18) |
| South Sudan | 1140.95 (690.5 to 1711.21) | 1231.81 (702.92 to 1969.91) | 0.33 (0.18 to 0.48) |
| Spain | 1288.11 (858.2 to 1808.1) | 1897.46 (1137.06 to 2989.99) | 1.52 (1.24 to 1.81) |
| Sri Lanka | 847.29 (531.37 to 1259.18) | 806.41 (493.23 to 1256.48) | -0.2 (-0.37 to -0.03) |
| Sudan | 1271.73 (752.63 to 1980.1) | 1379.35 (812.23 to 2211.9) | 0.26 (-0.02 to 0.54) |
| Suriname | 1275.25 (789.56 to 1936.93) | 1839.78 (1065.99 to 2958.26) | 1.19 (1.08 to 1.3) |
| Sweden | 1534.52 (987.34 to 2233.39) | 1833.59 (1145.55 to 2752.3) | 0.6 (0.22 to 0.97) |
| Switzerland | 1433.97 (908.95 to 2139.17) | 1416.07 (834.2 to 2208.67) | -0.03 (-0.14 to 0.09) |
| Syrian Arab Republic | 1185.23 (690.53 to 1857.45) | 1385.84 (785.25 to 2261.57) | 0.6 (0.5 to 0.71) |
| Taiwan (Province of China) | 494.61 (305.73 to 736.8) | 573.25 (337.38 to 883.74) | 0.48 (0.44 to 0.53) |
| Tajikistan | 728.32 (441.75 to 1097.48) | 847.82 (501.44 to 1352.89) | 0.51 (0.43 to 0.58) |
| Thailand | 648.6 (402.77 to 973.18) | 692.43 (410.25 to 1082.52) | 0.19 (0.16 to 0.21) |
| Timor-Leste | 703.42 (430.33 to 1064.68) | 701.19 (408.02 to 1103.33) | -0.03 (-0.09 to 0.03) |
| Togo | 1067.22 (648.25 to 1602.87) | 1123.64 (656.82 to 1751.85) | 0.21 (0.08 to 0.35) |
| Tokelau | 704.26 (422.09 to 1110.22) | 789.77 (453.17 to 1281.81) | 0.35 (0.29 to 0.41) |
| Tonga | 605.09 (374.02 to 914.25) | 641.23 (391.8 to 995.49) | 0.16 (0.11 to 0.21) |
| Trinidad and Tobago | 1128.15 (686.54 to 1706.63) | 1422.79 (819.24 to 2336.64) | 0.69 (0.59 to 0.79) |
| Tunisia | 1452.19 (859.81 to 2261.87) | 1891.13 (1076.62 to 3086.41) | 0.86 (0.8 to 0.93) |
| Turkey | 1256.23 (820.5 to 1786.11) | 1495.08 (826.51 to 2448.84) | 0.65 (0.38 to 0.92) |
| Turkmenistan | 768.44 (468.29 to 1159.77) | 867.85 (503.37 to 1379.95) | 0.46 (0.42 to 0.51) |
| Tuvalu | 701.36 (414.24 to 1103.03) | 784.16 (455.18 to 1274.9) | 0.33 (0.27 to 0.39) |
| Uganda | 1649.28 (1002.22 to 2532.89) | 1803.69 (1039.96 to 2881.94) | 0.32 (0.16 to 0.47) |
| Ukraine | 1060.67 (662.7 to 1580.92) | 1273.35 (768.55 to 1971.37) | 0.56 (0.37 to 0.76) |
| United Arab Emirates | 1211.94 (727.69 to 1879.79) | 1303.06 (747.1 to 2114.92) | 0.33 (0.29 to 0.38) |
| United Kingdom | 1421.17 (902.34 to 2095.25) | 1556.88 (980.24 to 2332.86) | 0.55 (0.18 to 0.93) |
| United Republic of Tanzania | 1121.29 (687.62 to 1685.16) | 1224.06 (713.36 to 1901.39) | 0.32 (0.29 to 0.34) |
| United States of America | 1206.85 (798.34 to 1735.92) | 1982.29 (1326.01 to 2844.07) | 1.7 (1.37 to 2.03) |
| United States Virgin Islands | 843.28 (504.73 to 1313.81) | 1050.48 (587.73 to 1656.63) | 0.77 (0.64 to 0.9) |
| Uruguay | 911.39 (550.34 to 1398.16) | 1335.28 (769.95 to 2160.47) | 1.23 (0.88 to 1.58) |
| Uzbekistan | 790.4 (492.23 to 1178.43) | 864.57 (512.96 to 1357.1) | 0.36 (0.24 to 0.48) |
| Vanuatu | 694.02 (425.95 to 1055.65) | 717.93 (422.89 to 1114.82) | 0.1 (0.04 to 0.16) |
| Venezuela (Bolivarian Republic of) | 828.65 (486.8 to 1284.14) | 923.24 (519.4 to 1480.02) | 0.35 (0.2 to 0.5) |
| Viet Nam | 615.34 (378.49 to 926.16) | 657.25 (388.72 to 1021.77) | 0.21 (0.04 to 0.38) |
| Yemen | 1523.46 (901.25 to 2386.08) | 1517.75 (848.41 to 2442.57) | -0.13 (-0.18 to -0.07) |
| Zambia | 956.99 (592.65 to 1434.37) | 1082.81 (634.87 to 1684.12) | 0.37 (0.31 to 0.43) |
| Zimbabwe | 725.87 (443.41 to 1095.82) | 856.27 (513.93 to 1349.79) | 0.51 (0.34 to 0.69) |

Supplementary Table 4. APCs of 7 super regions in ASIR of depressive disorders in WCBA. Abbreviations: WCBA, women of childbearing age; APC, annual percentage change; CI, confidence interval; ASIR, age-standardized incidence rate.

| Location | Year. Start | Year. End | APC (95% CI) | p-value |
| --- | --- | --- | --- | --- |
| Central Europe Eastern Europe and Central Asia | 1990 | 1994 | 0.43  (0.1 to 0.76) | 0.012 |
| Central Europe Eastern Europe and Central Asia | 1994 | 2010 | -0.86  (-0.9 to -0.82) | 0 |
| Central Europe Eastern Europe and Central Asia | 2010 | 2019 | 0.44  (0.32 to 0.55) | 0 |
| Central Europe Eastern Europe and Central Asia | 2019 | 2021 | 16.69  (15.41 to 17.99) | 0 |
| Global | 1990 | 2005 | -0.01  (-0.08 to 0.06) | 0.771 |
| Global | 2005 | 2010 | -2.08  (-2.55 to -1.61) | 0 |
| Global | 2010 | 2019 | 0.41  (0.23 to 0.59) | 0 |
| Global | 2019 | 2021 | 12.35  (10.38 to 14.36) | 0 |
| High-income | 1990 | 2000 | 1.65  (1.49 to 1.82) | 0 |
| High-income | 2000 | 2019 | -0.18  (-0.25 to -0.11) | 0 |
| High-income | 2019 | 2021 | 16.33  (13.53 to 19.2) | 0 |
| Latin America and Caribbean | 1990 | 2003 | 0.77  (0.38 to 1.17) | 0.001 |
| Latin America and Caribbean | 2003 | 2018 | -1  (-1.33 to -0.66) | 0 |
| Latin America and Caribbean | 2018 | 2021 | 11.96  (7.64 to 16.46) | 0 |
| North Africa and Middle East | 1990 | 2018 | -0.01  (-0.1 to 0.08) | 0.791 |
| North Africa and Middle East | 2018 | 2021 | 6.82  (3.53 to 10.22) | 0 |
| South Asia | 1990 | 1994 | 2.49  (2.12 to 2.87) | 0 |
| South Asia | 1994 | 2006 | -0.33  (-0.41 to -0.26) | 0 |
| South Asia | 2006 | 2010 | -6.18  (-6.7 to -5.67) | 0 |
| South Asia | 2010 | 2019 | -0.09  (-0.21 to 0.03) | 0.144 |
| South Asia | 2019 | 2021 | 14.17  (12.82 to 15.53) | 0 |
| Southeast Asia East Asia and Oceania | 1990 | 1995 | -1.03  (-1.62 to -0.44) | 0.002 |
| Southeast Asia East Asia and Oceania | 1995 | 1999 | -2.95  (-4.17 to -1.71) | 0 |
| Southeast Asia East Asia and Oceania | 1999 | 2010 | -1.63  (-1.82 to -1.45) | 0 |
| Southeast Asia East Asia and Oceania | 2010 | 2019 | 0.74  (0.45 to 1.03) | 0 |
| Southeast Asia East Asia and Oceania | 2019 | 2021 | 4.6  (1.57 to 7.72) | 0.005 |
| Sub-Saharan Africa | 1990 | 2004 | -0.05  (-0.09 to -0.01) | 0.012 |
| Sub-Saharan Africa | 2004 | 2012 | -0.9  (-1 to -0.79) | 0 |
| Sub-Saharan Africa | 2012 | 2019 | 0.2  (0.05 to 0.35) | 0.013 |
| Sub-Saharan Africa | 2019 | 2021 | 7.52  (6.54 to 8.51) | 0 |

Supplementary Table 5. APCs of 7 super regions in ASPR of depressive disorders in WCBA. Abbreviations: WCBA, women of childbearing age; APC, annual percentage change; CI, confidence interval; ASPR, age-standardized prevalence rate.

| Location | Year. Start | Year. End | APC (95% CI) | p-value |
| --- | --- | --- | --- | --- |
| Central Europe Eastern Europe and Central Asia | 1990 | 1994 | 0.27  (0.04 to 0.5) | 0.025 |
| Central Europe Eastern Europe and Central Asia | 1994 | 2010 | -0.58  (-0.61 to -0.55) | 0 |
| Central Europe Eastern Europe and Central Asia | 2010 | 2019 | 0.3  (0.22 to 0.38) | 0 |
| Central Europe Eastern Europe and Central Asia | 2019 | 2021 | 11.24  (10.37 to 12.11) | 0 |
| Global | 1990 | 2005 | -0.05  (-0.11 to 0) | 0.052 |
| Global | 2005 | 2010 | -1.42  (-1.8 to -1.03) | 0 |
| Global | 2010 | 2019 | 0.32  (0.18 to 0.46) | 0 |
| Global | 2019 | 2021 | 8.88  (7.34 to 10.45) | 0 |
| High-income | 1990 | 1999 | 1  (0.97 to 1.03) | 0 |
| High-income | 1999 | 2019 | -0.08  (-0.09 to -0.07) | 0 |
| High-income | 2019 | 2021 | 13.27  (12.77 to 13.78) | 0 |
| Latin America and Caribbean | 1990 | 2003 | 0.62  (0.3 to 0.94) | 0 |
| Latin America and Caribbean | 2003 | 2018 | -0.81  (-1.08 to -0.54) | 0 |
| Latin America and Caribbean | 2018 | 2021 | 9.53  (6.1 to 13.07) | 0 |
| North Africa and Middle East | 1990 | 2000 | -0.32  (-0.42 to -0.23) | 0 |
| North Africa and Middle East | 2000 | 2019 | 0.17  (0.13 to 0.21) | 0 |
| North Africa and Middle East | 2019 | 2021 | 7.78  (6.2 to 9.39) | 0 |
| South Asia | 1990 | 1994 | 1.81  (1.51 to 2.12) | 0 |
| South Asia | 1994 | 2006 | -0.24  (-0.3 to -0.18) | 0 |
| South Asia | 2006 | 2010 | -4.46  (-4.89 to -4.03) | 0 |
| South Asia | 2010 | 2019 | -0.04  (-0.14 to 0.06) | 0.394 |
| South Asia | 2019 | 2021 | 10.1  (9.02 to 11.2) | 0 |
| Southeast Asia East Asia and Oceania | 1990 | 1995 | -0.63  (-0.96 to -0.29) | 0.001 |
| Southeast Asia East Asia and Oceania | 1995 | 1999 | -1.94  (-2.66 to -1.22) | 0 |
| Southeast Asia East Asia and Oceania | 1999 | 2010 | -0.97  (-1.08 to -0.86) | 0 |
| Southeast Asia East Asia and Oceania | 2010 | 2019 | 0.27  (0.1 to 0.43) | 0.003 |
| Southeast Asia East Asia and Oceania | 2019 | 2021 | 3.65  (2 to 5.33) | 0 |
| Sub-Saharan Africa | 1990 | 2004 | -0.05  (-0.08 to -0.03) | 0 |
| Sub-Saharan Africa | 2004 | 2012 | -0.62  (-0.69 to -0.55) | 0 |
| Sub-Saharan Africa | 2012 | 2019 | 0.13  (0.04 to 0.22) | 0.009 |
| Sub-Saharan Africa | 2019 | 2021 | 5.18  (4.57 to 5.79) | 0 |

Supplementary Table 6. APCs of 7 super regions in ASDR of depressive disorders in WCBA. Abbreviations: WCBA, women of childbearing age; APC, annual percentage change; CI, confidence interval; ASDR, age-standardized DALYs rate.

| Location | Year. Start | Year. End | APC (95% CI) | p-value |
| --- | --- | --- | --- | --- |
| Central Europe Eastern Europe and Central Asia | 1990 | 1994 | 0.33  (0.03 to 0.63) | 0.035 |
| Central Europe Eastern Europe and Central Asia | 1994 | 2010 | -0.71  (-0.75 to -0.67) | 0 |
| Central Europe Eastern Europe and Central Asia | 2010 | 2019 | 0.4  (0.3 to 0.51) | 0 |
| Central Europe Eastern Europe and Central Asia | 2019 | 2021 | 13.76  (12.65 to 14.89) | 0 |
| Global | 1990 | 2005 | -0.03  (-0.09 to 0.03) | 0.254 |
| Global | 2005 | 2010 | -1.72  (-2.16 to -1.29) | 0 |
| Global | 2010 | 2019 | 0.37  (0.22 to 0.53) | 0 |
| Global | 2019 | 2021 | 10.42  (8.82 to 12.05) | 0 |
| High-income | 1990 | 2000 | 1.27  (1.18 to 1.36) | 0 |
| High-income | 2000 | 2019 | -0.13  (-0.17 to -0.09) | 0 |
| High-income | 2019 | 2021 | 14.66  (13.29 to 16.05) | 0 |
| Latin America and Caribbean | 1990 | 2003 | 0.7  (0.34 to 1.06) | 0.001 |
| Latin America and Caribbean | 2003 | 2018 | -0.89  (-1.21 to -0.58) | 0 |
| Latin America and Caribbean | 2018 | 2021 | 10.65  (6.86 to 14.58) | 0 |
| North Africa and Middle East | 1990 | 2018 | 0  (-0.08 to 0.09) | 0.922 |
| North Africa and Middle East | 2018 | 2021 | 5.97  (3.1 to 8.93) | 0 |
| South Asia | 1990 | 1994 | 2.15  (1.82 to 2.48) | 0 |
| South Asia | 1994 | 2006 | -0.25  (-0.32 to -0.18) | 0 |
| South Asia | 2006 | 2010 | -5.31  (-5.78 to -4.83) | 0 |
| South Asia | 2010 | 2019 | -0.04  (-0.15 to 0.07) | 0.473 |
| South Asia | 2019 | 2021 | 11.98  (10.83 to 13.14) | 0 |
| Southeast Asia East Asia and Oceania | 1990 | 1995 | -0.81  (-1.25 to -0.36) | 0.001 |
| Southeast Asia East Asia and Oceania | 1995 | 1999 | -2.42  (-3.37 to -1.46) | 0 |
| Southeast Asia East Asia and Oceania | 1999 | 2010 | -1.25  (-1.4 to -1.1) | 0 |
| Southeast Asia East Asia and Oceania | 2010 | 2019 | 0.46  (0.24 to 0.68) | 0 |
| Southeast Asia East Asia and Oceania | 2019 | 2021 | 3.98  (1.9 to 6.1) | 0.001 |
| Sub-Saharan Africa | 1990 | 2001 | -0.02  (-0.23 to 0.19) | 0.852 |
| Sub-Saharan Africa | 2001 | 2018 | -0.41  (-0.53 to -0.29) | 0 |
| Sub-Saharan Africa | 2018 | 2021 | 4.63  (2.96 to 6.32) | 0 |

Supplementary Table 7. APCs of 204 countries in ASIR of depressive disorders in WCBA. Abbreviations: WCBA, women of childbearing age; APC, annual percentage change; CI, confidence interval; ASIR, age-standardized incidence rate.

| Location | Year. Start | Year. End | APC (95% CI) | p-value |
| --- | --- | --- | --- | --- |
| Afghanistan | 1990 | 2019 | -0.07  (-0.09 to -0.06) | 0 |
| Afghanistan | 2019 | 2021 | 12.08  (10.72 to 13.45) | 0 |
| Albania | 1990 | 2003 | 0.38  (0.28 to 0.48) | 0 |
| Albania | 2003 | 2019 | -0.12  (-0.2 to -0.04) | 0.006 |
| Albania | 2019 | 2021 | 21.57  (18.69 to 24.53) | 0 |
| Algeria | 1990 | 2017 | -0.25  (-0.34 to -0.15) | 0 |
| Algeria | 2017 | 2021 | 5.05  (3.09 to 7.05) | 0 |
| American Samoa | 1990 | 1994 | -0.67  (-1.05 to -0.28) | 0.002 |
| American Samoa | 1994 | 2014 | -0.13  (-0.16 to -0.09) | 0 |
| American Samoa | 2014 | 2019 | 0.72  (0.32 to 1.11) | 0.001 |
| American Samoa | 2019 | 2021 | 6.88  (5.31 to 8.48) | 0 |
| Andorra | 1990 | 2018 | -0.23  (-0.35 to -0.1) | 0.001 |
| Andorra | 2018 | 2021 | 12.21  (7.53 to 17.09) | 0 |
| Angola | 1990 | 2019 | -0.17  (-0.21 to -0.14) | 0 |
| Angola | 2019 | 2021 | 9.02  (6.34 to 11.78) | 0 |
| Antigua and Barbuda | 1990 | 1994 | -1.45  (-1.56 to -1.34) | 0 |
| Antigua and Barbuda | 1994 | 2006 | -0.05  (-0.08 to -0.03) | 0 |
| Antigua and Barbuda | 2006 | 2010 | -1.53  (-1.7 to -1.35) | 0 |
| Antigua and Barbuda | 2010 | 2019 | 1.57  (1.53 to 1.61) | 0 |
| Antigua and Barbuda | 2019 | 2021 | 14.03  (13.56 to 14.51) | 0 |
| Argentina | 1990 | 2019 | -0.32  (-0.4 to -0.23) | 0 |
| Argentina | 2019 | 2021 | 18.47  (11.17 to 26.26) | 0 |
| Armenia | 1990 | 2011 | -0.17  (-0.23 to -0.11) | 0 |
| Armenia | 2011 | 2019 | 1.07  (0.76 to 1.38) | 0 |
| Armenia | 2019 | 2021 | 20.2  (16.94 to 23.55) | 0 |
| Australia | 1990 | 2000 | 0.85  (0.78 to 0.91) | 0 |
| Australia | 2000 | 2005 | 2.22  (1.96 to 2.47) | 0 |
| Australia | 2005 | 2019 | -1.08  (-1.13 to -1.03) | 0 |
| Australia | 2019 | 2021 | 6.28  (4.84 to 7.75) | 0 |
| Austria | 1990 | 1995 | -0.35  (-0.8 to 0.1) | 0.116 |
| Austria | 1995 | 1999 | -2.78  (-3.71 to -1.85) | 0 |
| Austria | 1999 | 2011 | -1.2  (-1.33 to -1.07) | 0 |
| Austria | 2011 | 2019 | 0.69  (0.43 to 0.96) | 0 |
| Austria | 2019 | 2021 | 14.34  (11.52 to 17.24) | 0 |
| Azerbaijan | 1990 | 1994 | -1.23  (-1.56 to -0.9) | 0 |
| Azerbaijan | 1994 | 2013 | -0.18  (-0.21 to -0.14) | 0 |
| Azerbaijan | 2013 | 2019 | 1.59  (1.35 to 1.84) | 0 |
| Azerbaijan | 2019 | 2021 | 17.44  (16.02 to 18.87) | 0 |
| Bahamas | 1990 | 2012 | -0.29  (-0.36 to -0.23) | 0 |
| Bahamas | 2012 | 2019 | 1.57  (1.08 to 2.06) | 0 |
| Bahamas | 2019 | 2021 | 16.5  (12.66 to 20.46) | 0 |
| Bahrain | 1990 | 2000 | 0.12  (0.05 to 0.18) | 0.002 |
| Bahrain | 2000 | 2014 | -1.16  (-1.2 to -1.11) | 0 |
| Bahrain | 2014 | 2019 | 0.4  (0.1 to 0.7) | 0.011 |
| Bahrain | 2019 | 2021 | 12.12  (10.88 to 13.36) | 0 |
| Bangladesh | 1990 | 2019 | -0.23  (-0.25 to -0.21) | 0 |
| Bangladesh | 2019 | 2021 | 11.66  (10.13 to 13.21) | 0 |
| Barbados | 1990 | 1995 | -0.99  (-1.16 to -0.83) | 0 |
| Barbados | 1995 | 2006 | 0.09  (0.04 to 0.15) | 0.003 |
| Barbados | 2006 | 2010 | -0.58  (-0.94 to -0.22) | 0.003 |
| Barbados | 2010 | 2019 | 0.91  (0.83 to 0.99) | 0 |
| Barbados | 2019 | 2021 | 16.62  (15.63 to 17.62) | 0 |
| Belarus | 1990 | 1995 | 0.82  (0.7 to 0.94) | 0 |
| Belarus | 1995 | 2004 | 0.29  (0.23 to 0.34) | 0 |
| Belarus | 2004 | 2019 | -0.53  (-0.55 to -0.5) | 0 |
| Belarus | 2019 | 2021 | 25.7  (24.89 to 26.52) | 0 |
| Belgium | 1990 | 1999 | 0.15  (-0.31 to 0.61) | 0.51 |
| Belgium | 1999 | 2010 | 1.69  (1.19 to 2.19) | 0 |
| Belgium | 2010 | 2018 | -1.22  (-2.32 to -0.1) | 0.034 |
| Belgium | 2018 | 2021 | 10.21  (4.58 to 16.16) | 0.001 |
| Belize | 1990 | 2019 | 0.16  (0.11 to 0.21) | 0 |
| Belize | 2019 | 2021 | 15.64  (11.4 to 20.05) | 0 |
| Benin | 1990 | 2002 | 0.1  (0.06 to 0.14) | 0 |
| Benin | 2002 | 2012 | -0.15  (-0.22 to -0.09) | 0 |
| Benin | 2012 | 2019 | 0.24  (0.12 to 0.36) | 0.001 |
| Benin | 2019 | 2021 | 5.19  (4.28 to 6.1) | 0 |
| Bermuda | 1990 | 2002 | -0.83  (-0.86 to -0.8) | 0 |
| Bermuda | 2002 | 2010 | -1.41  (-1.49 to -1.34) | 0 |
| Bermuda | 2010 | 2019 | 0.48  (0.42 to 0.54) | 0 |
| Bermuda | 2019 | 2021 | 15.61  (14.8 to 16.42) | 0 |
| Bhutan | 1990 | 2014 | -0.67  (-0.75 to -0.58) | 0 |
| Bhutan | 2014 | 2021 | 1.51  (0.9 to 2.13) | 0 |
| Bolivia (Plurinational State of) | 1990 | 2019 | -0.44  (-0.47 to -0.4) | 0 |
| Bolivia (Plurinational State of) | 2019 | 2021 | 29.21  (26.03 to 32.47) | 0 |
| Bosnia and Herzegovina | 1990 | 2001 | -0.26  (-0.33 to -0.19) | 0 |
| Bosnia and Herzegovina | 2001 | 2013 | -2.17  (-2.24 to -2.1) | 0 |
| Bosnia and Herzegovina | 2013 | 2019 | 0.28  (0 to 0.55) | 0.047 |
| Bosnia and Herzegovina | 2019 | 2021 | 20.36  (18.67 to 22.07) | 0 |
| Botswana | 1990 | 2001 | 0.77  (0.67 to 0.88) | 0 |
| Botswana | 2001 | 2019 | -0.4  (-0.45 to -0.34) | 0 |
| Botswana | 2019 | 2021 | 14.52  (12.38 to 16.69) | 0 |
| Brazil | 1990 | 2005 | 1.46  (1.23 to 1.69) | 0 |
| Brazil | 2005 | 2010 | -5.21  (-6.65 to -3.74) | 0 |
| Brazil | 2010 | 2019 | -0.77  (-1.28 to -0.26) | 0.005 |
| Brazil | 2019 | 2021 | 18.87  (12.41 to 25.7) | 0 |
| Brunei Darussalam | 1990 | 1994 | -1.53  (-1.8 to -1.26) | 0 |
| Brunei Darussalam | 1994 | 2005 | -0.07  (-0.14 to -0.01) | 0.035 |
| Brunei Darussalam | 2005 | 2011 | -1  (-1.19 to -0.81) | 0 |
| Brunei Darussalam | 2011 | 2019 | 1.81  (1.69 to 1.93) | 0 |
| Brunei Darussalam | 2019 | 2021 | 9.79  (8.68 to 10.9) | 0 |
| Bulgaria | 1990 | 1998 | -0.24  (-0.31 to -0.17) | 0 |
| Bulgaria | 1998 | 2003 | -0.92  (-1.12 to -0.72) | 0 |
| Bulgaria | 2003 | 2010 | -1.85  (-1.96 to -1.74) | 0 |
| Bulgaria | 2010 | 2019 | 0.09  (0.02 to 0.17) | 0.02 |
| Bulgaria | 2019 | 2021 | 27.7  (26.64 to 28.78) | 0 |
| Burkina Faso | 1990 | 1999 | -1  (-1.41 to -0.59) | 0 |
| Burkina Faso | 1999 | 2005 | 1.84  (0.88 to 2.81) | 0.001 |
| Burkina Faso | 2005 | 2010 | -0.66  (-1.95 to 0.63) | 0.294 |
| Burkina Faso | 2010 | 2014 | 2.61  (0.42 to 4.85) | 0.022 |
| Burkina Faso | 2014 | 2021 | -1.01  (-1.73 to -0.29) | 0.009 |
| Burundi | 1990 | 1996 | -0.12  (-0.24 to 0.01) | 0.067 |
| Burundi | 1996 | 2011 | -1.47  (-1.51 to -1.44) | 0 |
| Burundi | 2011 | 2019 | 0  (-0.1 to 0.09) | 0.977 |
| Burundi | 2019 | 2021 | 10.62  (9.64 to 11.61) | 0 |
| Cabo Verde | 1990 | 2019 | 0.22  (0.17 to 0.27) | 0 |
| Cabo Verde | 2019 | 2021 | 14.33  (10.36 to 18.45) | 0 |
| Cambodia | 1990 | 1996 | 0.23  (0.03 to 0.44) | 0.029 |
| Cambodia | 1996 | 2010 | -1.59  (-1.65 to -1.53) | 0 |
| Cambodia | 2010 | 2019 | -0.43  (-0.56 to -0.31) | 0 |
| Cambodia | 2019 | 2021 | 13.22  (11.64 to 14.82) | 0 |
| Cameroon | 1990 | 2003 | 0.2  (0.19 to 0.22) | 0 |
| Cameroon | 2003 | 2015 | -0.24  (-0.26 to -0.21) | 0 |
| Cameroon | 2015 | 2019 | 0.39  (0.23 to 0.55) | 0 |
| Cameroon | 2019 | 2021 | 4.55  (4.15 to 4.96) | 0 |
| Canada | 1990 | 2000 | 1.36  (1.27 to 1.46) | 0 |
| Canada | 2000 | 2009 | -2.06  (-2.17 to -1.95) | 0 |
| Canada | 2009 | 2019 | 1.01  (0.9 to 1.11) | 0 |
| Canada | 2019 | 2021 | 17.63  (15.51 to 19.79) | 0 |
| Central African Republic | 1990 | 2021 | -0.01  (-0.11 to 0.1) | 0.907 |
| Chad | 1990 | 2004 | 0.38  (0.2 to 0.56) | 0 |
| Chad | 2004 | 2017 | -0.33  (-0.55 to -0.11) | 0.005 |
| Chad | 2017 | 2021 | 2.13  (0.63 to 3.64) | 0.007 |
| Chile | 1990 | 2005 | -0.36  (-0.4 to -0.32) | 0 |
| Chile | 2005 | 2010 | -2.42  (-2.76 to -2.07) | 0 |
| Chile | 2010 | 2019 | 0.22  (0.08 to 0.36) | 0.004 |
| Chile | 2019 | 2021 | 16.45  (14.59 to 18.34) | 0 |
| China | 1990 | 1995 | -1.17  (-2.33 to 0.01) | 0.051 |
| China | 1995 | 1999 | -3.65  (-6.01 to -1.24) | 0.005 |
| China | 1999 | 2010 | -2.14  (-2.5 to -1.78) | 0 |
| China | 2010 | 2021 | 0.68  (0.33 to 1.03) | 0.001 |
| Colombia | 1990 | 2005 | 1.21  (0.92 to 1.5) | 0 |
| Colombia | 2005 | 2011 | -6.25  (-7.32 to -5.17) | 0 |
| Colombia | 2011 | 2018 | -0.56  (-1.44 to 0.33) | 0.203 |
| Colombia | 2018 | 2021 | 6.88  (3.15 to 10.75) | 0.001 |
| Comoros | 1990 | 2002 | -0.16  (-0.21 to -0.11) | 0 |
| Comoros | 2002 | 2012 | -0.55  (-0.63 to -0.48) | 0 |
| Comoros | 2012 | 2019 | 0.37  (0.23 to 0.51) | 0 |
| Comoros | 2019 | 2021 | 10.75  (9.64 to 11.88) | 0 |
| Congo | 1990 | 1995 | -0.2  (-1.2 to 0.81) | 0.687 |
| Congo | 1995 | 2000 | 2  (0.71 to 3.3) | 0.004 |
| Congo | 2000 | 2008 | -0.48  (-0.96 to 0.01) | 0.054 |
| Congo | 2008 | 2018 | -1.41  (-1.77 to -1.05) | 0 |
| Congo | 2018 | 2021 | 6.43  (3.84 to 9.1) | 0 |
| Cook Islands | 1990 | 2019 | 0.01  (-0.01 to 0.03) | 0.225 |
| Cook Islands | 2019 | 2021 | 6.83  (5.54 to 8.14) | 0 |
| Costa Rica | 1990 | 2019 | 0.25  (0.22 to 0.28) | 0 |
| Costa Rica | 2019 | 2021 | 14.95  (12.49 to 17.47) | 0 |
| Coted'Ivoire | 1990 | 2012 | -0.28  (-0.34 to -0.23) | 0 |
| Coted'Ivoire | 2012 | 2021 | 1.01  (0.76 to 1.27) | 0 |
| Croatia | 1990 | 1996 | -0.52  (-0.6 to -0.43) | 0 |
| Croatia | 1996 | 2009 | -1.84  (-1.87 to -1.81) | 0 |
| Croatia | 2009 | 2019 | -0.02  (-0.07 to 0.03) | 0.487 |
| Croatia | 2019 | 2021 | 16.48  (15.7 to 17.26) | 0 |
| Cuba | 1990 | 1995 | -1.56  (-1.76 to -1.37) | 0 |
| Cuba | 1995 | 1999 | -2.71  (-3.13 to -2.29) | 0 |
| Cuba | 1999 | 2010 | -3.32  (-3.39 to -3.25) | 0 |
| Cuba | 2010 | 2019 | 0.11  (0.01 to 0.22) | 0.039 |
| Cuba | 2019 | 2021 | 15.96  (14.55 to 17.38) | 0 |
| Cyprus | 1990 | 1994 | -1.29  (-1.54 to -1.03) | 0 |
| Cyprus | 1994 | 2005 | -0.06  (-0.12 to 0) | 0.056 |
| Cyprus | 2005 | 2011 | -0.89  (-1.08 to -0.71) | 0 |
| Cyprus | 2011 | 2019 | 1.53  (1.41 to 1.64) | 0 |
| Cyprus | 2019 | 2021 | 11.02  (9.96 to 12.1) | 0 |
| Czechia | 1990 | 1995 | -0.02  (-0.2 to 0.16) | 0.824 |
| Czechia | 1995 | 2004 | -1.64  (-1.72 to -1.56) | 0 |
| Czechia | 2004 | 2015 | -0.95  (-1.02 to -0.89) | 0 |
| Czechia | 2015 | 2019 | 0.88  (0.43 to 1.32) | 0.001 |
| Czechia | 2019 | 2021 | 16.95  (15.68 to 18.23) | 0 |
| Democratic People's Republic of Korea | 1990 | 1995 | -0.29  (-0.33 to -0.24) | 0 |
| Democratic People's Republic of Korea | 1995 | 2005 | -0.8  (-0.82 to -0.78) | 0 |
| Democratic People's Republic of Korea | 2005 | 2010 | -1.02  (-1.08 to -0.96) | 0 |
| Democratic People's Republic of Korea | 2010 | 2019 | -0.05  (-0.08 to -0.03) | 0 |
| Democratic People's Republic of Korea | 2019 | 2021 | 4.05  (3.79 to 4.32) | 0 |
| Democratic Republic of the Congo | 1990 | 2018 | -0.17  (-0.25 to -0.1) | 0 |
| Democratic Republic of the Congo | 2018 | 2021 | 4.17  (1.69 to 6.72) | 0.002 |
| Denmark | 1990 | 1995 | -1.09  (-1.22 to -0.96) | 0 |
| Denmark | 1995 | 2001 | -3.08  (-3.21 to -2.96) | 0 |
| Denmark | 2001 | 2010 | -1.4  (-1.46 to -1.33) | 0 |
| Denmark | 2010 | 2019 | 0.29  (0.22 to 0.36) | 0 |
| Denmark | 2019 | 2021 | 13.27  (12.38 to 14.16) | 0 |
| Djibouti | 1990 | 2018 | -0.06  (-0.11 to -0.01) | 0.024 |
| Djibouti | 2018 | 2021 | 6.04  (4.27 to 7.85) | 0 |
| Dominica | 1990 | 1995 | -1.09  (-1.22 to -0.96) | 0 |
| Dominica | 1995 | 2005 | 0.07  (0.01 to 0.12) | 0.022 |
| Dominica | 2005 | 2011 | -0.81  (-0.94 to -0.68) | 0 |
| Dominica | 2011 | 2019 | 1.41  (1.33 to 1.49) | 0 |
| Dominica | 2019 | 2021 | 14.88  (14.09 to 15.68) | 0 |
| Dominican Republic | 1990 | 2004 | 0.44  (0.11 to 0.77) | 0.012 |
| Dominican Republic | 2004 | 2018 | -0.58  (-0.95 to -0.21) | 0.003 |
| Dominican Republic | 2018 | 2021 | 9  (4.45 to 13.74) | 0 |
| Ecuador | 1990 | 2019 | -0.09  (-0.11 to -0.07) | 0 |
| Ecuador | 2019 | 2021 | 23.12  (21.07 to 25.2) | 0 |
| Egypt | 1990 | 2000 | -0.59  (-0.71 to -0.47) | 0 |
| Egypt | 2000 | 2011 | 0  (-0.07 to 0.07) | 0.994 |
| Egypt | 2011 | 2019 | 1.01  (0.79 to 1.22) | 0 |
| Egypt | 2019 | 2021 | 11.6  (9.11 to 14.14) | 0 |
| El Salvador | 1990 | 2018 | -0.76  (-0.87 to -0.65) | 0 |
| El Salvador | 2018 | 2021 | 9.77  (5.68 to 14.01) | 0 |
| Equatorial Guinea | 1990 | 2003 | -0.39  (-0.59 to -0.18) | 0.001 |
| Equatorial Guinea | 2003 | 2018 | 0.07  (-0.11 to 0.25) | 0.443 |
| Equatorial Guinea | 2018 | 2021 | 4.41  (2.16 to 6.7) | 0 |
| Eritrea | 1990 | 1994 | -0.6  (-0.63 to -0.57) | 0 |
| Eritrea | 1994 | 2000 | -0.34  (-0.36 to -0.32) | 0 |
| Eritrea | 2000 | 2015 | -0.2  (-0.2 to -0.2) | 0 |
| Eritrea | 2015 | 2019 | 0.43  (0.38 to 0.48) | 0 |
| Eritrea | 2019 | 2021 | 6.27  (6.14 to 6.4) | 0 |
| Estonia | 1990 | 2001 | -0.49  (-0.54 to -0.44) | 0 |
| Estonia | 2001 | 2013 | -2.65  (-2.7 to -2.6) | 0 |
| Estonia | 2013 | 2019 | -0.34  (-0.54 to -0.13) | 0.002 |
| Estonia | 2019 | 2021 | 18.94  (17.61 to 20.3) | 0 |
| Eswatini | 1990 | 2001 | 0.14  (0.08 to 0.21) | 0 |
| Eswatini | 2001 | 2011 | 0.36  (0.29 to 0.44) | 0 |
| Eswatini | 2011 | 2014 | 1.14  (0.25 to 2.03) | 0.015 |
| Eswatini | 2014 | 2019 | -0.05  (-0.36 to 0.27) | 0.749 |
| Eswatini | 2019 | 2021 | 22.39  (20.88 to 23.91) | 0 |
| Ethiopia | 1990 | 2004 | -0.61  (-0.66 to -0.57) | 0 |
| Ethiopia | 2004 | 2010 | -1.49  (-1.72 to -1.26) | 0 |
| Ethiopia | 2010 | 2019 | 0.5  (0.38 to 0.61) | 0 |
| Ethiopia | 2019 | 2021 | 8.65  (7.44 to 9.86) | 0 |
| Fiji | 1990 | 2005 | 0.04  (0.02 to 0.05) | 0 |
| Fiji | 2005 | 2010 | -0.87  (-0.97 to -0.77) | 0 |
| Fiji | 2010 | 2019 | 0.08  (0.05 to 0.12) | 0 |
| Fiji | 2019 | 2021 | 11.39  (10.96 to 11.81) | 0 |
| Finland | 1990 | 2000 | 0.6  (0.18 to 1.02) | 0.008 |
| Finland | 2000 | 2011 | -1.17  (-1.52 to -0.82) | 0 |
| Finland | 2011 | 2015 | -2.82  (-5.07 to -0.52) | 0.019 |
| Finland | 2015 | 2019 | 0.84  (-1.81 to 3.57) | 0.517 |
| Finland | 2019 | 2021 | 15.27  (6.56 to 24.69) | 0.001 |
| France | 1990 | 1995 | -1.02  (-1.83 to -0.19) | 0.018 |
| France | 1995 | 2000 | 1.4  (0.19 to 2.62) | 0.025 |
| France | 2000 | 2018 | -1.15  (-1.33 to -0.96) | 0 |
| France | 2018 | 2021 | 9.89  (5.86 to 14.08) | 0 |
| Gabon | 1990 | 1993 | -0.34  (-0.92 to 0.26) | 0.248 |
| Gabon | 1993 | 2005 | 0.41  (0.34 to 0.49) | 0 |
| Gabon | 2005 | 2010 | -1.65  (-2 to -1.29) | 0 |
| Gabon | 2010 | 2019 | -0.08  (-0.2 to 0.04) | 0.191 |
| Gabon | 2019 | 2021 | 9.37  (7.9 to 10.87) | 0 |
| Gambia | 1990 | 1994 | 2.26  (2.09 to 2.42) | 0 |
| Gambia | 1994 | 2005 | -0.39  (-0.43 to -0.35) | 0 |
| Gambia | 2005 | 2010 | -2.68  (-2.83 to -2.52) | 0 |
| Gambia | 2010 | 2019 | 0.18  (0.13 to 0.24) | 0 |
| Gambia | 2019 | 2021 | 7.54  (6.87 to 8.2) | 0 |
| Georgia | 1990 | 1995 | -0.57  (-0.65 to -0.49) | 0 |
| Georgia | 1995 | 2006 | -0.28  (-0.31 to -0.26) | 0 |
| Georgia | 2006 | 2012 | 0.02  (-0.06 to 0.09) | 0.691 |
| Georgia | 2012 | 2019 | 0.71  (0.65 to 0.77) | 0 |
| Georgia | 2019 | 2021 | 17.32  (16.82 to 17.82) | 0 |
| Germany | 1990 | 2000 | 0.24  (-0.01 to 0.5) | 0.06 |
| Germany | 2000 | 2004 | 5.1  (3.49 to 6.73) | 0 |
| Germany | 2004 | 2016 | 0.47  (0.22 to 0.72) | 0.001 |
| Germany | 2016 | 2019 | -2.73  (-6.87 to 1.59) | 0.198 |
| Germany | 2019 | 2021 | 11.26  (5.08 to 17.8) | 0.001 |
| Ghana | 1990 | 2003 | 0.26  (0.12 to 0.39) | 0.001 |
| Ghana | 2003 | 2018 | -0.51  (-0.63 to -0.39) | 0 |
| Ghana | 2018 | 2021 | 5.24  (3.59 to 6.91) | 0 |
| Greece | 1990 | 1995 | -0.57  (-2.27 to 1.15) | 0.492 |
| Greece | 1995 | 1999 | 6.62  (2.77 to 10.62) | 0.002 |
| Greece | 1999 | 2011 | -1.06  (-1.5 to -0.63) | 0 |
| Greece | 2011 | 2015 | -5.64  (-8.33 to -2.88) | 0.001 |
| Greece | 2015 | 2021 | 5.82  (4.5 to 7.15) | 0 |
| Greenland | 1990 | 1998 | 0.3  (0.12 to 0.48) | 0.002 |
| Greenland | 1998 | 2019 | -0.83  (-0.88 to -0.79) | 0 |
| Greenland | 2019 | 2021 | 17.76  (15.38 to 20.18) | 0 |
| Grenada | 1990 | 1995 | -0.61  (-0.72 to -0.5) | 0 |
| Grenada | 1995 | 2005 | 0.2  (0.16 to 0.25) | 0 |
| Grenada | 2005 | 2010 | -0.86  (-1 to -0.71) | 0 |
| Grenada | 2010 | 2019 | 0.76  (0.71 to 0.81) | 0 |
| Grenada | 2019 | 2021 | 13.27  (12.67 to 13.88) | 0 |
| Guam | 1990 | 2000 | -0.63  (-0.72 to -0.54) | 0 |
| Guam | 2000 | 2019 | 0.21  (0.17 to 0.25) | 0 |
| Guam | 2019 | 2021 | 14  (12.28 to 15.74) | 0 |
| Guatemala | 1990 | 2004 | 1.06  (0.8 to 1.32) | 0 |
| Guatemala | 2004 | 2018 | -1.37  (-1.65 to -1.08) | 0 |
| Guatemala | 2018 | 2021 | 10.16  (6.37 to 14.09) | 0 |
| Guinea | 1990 | 2018 | -0.03  (-0.07 to 0.01) | 0.177 |
| Guinea | 2018 | 2021 | 4.93  (3.5 to 6.38) | 0 |
| Guinea-Bissau | 1990 | 2018 | 0.03  (-0.03 to 0.08) | 0.311 |
| Guinea-Bissau | 2018 | 2021 | 4.4  (2.61 to 6.23) | 0 |
| Guyana | 1990 | 1995 | 0.31  (0.18 to 0.44) | 0 |
| Guyana | 1995 | 2004 | 1.05  (0.99 to 1.12) | 0 |
| Guyana | 2004 | 2014 | 0.17  (0.12 to 0.22) | 0 |
| Guyana | 2014 | 2019 | -0.97  (-1.16 to -0.79) | 0 |
| Guyana | 2019 | 2021 | 18.71  (17.88 to 19.54) | 0 |
| Haiti | 1990 | 2011 | -0.34  (-0.37 to -0.3) | 0 |
| Haiti | 2011 | 2019 | 0.17  (-0.02 to 0.35) | 0.075 |
| Haiti | 2019 | 2021 | 12.08  (10.24 to 13.95) | 0 |
| Honduras | 1990 | 2019 | 0.09  (0.07 to 0.12) | 0 |
| Honduras | 2019 | 2021 | 19.84  (17.88 to 21.85) | 0 |
| Hungary | 1990 | 1995 | -1  (-1.07 to -0.92) | 0 |
| Hungary | 1995 | 2009 | -1.86  (-1.88 to -1.84) | 0 |
| Hungary | 2009 | 2014 | -1.36  (-1.47 to -1.25) | 0 |
| Hungary | 2014 | 2019 | 0.16  (0.04 to 0.28) | 0.013 |
| Hungary | 2019 | 2021 | 15.43  (14.92 to 15.95) | 0 |
| Iceland | 1990 | 2011 | -0.98  (-1.08 to -0.88) | 0 |
| Iceland | 2011 | 2018 | 0.32  (-0.38 to 1.02) | 0.353 |
| Iceland | 2018 | 2021 | 5.55  (3.05 to 8.11) | 0 |
| India | 1990 | 1994 | 3.33  (2.81 to 3.84) | 0 |
| India | 1994 | 2006 | -0.49  (-0.6 to -0.39) | 0 |
| India | 2006 | 2010 | -7.72  (-8.41 to -7.03) | 0 |
| India | 2010 | 2019 | -0.12  (-0.28 to 0.04) | 0.131 |
| India | 2019 | 2021 | 15.27  (13.45 to 17.12) | 0 |
| Indonesia | 1990 | 1996 | -0.7  (-0.82 to -0.57) | 0 |
| Indonesia | 1996 | 2005 | -0.06  (-0.14 to 0.02) | 0.128 |
| Indonesia | 2005 | 2011 | -0.76  (-0.93 to -0.6) | 0 |
| Indonesia | 2011 | 2019 | 1.26  (1.16 to 1.36) | 0 |
| Indonesia | 2019 | 2021 | 15.21  (14.3 to 16.13) | 0 |
| Iran (Islamic Republic of) | 1990 | 2001 | -0.93  (-1.19 to -0.67) | 0 |
| Iran (Islamic Republic of) | 2001 | 2019 | 1.11  (0.97 to 1.25) | 0 |
| Iran (Islamic Republic of) | 2019 | 2021 | 6.22  (1.94 to 10.68) | 0.006 |
| Iraq | 1990 | 1996 | -0.21  (-2.03 to 1.66) | 0.818 |
| Iraq | 1996 | 1999 | -7.31  (-15.64 to 1.85) | 0.108 |
| Iraq | 1999 | 2010 | -0.42  (-1.08 to 0.25) | 0.204 |
| Iraq | 2010 | 2014 | 6.07  (1.49 to 10.85) | 0.012 |
| Iraq | 2014 | 2021 | 2.1  (0.49 to 3.73) | 0.013 |
| Ireland | 1990 | 2009 | 0.41  (0.36 to 0.46) | 0 |
| Ireland | 2009 | 2015 | -1.32  (-1.68 to -0.97) | 0 |
| Ireland | 2015 | 2019 | 3.01  (1.7 to 4.32) | 0 |
| Ireland | 2019 | 2021 | 15.63  (11.73 to 19.66) | 0 |
| Israel | 1990 | 1994 | 3.35  (3.08 to 3.63) | 0 |
| Israel | 1994 | 2010 | -0.36  (-0.39 to -0.33) | 0 |
| Israel | 2010 | 2015 | -3.08  (-3.32 to -2.83) | 0 |
| Israel | 2015 | 2019 | 1.24  (0.76 to 1.73) | 0 |
| Israel | 2019 | 2021 | 10.47  (9.25 to 11.71) | 0 |
| Italy | 1990 | 1994 | 2.98  (2.59 to 3.38) | 0 |
| Italy | 1994 | 2005 | -0.72  (-0.81 to -0.62) | 0 |
| Italy | 2005 | 2010 | -3.51  (-3.89 to -3.14) | 0 |
| Italy | 2010 | 2019 | 1.54  (1.4 to 1.67) | 0 |
| Italy | 2019 | 2021 | 16.89  (15.45 to 18.34) | 0 |
| Jamaica | 1990 | 1994 | -1.57  (-1.72 to -1.42) | 0 |
| Jamaica | 1994 | 2006 | -0.01  (-0.04 to 0.03) | 0.709 |
| Jamaica | 2006 | 2010 | -1.46  (-1.69 to -1.23) | 0 |
| Jamaica | 2010 | 2019 | 1.39  (1.34 to 1.45) | 0 |
| Jamaica | 2019 | 2021 | 15.7  (15.05 to 16.35) | 0 |
| Japan | 1990 | 1995 | -0.93  (-1.51 to -0.35) | 0.004 |
| Japan | 1995 | 2000 | 4.45  (3.62 to 5.28) | 0 |
| Japan | 2000 | 2006 | 0.69  (0.13 to 1.26) | 0.018 |
| Japan | 2006 | 2019 | -1.01  (-1.15 to -0.86) | 0 |
| Japan | 2019 | 2021 | 14.4  (11.22 to 17.68) | 0 |
| Jordan | 1990 | 2001 | -0.21  (-0.27 to -0.15) | 0 |
| Jordan | 2001 | 2011 | -1.26  (-1.35 to -1.18) | 0 |
| Jordan | 2011 | 2019 | 0.46  (0.33 to 0.59) | 0 |
| Jordan | 2019 | 2021 | 8.03  (6.83 to 9.25) | 0 |
| Kazakhstan | 1990 | 1994 | -1.92  (-3.01 to -0.81) | 0.002 |
| Kazakhstan | 1994 | 2002 | 0.74  (0.29 to 1.19) | 0.003 |
| Kazakhstan | 2002 | 2009 | -0.37  (-0.9 to 0.16) | 0.155 |
| Kazakhstan | 2009 | 2019 | 1.05  (0.72 to 1.37) | 0 |
| Kazakhstan | 2019 | 2021 | 9.85  (4.92 to 15) | 0 |
| Kenya | 1990 | 2002 | 0.25  (-0.06 to 0.56) | 0.111 |
| Kenya | 2002 | 2017 | -1.11  (-1.35 to -0.87) | 0 |
| Kenya | 2017 | 2021 | 4.81  (3.03 to 6.62) | 0 |
| Kiribati | 1990 | 1999 | -0.86  (-0.92 to -0.81) | 0 |
| Kiribati | 1999 | 2012 | -0.5  (-0.54 to -0.46) | 0 |
| Kiribati | 2012 | 2019 | 0.19  (0.08 to 0.3) | 0.001 |
| Kiribati | 2019 | 2021 | 6.57  (5.74 to 7.4) | 0 |
| Kuwait | 1990 | 2001 | 0.16  (-0.21 to 0.52) | 0.386 |
| Kuwait | 2001 | 2016 | -0.58  (-0.83 to -0.34) | 0 |
| Kuwait | 2016 | 2021 | 1.98  (0.62 to 3.35) | 0.006 |
| Kyrgyzstan | 1990 | 2019 | -0.25  (-0.27 to -0.22) | 0 |
| Kyrgyzstan | 2019 | 2021 | 17.78  (15.2 to 20.41) | 0 |
| Lao People's Democratic Republic | 1990 | 1996 | -0.59  (-1.68 to 0.51) | 0.271 |
| Lao People's Democratic Republic | 1996 | 1999 | -5.38  (-10.86 to 0.44) | 0.068 |
| Lao People's Democratic Republic | 1999 | 2010 | -0.8  (-1.22 to -0.36) | 0.001 |
| Lao People's Democratic Republic | 2010 | 2019 | 1.06  (0.44 to 1.69) | 0.002 |
| Lao People's Democratic Republic | 2019 | 2021 | 7.18  (-0.51 to 15.47) | 0.066 |
| Latvia | 1990 | 2006 | -0.76  (-0.84 to -0.68) | 0 |
| Latvia | 2006 | 2011 | -1.68  (-2.25 to -1.1) | 0 |
| Latvia | 2011 | 2019 | 0.2  (-0.06 to 0.47) | 0.125 |
| Latvia | 2019 | 2021 | 17.97  (14.45 to 21.59) | 0 |
| Lebanon | 1990 | 1998 | 2.46  (2.31 to 2.6) | 0 |
| Lebanon | 1998 | 2004 | 0.42  (0.19 to 0.64) | 0.001 |
| Lebanon | 2004 | 2015 | -0.31  (-0.38 to -0.24) | 0 |
| Lebanon | 2015 | 2019 | -2.32  (-2.94 to -1.69) | 0 |
| Lebanon | 2019 | 2021 | 21.19  (19.17 to 23.25) | 0 |
| Lesotho | 1990 | 1994 | -3.56  (-3.74 to -3.37) | 0 |
| Lesotho | 1994 | 2010 | 0.46  (0.44 to 0.49) | 0 |
| Lesotho | 2010 | 2019 | 0.13  (0.07 to 0.19) | 0 |
| Lesotho | 2019 | 2021 | 18.16  (17.33 to 18.98) | 0 |
| Liberia | 1990 | 2001 | -0.04  (-0.06 to -0.01) | 0.004 |
| Liberia | 2001 | 2004 | 6.09  (5.8 to 6.38) | 0 |
| Liberia | 2004 | 2015 | 0.1  (0.08 to 0.12) | 0 |
| Liberia | 2015 | 2019 | -4.62  (-4.76 to -4.48) | 0 |
| Liberia | 2019 | 2021 | 3.92  (3.47 to 4.36) | 0 |
| Libya | 1990 | 2018 | -0.09  (-0.17 to -0.01) | 0.028 |
| Libya | 2018 | 2021 | 6.51  (3.75 to 9.35) | 0 |
| Lithuania | 1990 | 2000 | 0.31  (0.27 to 0.36) | 0 |
| Lithuania | 2000 | 2008 | -1.15  (-1.23 to -1.07) | 0 |
| Lithuania | 2008 | 2015 | -0.87  (-0.98 to -0.77) | 0 |
| Lithuania | 2015 | 2019 | -0.13  (-0.43 to 0.17) | 0.364 |
| Lithuania | 2019 | 2021 | 24.09  (23.16 to 25.03) | 0 |
| Luxembourg | 1990 | 2013 | -1.41  (-1.47 to -1.34) | 0 |
| Luxembourg | 2013 | 2019 | 0.57  (-0.09 to 1.23) | 0.088 |
| Luxembourg | 2019 | 2021 | 13.58  (9.48 to 17.82) | 0 |
| Madagascar | 1990 | 2014 | -0.32  (-0.34 to -0.29) | 0 |
| Madagascar | 2014 | 2019 | 0.52  (0.16 to 0.88) | 0.007 |
| Madagascar | 2019 | 2021 | 9.67  (8.18 to 11.18) | 0 |
| Malawi | 1990 | 1995 | 0.1  (0.03 to 0.17) | 0.006 |
| Malawi | 1995 | 2004 | -0.66  (-0.69 to -0.63) | 0 |
| Malawi | 2004 | 2010 | -1.2  (-1.25 to -1.14) | 0 |
| Malawi | 2010 | 2019 | 0.15  (0.12 to 0.18) | 0 |
| Malawi | 2019 | 2021 | 10.59  (10.18 to 11.01) | 0 |
| Malaysia | 1990 | 2001 | -1.62  (-2.11 to -1.13) | 0 |
| Malaysia | 2001 | 2004 | 8.55  (2.01 to 15.51) | 0.013 |
| Malaysia | 2004 | 2011 | 0  (-0.99 to 1) | 0.997 |
| Malaysia | 2011 | 2015 | 3.09  (0.14 to 6.12) | 0.041 |
| Malaysia | 2015 | 2021 | -0.81  (-2.08 to 0.48) | 0.204 |
| Maldives | 1990 | 1995 | -0.55  (-0.72 to -0.39) | 0 |
| Maldives | 1995 | 2010 | -2.08  (-2.11 to -2.05) | 0 |
| Maldives | 2010 | 2019 | -0.64  (-0.72 to -0.56) | 0 |
| Maldives | 2019 | 2021 | 12.26  (11.26 to 13.27) | 0 |
| Mali | 1990 | 2010 | -0.59  (-0.62 to -0.56) | 0 |
| Mali | 2010 | 2019 | 0.72  (0.61 to 0.82) | 0 |
| Mali | 2019 | 2021 | 5.19  (3.92 to 6.48) | 0 |
| Malta | 1990 | 2012 | -0.39  (-0.47 to -0.31) | 0 |
| Malta | 2012 | 2019 | 1.84  (1.27 to 2.42) | 0 |
| Malta | 2019 | 2021 | 10.46  (6.32 to 14.77) | 0 |
| Marshall Islands | 1990 | 2006 | 0.03  (0 to 0.06) | 0.027 |
| Marshall Islands | 2006 | 2010 | -1.24  (-1.58 to -0.91) | 0 |
| Marshall Islands | 2010 | 2019 | -0.06  (-0.13 to 0.02) | 0.13 |
| Marshall Islands | 2019 | 2021 | 6.68  (5.79 to 7.57) | 0 |
| Mauritania | 1990 | 2011 | -0.53  (-0.56 to -0.5) | 0 |
| Mauritania | 2011 | 2019 | 0.75  (0.59 to 0.91) | 0 |
| Mauritania | 2019 | 2021 | 6.44  (4.89 to 8.01) | 0 |
| Mauritius | 1990 | 2001 | 0.55  (0.38 to 0.72) | 0 |
| Mauritius | 2001 | 2010 | -1.23  (-1.49 to -0.98) | 0 |
| Mauritius | 2010 | 2015 | -2.37  (-3.13 to -1.6) | 0 |
| Mauritius | 2015 | 2019 | 0.26  (-1.08 to 1.63) | 0.689 |
| Mauritius | 2019 | 2021 | 14.17  (10.53 to 17.92) | 0 |
| Mexico | 1990 | 2005 | 0.35  (0.27 to 0.43) | 0 |
| Mexico | 2005 | 2010 | 5.22  (4.64 to 5.8) | 0 |
| Mexico | 2010 | 2019 | 0.89  (0.7 to 1.08) | 0 |
| Mexico | 2019 | 2021 | 17.42  (15.31 to 19.57) | 0 |
| Micronesia (Federated States of) | 1990 | 1996 | -0.62  (-0.73 to -0.5) | 0 |
| Micronesia (Federated States of) | 1996 | 2005 | -0.21  (-0.29 to -0.14) | 0 |
| Micronesia (Federated States of) | 2005 | 2013 | -0.6  (-0.69 to -0.51) | 0 |
| Micronesia (Federated States of) | 2013 | 2019 | 0.31  (0.16 to 0.46) | 0 |
| Micronesia (Federated States of) | 2019 | 2021 | 6.01  (5.14 to 6.89) | 0 |
| Monaco | 1990 | 2019 | 0.02  (0.01 to 0.03) | 0 |
| Monaco | 2019 | 2021 | 15.09  (14.47 to 15.72) | 0 |
| Mongolia | 1990 | 1996 | 0.19  (-0.07 to 0.45) | 0.142 |
| Mongolia | 1996 | 2019 | -0.41  (-0.44 to -0.37) | 0 |
| Mongolia | 2019 | 2021 | 3.09  (1.24 to 4.98) | 0.002 |
| Montenegro | 1990 | 2003 | 0.08  (0.05 to 0.12) | 0 |
| Montenegro | 2003 | 2010 | -0.72  (-0.83 to -0.61) | 0 |
| Montenegro | 2010 | 2019 | -0.03  (-0.11 to 0.04) | 0.335 |
| Montenegro | 2019 | 2021 | 21.82  (20.8 to 22.85) | 0 |
| Morocco | 1990 | 2018 | -0.29  (-0.4 to -0.17) | 0 |
| Morocco | 2018 | 2021 | 9.3  (5.2 to 13.56) | 0 |
| Mozambique | 1990 | 1994 | -0.23  (-0.3 to -0.16) | 0 |
| Mozambique | 1994 | 2005 | 0.15  (0.13 to 0.16) | 0 |
| Mozambique | 2005 | 2015 | -0.13  (-0.15 to -0.11) | 0 |
| Mozambique | 2015 | 2019 | 0.23  (0.12 to 0.34) | 0 |
| Mozambique | 2019 | 2021 | 10.62  (10.31 to 10.92) | 0 |
| Myanmar | 1990 | 1995 | -0.52  (-1.31 to 0.28) | 0.188 |
| Myanmar | 1995 | 1999 | -3.1  (-4.71 to -1.47) | 0.001 |
| Myanmar | 1999 | 2010 | -0.33  (-0.57 to -0.09) | 0.011 |
| Myanmar | 2010 | 2019 | 2.33  (1.95 to 2.71) | 0 |
| Myanmar | 2019 | 2021 | 16.33  (11.36 to 21.51) | 0 |
| Namibia | 1990 | 1995 | 0.83  (-0.07 to 1.72) | 0.067 |
| Namibia | 1995 | 1999 | -2.56  (-4.27 to -0.82) | 0.006 |
| Namibia | 1999 | 2010 | -0.74  (-0.99 to -0.48) | 0 |
| Namibia | 2010 | 2019 | 1.38  (0.96 to 1.8) | 0 |
| Namibia | 2019 | 2021 | 17.85  (12.15 to 23.83) | 0 |
| Nauru | 1990 | 2000 | -0.05  (-0.11 to 0) | 0.055 |
| Nauru | 2000 | 2004 | 0.39  (0.04 to 0.74) | 0.031 |
| Nauru | 2004 | 2019 | -0.05  (-0.08 to -0.02) | 0.005 |
| Nauru | 2019 | 2021 | 6.9  (6.05 to 7.76) | 0 |
| Nepal | 1990 | 1995 | -0.42  (-1.85 to 1.04) | 0.555 |
| Nepal | 1995 | 2000 | 3  (1.13 to 4.91) | 0.003 |
| Nepal | 2000 | 2010 | 0.51  (0.04 to 0.99) | 0.035 |
| Nepal | 2010 | 2018 | -1.7  (-2.41 to -0.98) | 0 |
| Nepal | 2018 | 2021 | 12.05  (8.06 to 16.18) | 0 |
| Netherlands | 1990 | 2010 | -0.18  (-0.21 to -0.14) | 0 |
| Netherlands | 2010 | 2015 | -2.74  (-3.19 to -2.29) | 0 |
| Netherlands | 2015 | 2019 | 3.15  (2.18 to 4.12) | 0 |
| Netherlands | 2019 | 2021 | 15.95  (13.01 to 18.96) | 0 |
| New Zealand | 1990 | 1993 | 1.5  (0.63 to 2.37) | 0.002 |
| New Zealand | 1993 | 2010 | -0.07  (-0.13 to -0.01) | 0.016 |
| New Zealand | 2010 | 2019 | 0.37  (0.22 to 0.52) | 0 |
| New Zealand | 2019 | 2021 | 2.34  (0.53 to 4.18) | 0.014 |
| Nicaragua | 1990 | 2001 | 0.61  (0.18 to 1.05) | 0.008 |
| Nicaragua | 2001 | 2018 | -0.57  (-0.81 to -0.33) | 0 |
| Nicaragua | 2018 | 2021 | 9.49  (5.68 to 13.44) | 0 |
| Niger | 1990 | 2021 | -0.08  (-0.13 to -0.02) | 0.006 |
| Nigeria | 1990 | 2004 | 0.22  (-0.07 to 0.5) | 0.125 |
| Nigeria | 2004 | 2014 | -2.51  (-3.04 to -1.98) | 0 |
| Nigeria | 2014 | 2021 | 1.05  (0.25 to 1.84) | 0.012 |
| Niue | 1990 | 2006 | 0.06  (0.02 to 0.1) | 0.006 |
| Niue | 2006 | 2019 | -0.07  (-0.13 to 0) | 0.047 |
| Niue | 2019 | 2021 | 7.15  (5.88 to 8.44) | 0 |
| North Macedonia | 1990 | 2012 | -0.7  (-0.74 to -0.66) | 0 |
| North Macedonia | 2012 | 2019 | 0.69  (0.39 to 0.99) | 0 |
| North Macedonia | 2019 | 2021 | 26.38  (23.73 to 29.09) | 0 |
| Northern Mariana Islands | 1990 | 1995 | -0.97  (-1.18 to -0.75) | 0 |
| Northern Mariana Islands | 1995 | 2005 | 0.46  (0.37 to 0.54) | 0 |
| Northern Mariana Islands | 2005 | 2011 | -0.76  (-0.96 to -0.56) | 0 |
| Northern Mariana Islands | 2011 | 2019 | 0.84  (0.72 to 0.96) | 0 |
| Northern Mariana Islands | 2019 | 2021 | 14.24  (12.96 to 15.54) | 0 |
| Norway | 1990 | 1995 | 0.9  (0 to 1.8) | 0.05 |
| Norway | 1995 | 2000 | -1.53  (-2.75 to -0.31) | 0.017 |
| Norway | 2000 | 2004 | 1.68  (-0.31 to 3.7) | 0.093 |
| Norway | 2004 | 2016 | -0.29  (-0.57 to -0.01) | 0.04 |
| Norway | 2016 | 2021 | 4.65  (3.67 to 5.64) | 0 |
| Oman | 1990 | 2012 | -0.23  (-0.25 to -0.21) | 0 |
| Oman | 2012 | 2019 | 1.04  (0.9 to 1.19) | 0 |
| Oman | 2019 | 2021 | 11.59  (10.51 to 12.68) | 0 |
| Pakistan | 1990 | 2006 | 0.47  (0.19 to 0.76) | 0.002 |
| Pakistan | 2006 | 2016 | -1.47  (-2.12 to -0.81) | 0 |
| Pakistan | 2016 | 2021 | 4.59  (2.83 to 6.38) | 0 |
| Palau | 1990 | 2019 | 0.01  (0 to 0.03) | 0.129 |
| Palau | 2019 | 2021 | 6.85  (5.4 to 8.33) | 0 |
| Palestine | 1990 | 1995 | -0.15  (-0.48 to 0.18) | 0.355 |
| Palestine | 1995 | 2000 | 2.12  (1.71 to 2.54) | 0 |
| Palestine | 2000 | 2009 | -0.3  (-0.43 to -0.18) | 0 |
| Palestine | 2009 | 2019 | -1.24  (-1.36 to -1.13) | 0 |
| Palestine | 2019 | 2021 | 12.59  (10.72 to 14.49) | 0 |
| Panama | 1990 | 2018 | -0.06  (-0.2 to 0.07) | 0.346 |
| Panama | 2018 | 2021 | 11.6  (6.58 to 16.85) | 0 |
| Papua New Guinea | 1990 | 2006 | -0.15  (-0.18 to -0.12) | 0 |
| Papua New Guinea | 2006 | 2010 | -0.79  (-1.18 to -0.39) | 0 |
| Papua New Guinea | 2010 | 2019 | -0.17  (-0.26 to -0.09) | 0.001 |
| Papua New Guinea | 2019 | 2021 | 2.86  (1.84 to 3.89) | 0 |
| Paraguay | 1990 | 2019 | 0.22  (0.21 to 0.24) | 0 |
| Paraguay | 2019 | 2021 | 16.97  (15.21 to 18.76) | 0 |
| Peru | 1990 | 2018 | -0.87  (-1.07 to -0.67) | 0 |
| Peru | 2018 | 2021 | 20.29  (11.95 to 29.25) | 0 |
| Philippines | 1990 | 2005 | -1.04  (-1.1 to -0.98) | 0 |
| Philippines | 2005 | 2019 | 0.15  (0.08 to 0.23) | 0 |
| Philippines | 2019 | 2021 | 17.15  (15.36 to 18.97) | 0 |
| Poland | 1990 | 2000 | 0.1  (0.02 to 0.17) | 0.013 |
| Poland | 2000 | 2006 | -3.1  (-3.3 to -2.9) | 0 |
| Poland | 2006 | 2015 | -0.35  (-0.45 to -0.25) | 0 |
| Poland | 2015 | 2019 | 3.94  (3.46 to 4.43) | 0 |
| Poland | 2019 | 2021 | 16.61  (15.47 to 17.77) | 0 |
| Portugal | 1990 | 1995 | -0.85  (-3.08 to 1.43) | 0.444 |
| Portugal | 1995 | 1999 | 3.87  (-1.13 to 9.11) | 0.124 |
| Portugal | 1999 | 2018 | -1.79  (-2.06 to -1.53) | 0 |
| Portugal | 2018 | 2021 | 13.99  (7.25 to 21.16) | 0 |
| Puerto Rico | 1990 | 2012 | -0.41  (-0.5 to -0.32) | 0 |
| Puerto Rico | 2012 | 2019 | 1.53  (0.87 to 2.19) | 0 |
| Puerto Rico | 2019 | 2021 | 12.02  (7.1 to 17.17) | 0 |
| Qatar | 1990 | 2001 | -0.19  (-0.43 to 0.05) | 0.116 |
| Qatar | 2001 | 2010 | -1.14  (-1.52 to -0.75) | 0 |
| Qatar | 2010 | 2018 | 0.09  (-0.41 to 0.6) | 0.702 |
| Qatar | 2018 | 2021 | 5.07  (2.88 to 7.3) | 0 |
| Republic of Korea | 1990 | 2005 | -0.15  (-0.2 to -0.1) | 0 |
| Republic of Korea | 2005 | 2015 | 0.82  (0.73 to 0.91) | 0 |
| Republic of Korea | 2015 | 2019 | -0.24  (-0.85 to 0.38) | 0.433 |
| Republic of Korea | 2019 | 2021 | 9.77  (7.61 to 11.98) | 0 |
| Republic of Moldova | 1990 | 1995 | -0.39  (-0.63 to -0.14) | 0.004 |
| Republic of Moldova | 1995 | 2007 | -1.55  (-1.63 to -1.48) | 0 |
| Republic of Moldova | 2007 | 2019 | -0.14  (-0.22 to -0.06) | 0.001 |
| Republic of Moldova | 2019 | 2021 | 19.21  (17.51 to 20.95) | 0 |
| Romania | 1990 | 2012 | -0.48  (-0.51 to -0.44) | 0 |
| Romania | 2012 | 2019 | 0.57  (0.32 to 0.82) | 0 |
| Romania | 2019 | 2021 | 21.69  (19.56 to 23.87) | 0 |
| Russian Federation | 1990 | 1993 | 0.7  (-1.39 to 2.83) | 0.495 |
| Russian Federation | 1993 | 2010 | -1.1  (-1.26 to -0.94) | 0 |
| Russian Federation | 2010 | 2014 | 3.6  (1.45 to 5.8) | 0.002 |
| Russian Federation | 2014 | 2019 | -0.1  (-1.42 to 1.24) | 0.88 |
| Russian Federation | 2019 | 2021 | 18.23  (13.28 to 23.39) | 0 |
| Rwanda | 1990 | 1995 | 0.93  (0.69 to 1.17) | 0 |
| Rwanda | 1995 | 2003 | -0.97  (-1.1 to -0.84) | 0 |
| Rwanda | 2003 | 2010 | -2.52  (-2.7 to -2.34) | 0 |
| Rwanda | 2010 | 2019 | 0.15  (0.02 to 0.28) | 0.025 |
| Rwanda | 2019 | 2021 | 9.63  (8.16 to 11.12) | 0 |
| Saint Kitts and Nevis | 1990 | 2019 | 0.04  (0.02 to 0.06) | 0 |
| Saint Kitts and Nevis | 2019 | 2021 | 12.62  (11.17 to 14.09) | 0 |
| Saint Lucia | 1990 | 1996 | -0.86  (-0.97 to -0.74) | 0 |
| Saint Lucia | 1996 | 2006 | 0.03  (-0.03 to 0.09) | 0.303 |
| Saint Lucia | 2006 | 2010 | -0.66  (-1.01 to -0.32) | 0.001 |
| Saint Lucia | 2010 | 2019 | 0.95  (0.87 to 1.02) | 0 |
| Saint Lucia | 2019 | 2021 | 18.1  (17.14 to 19.06) | 0 |
| Saint Vincent and the Grenadines | 1990 | 1994 | -1.11  (-1.26 to -0.96) | 0 |
| Saint Vincent and the Grenadines | 1994 | 2005 | 0.08  (0.04 to 0.11) | 0 |
| Saint Vincent and the Grenadines | 2005 | 2010 | -0.64  (-0.79 to -0.49) | 0 |
| Saint Vincent and the Grenadines | 2010 | 2019 | 0.97  (0.92 to 1.03) | 0 |
| Saint Vincent and the Grenadines | 2019 | 2021 | 14.8  (14.17 to 15.44) | 0 |
| Samoa | 1990 | 2000 | -0.55  (-0.6 to -0.49) | 0 |
| Samoa | 2000 | 2005 | -0.06  (-0.28 to 0.16) | 0.565 |
| Samoa | 2005 | 2010 | -1.01  (-1.23 to -0.8) | 0 |
| Samoa | 2010 | 2019 | 0.2  (0.12 to 0.28) | 0 |
| Samoa | 2019 | 2021 | 7.04  (6.12 to 7.96) | 0 |
| San Marino | 1990 | 2019 | 0.06  (0.05 to 0.07) | 0 |
| San Marino | 2019 | 2021 | 18.26  (17.31 to 19.22) | 0 |
| Sao Tome and Principe | 1990 | 2016 | -0.1  (-0.16 to -0.03) | 0.004 |
| Sao Tome and Principe | 2016 | 2021 | 2.75  (1.89 to 3.62) | 0 |
| Saudi Arabia | 1990 | 2018 | 0.13  (0 to 0.25) | 0.045 |
| Saudi Arabia | 2018 | 2021 | 6.66  (2.54 to 10.95) | 0.002 |
| Senegal | 1990 | 2010 | -0.34  (-0.4 to -0.28) | 0 |
| Senegal | 2010 | 2019 | 0.94  (0.71 to 1.18) | 0 |
| Senegal | 2019 | 2021 | 10.55  (7.56 to 13.62) | 0 |
| Serbia | 1990 | 1997 | -0.42  (-0.55 to -0.29) | 0 |
| Serbia | 1997 | 2011 | -1.19  (-1.24 to -1.14) | 0 |
| Serbia | 2011 | 2019 | 0.16  (0.03 to 0.3) | 0.022 |
| Serbia | 2019 | 2021 | 16.64  (15.1 to 18.19) | 0 |
| Seychelles | 1990 | 1999 | -0.55  (-0.61 to -0.5) | 0 |
| Seychelles | 1999 | 2004 | -0.25  (-0.45 to -0.05) | 0.015 |
| Seychelles | 2004 | 2011 | -0.66  (-0.76 to -0.55) | 0 |
| Seychelles | 2011 | 2019 | 1  (0.91 to 1.08) | 0 |
| Seychelles | 2019 | 2021 | 16.22  (15.33 to 17.13) | 0 |
| Sierra Leone | 1990 | 2021 | 0.38  (0.3 to 0.45) | 0 |
| Singapore | 1990 | 2000 | -0.8  (-0.98 to -0.62) | 0 |
| Singapore | 2000 | 2005 | -7.29  (-8.05 to -6.51) | 0 |
| Singapore | 2005 | 2009 | -1.98  (-3.35 to -0.6) | 0.008 |
| Singapore | 2009 | 2018 | -0.37  (-0.66 to -0.08) | 0.015 |
| Singapore | 2018 | 2021 | 4.88  (3.16 to 6.63) | 0 |
| Slovakia | 1990 | 1996 | -0.49  (-0.89 to -0.09) | 0.02 |
| Slovakia | 1996 | 1999 | -2.94  (-4.99 to -0.86) | 0.009 |
| Slovakia | 1999 | 2010 | -0.82  (-0.98 to -0.66) | 0 |
| Slovakia | 2010 | 2019 | 1.15  (0.9 to 1.4) | 0 |
| Slovakia | 2019 | 2021 | 19.09  (15.46 to 22.84) | 0 |
| Slovenia | 1990 | 2001 | -0.69  (-0.8 to -0.58) | 0 |
| Slovenia | 2001 | 2012 | -2.03  (-2.15 to -1.9) | 0 |
| Slovenia | 2012 | 2019 | 0.01  (-0.28 to 0.3) | 0.956 |
| Slovenia | 2019 | 2021 | 15.83  (13.36 to 18.36) | 0 |
| Solomon Islands | 1990 | 2000 | -0.36  (-0.4 to -0.33) | 0 |
| Solomon Islands | 2000 | 2005 | -0.02  (-0.15 to 0.11) | 0.79 |
| Solomon Islands | 2005 | 2011 | -0.78  (-0.87 to -0.69) | 0 |
| Solomon Islands | 2011 | 2019 | -0.08  (-0.14 to -0.03) | 0.005 |
| Solomon Islands | 2019 | 2021 | 6.92  (6.4 to 7.44) | 0 |
| Somalia | 1990 | 2004 | 0.04  (0.01 to 0.07) | 0.025 |
| Somalia | 2004 | 2010 | -0.49  (-0.64 to -0.33) | 0 |
| Somalia | 2010 | 2015 | 0.17  (-0.05 to 0.39) | 0.116 |
| Somalia | 2015 | 2019 | -0.21  (-0.55 to 0.13) | 0.207 |
| Somalia | 2019 | 2021 | 13.69  (12.73 to 14.66) | 0 |
| South Africa | 1990 | 2005 | -0.78  (-0.99 to -0.56) | 0 |
| South Africa | 2005 | 2019 | 1.1  (0.83 to 1.38) | 0 |
| South Africa | 2019 | 2021 | 15.77  (9.37 to 22.55) | 0 |
| South Sudan | 1990 | 2018 | -0.06  (-0.11 to -0.01) | 0.029 |
| South Sudan | 2018 | 2021 | 4.4  (2.5 to 6.33) | 0 |
| Spain | 1990 | 2005 | -1.07  (-1.22 to -0.92) | 0 |
| Spain | 2005 | 2011 | 5.47  (4.49 to 6.46) | 0 |
| Spain | 2011 | 2019 | 1.46  (0.95 to 1.98) | 0 |
| Spain | 2019 | 2021 | 13.6  (6.73 to 20.91) | 0 |
| Sri Lanka | 1990 | 1996 | -0.6  (-0.96 to -0.24) | 0.002 |
| Sri Lanka | 1996 | 2000 | -4.22  (-5.18 to -3.26) | 0 |
| Sri Lanka | 2000 | 2009 | -2.26  (-2.47 to -2.04) | 0 |
| Sri Lanka | 2009 | 2019 | 0.68  (0.49 to 0.87) | 0 |
| Sri Lanka | 2019 | 2021 | 14.55  (11.47 to 17.7) | 0 |
| Sudan | 1990 | 2018 | -0.44  (-0.53 to -0.35) | 0 |
| Sudan | 2018 | 2021 | 7.4  (4.04 to 10.88) | 0 |
| Suriname | 1990 | 2001 | 0.23  (0.16 to 0.3) | 0 |
| Suriname | 2001 | 2005 | 0.77  (0.27 to 1.27) | 0.005 |
| Suriname | 2005 | 2009 | -0.58  (-1.08 to -0.08) | 0.026 |
| Suriname | 2009 | 2019 | 0.01  (-0.09 to 0.1) | 0.892 |
| Suriname | 2019 | 2021 | 19.84  (18.35 to 21.35) | 0 |
| Sweden | 1990 | 2009 | 0.27  (0.08 to 0.45) | 0.006 |
| Sweden | 2009 | 2018 | -1.18  (-1.86 to -0.5) | 0.002 |
| Sweden | 2018 | 2021 | 8.87  (5.04 to 12.84) | 0 |
| Switzerland | 1990 | 2000 | -0.65  (-0.75 to -0.55) | 0 |
| Switzerland | 2000 | 2005 | -8.79  (-9.15 to -8.44) | 0 |
| Switzerland | 2005 | 2009 | 6.49  (5.9 to 7.08) | 0 |
| Switzerland | 2009 | 2019 | -0.13  (-0.24 to -0.02) | 0.019 |
| Switzerland | 2019 | 2021 | 15.53  (13.59 to 17.5) | 0 |
| Syrian Arab Republic | 1990 | 2012 | -0.3  (-0.34 to -0.27) | 0 |
| Syrian Arab Republic | 2012 | 2019 | 1.17  (0.92 to 1.42) | 0 |
| Syrian Arab Republic | 2019 | 2021 | 10.43  (8.63 to 12.26) | 0 |
| Taiwan (Province of China) | 1990 | 1997 | 0.3  (0.2 to 0.4) | 0 |
| Taiwan (Province of China) | 1997 | 2005 | 1.1  (1.01 to 1.2) | 0 |
| Taiwan (Province of China) | 2005 | 2019 | 0.21  (0.17 to 0.24) | 0 |
| Taiwan (Province of China) | 2019 | 2021 | 3.21  (2.32 to 4.1) | 0 |
| Tajikistan | 1990 | 2007 | -0.68  (-0.72 to -0.65) | 0 |
| Tajikistan | 2007 | 2015 | -0.16  (-0.3 to -0.01) | 0.037 |
| Tajikistan | 2015 | 2019 | 1.02  (0.46 to 1.59) | 0.001 |
| Tajikistan | 2019 | 2021 | 15.07  (13.57 to 16.59) | 0 |
| Thailand | 1990 | 1997 | 0.15  (0.11 to 0.19) | 0 |
| Thailand | 1997 | 2005 | -0.14  (-0.18 to -0.1) | 0 |
| Thailand | 2005 | 2010 | -1.29  (-1.39 to -1.19) | 0 |
| Thailand | 2010 | 2019 | -0.34  (-0.37 to -0.3) | 0 |
| Thailand | 2019 | 2021 | 8.97  (8.52 to 9.42) | 0 |
| Timor-Leste | 1990 | 1996 | -0.65  (-0.77 to -0.54) | 0 |
| Timor-Leste | 1996 | 2000 | -1.93  (-2.22 to -1.64) | 0 |
| Timor-Leste | 2000 | 2009 | -1.44  (-1.5 to -1.38) | 0 |
| Timor-Leste | 2009 | 2019 | 0.1  (0.05 to 0.16) | 0.001 |
| Timor-Leste | 2019 | 2021 | 11.44  (10.56 to 12.32) | 0 |
| Togo | 1990 | 2018 | -0.1  (-0.14 to -0.05) | 0 |
| Togo | 2018 | 2021 | 3.32  (1.66 to 5) | 0 |
| Tokelau | 1990 | 2019 | 0.01  (-0.01 to 0.02) | 0.365 |
| Tokelau | 2019 | 2021 | 6.79  (5.5 to 8.1) | 0 |
| Tonga | 1990 | 1997 | -0.46  (-0.55 to -0.37) | 0 |
| Tonga | 1997 | 2005 | -0.11  (-0.2 to -0.02) | 0.017 |
| Tonga | 2005 | 2011 | -0.73  (-0.87 to -0.58) | 0 |
| Tonga | 2011 | 2019 | 0.35  (0.26 to 0.43) | 0 |
| Tonga | 2019 | 2021 | 6.75  (5.88 to 7.63) | 0 |
| Trinidad and Tobago | 1990 | 2008 | -0.81  (-0.85 to -0.76) | 0 |
| Trinidad and Tobago | 2008 | 2019 | 0.17  (0.07 to 0.27) | 0.002 |
| Trinidad and Tobago | 2019 | 2021 | 19.9  (17.9 to 21.94) | 0 |
| Tunisia | 1990 | 2002 | -0.66  (-0.71 to -0.61) | 0 |
| Tunisia | 2002 | 2011 | -0.17  (-0.25 to -0.08) | 0 |
| Tunisia | 2011 | 2019 | 0.71  (0.59 to 0.83) | 0 |
| Tunisia | 2019 | 2021 | 18.21  (16.97 to 19.45) | 0 |
| Turkey | 1990 | 1998 | 0.15  (-0.05 to 0.35) | 0.142 |
| Turkey | 1998 | 2005 | 1.74  (1.4 to 2.09) | 0 |
| Turkey | 2005 | 2009 | -1.79  (-2.97 to -0.59) | 0.006 |
| Turkey | 2009 | 2019 | -0.61  (-0.85 to -0.37) | 0 |
| Turkey | 2019 | 2021 | 11.51  (7.43 to 15.74) | 0 |
| Turkmenistan | 1990 | 2013 | -0.45  (-0.46 to -0.43) | 0 |
| Turkmenistan | 2013 | 2019 | 0.3  (0.16 to 0.44) | 0 |
| Turkmenistan | 2019 | 2021 | 13.58  (12.7 to 14.47) | 0 |
| Tuvalu | 1990 | 2019 | 0.02  (0 to 0.03) | 0.034 |
| Tuvalu | 2019 | 2021 | 6.41  (5.33 to 7.5) | 0 |
| Uganda | 1990 | 2000 | 1.9  (1.79 to 2) | 0 |
| Uganda | 2000 | 2008 | -0.79  (-0.94 to -0.63) | 0 |
| Uganda | 2008 | 2015 | -3.16  (-3.39 to -2.93) | 0 |
| Uganda | 2015 | 2019 | 0.21  (-0.57 to 1.01) | 0.576 |
| Uganda | 2019 | 2021 | 9.79  (7.7 to 11.92) | 0 |
| Ukraine | 1990 | 1996 | 1.76  (1.37 to 2.15) | 0 |
| Ukraine | 1996 | 2008 | -0.66  (-0.81 to -0.51) | 0 |
| Ukraine | 2008 | 2015 | -2.23  (-2.6 to -1.86) | 0 |
| Ukraine | 2015 | 2019 | 0.06  (-1.06 to 1.2) | 0.912 |
| Ukraine | 2019 | 2021 | 17.76  (14.68 to 20.93) | 0 |
| United Arab Emirates | 1990 | 2001 | -0.21  (-0.24 to -0.17) | 0 |
| United Arab Emirates | 2001 | 2010 | -1.47  (-1.52 to -1.42) | 0 |
| United Arab Emirates | 2010 | 2019 | 0.54  (0.48 to 0.59) | 0 |
| United Arab Emirates | 2019 | 2021 | 11.89  (11.24 to 12.55) | 0 |
| United Kingdom | 1990 | 2009 | -1.23  (-1.4 to -1.06) | 0 |
| United Kingdom | 2009 | 2019 | 1.4  (0.84 to 1.96) | 0 |
| United Kingdom | 2019 | 2021 | 15.58  (8.76 to 22.82) | 0 |
| United Republic of Tanzania | 1990 | 2001 | -0.16  (-0.19 to -0.14) | 0 |
| United Republic of Tanzania | 2001 | 2011 | -0.57  (-0.61 to -0.53) | 0 |
| United Republic of Tanzania | 2011 | 2019 | 0.17  (0.12 to 0.23) | 0 |
| United Republic of Tanzania | 2019 | 2021 | 8.15  (7.61 to 8.69) | 0 |
| United States Virgin Islands | 1990 | 1994 | -0.84  (-1.39 to -0.29) | 0.005 |
| United States Virgin Islands | 1994 | 2011 | -0.04  (-0.1 to 0.03) | 0.26 |
| United States Virgin Islands | 2011 | 2019 | 0.78  (0.55 to 1.01) | 0 |
| United States Virgin Islands | 2019 | 2021 | 13.07  (10.87 to 15.3) | 0 |
| United States of America | 1990 | 1999 | 4.4  (3.72 to 5.08) | 0 |
| United States of America | 1999 | 2019 | -0.41  (-0.61 to -0.2) | 0 |
| United States of America | 2019 | 2021 | 17.52  (9.46 to 26.16) | 0 |
| Uruguay | 1990 | 1994 | -2.27  (-3.33 to -1.2) | 0 |
| Uruguay | 1994 | 2011 | 0.76  (0.64 to 0.87) | 0 |
| Uruguay | 2011 | 2015 | 2.54  (0.86 to 4.26) | 0.005 |
| Uruguay | 2015 | 2019 | -0.82  (-2.52 to 0.92) | 0.334 |
| Uruguay | 2019 | 2021 | 16.66  (11.77 to 21.76) | 0 |
| Uzbekistan | 1990 | 2019 | -0.35  (-0.38 to -0.32) | 0 |
| Uzbekistan | 2019 | 2021 | 12.27  (9.48 to 15.13) | 0 |
| Vanuatu | 1990 | 2006 | -0.32  (-0.35 to -0.29) | 0 |
| Vanuatu | 2006 | 2010 | -1.08  (-1.47 to -0.69) | 0 |
| Vanuatu | 2010 | 2019 | 0.09  (0.01 to 0.18) | 0.038 |
| Vanuatu | 2019 | 2021 | 6.72  (5.65 to 7.8) | 0 |
| Venezuela (Bolivarian Republic of) | 1990 | 2018 | -0.11  (-0.16 to -0.06) | 0 |
| Venezuela (Bolivarian Republic of) | 2018 | 2021 | 5.17  (3.4 to 6.98) | 0 |
| Viet Nam | 1990 | 2003 | 0.1  (-0.06 to 0.26) | 0.216 |
| Viet Nam | 2003 | 2019 | -1.09  (-1.22 to -0.96) | 0 |
| Viet Nam | 2019 | 2021 | 12.92  (8.38 to 17.65) | 0 |
| Yemen | 1990 | 2021 | -0.15  (-0.21 to -0.09) | 0 |
| Zambia | 1990 | 1997 | 0.03  (-0.06 to 0.11) | 0.542 |
| Zambia | 1997 | 2005 | -0.48  (-0.55 to -0.41) | 0 |
| Zambia | 2005 | 2010 | -1.05  (-1.21 to -0.89) | 0 |
| Zambia | 2010 | 2019 | 0.45  (0.38 to 0.51) | 0 |
| Zambia | 2019 | 2021 | 10.02  (9.18 to 10.86) | 0 |
| Zimbabwe | 1990 | 2001 | -0.54  (-0.68 to -0.41) | 0 |
| Zimbabwe | 2001 | 2005 | 0.43  (-0.52 to 1.4) | 0.354 |
| Zimbabwe | 2005 | 2009 | -0.83  (-1.78 to 0.14) | 0.089 |
| Zimbabwe | 2009 | 2019 | 0.81  (0.62 to 0.99) | 0 |
| Zimbabwe | 2019 | 2021 | 10.45  (7.82 to 13.14) | 0 |

Supplementary Table 8. APCs of 204 countries in ASPR of depressive disorders in WCBA. Abbreviations: WCBA, women of childbearing age; APC, annual percentage change; CI, confidence interval; ASPR, age-standardized prevalence rate.

| Location | Year. Start | Year. End | APC (95% CI) | p value |
| --- | --- | --- | --- | --- |
| Afghanistan | 1990 | 2019 | -0.06  (-0.07 to -0.05) | 0 |
| Afghanistan | 2019 | 2021 | 9.71  (8.77 to 10.65) | 0 |
| Albania | 1990 | 2002 | 0.26  (0.2 to 0.33) | 0 |
| Albania | 2002 | 2019 | -0.06  (-0.11 to -0.02) | 0.008 |
| Albania | 2019 | 2021 | 13.57  (12.03 to 15.12) | 0 |
| Algeria | 1990 | 2017 | -0.19  (-0.26 to -0.12) | 0 |
| Algeria | 2017 | 2021 | 3.87  (2.37 to 5.38) | 0 |
| American Samoa | 1990 | 1994 | -0.35  (-0.54 to -0.16) | 0.001 |
| American Samoa | 1994 | 2014 | -0.07  (-0.09 to -0.05) | 0 |
| American Samoa | 2014 | 2019 | 0.36  (0.17 to 0.55) | 0.001 |
| American Samoa | 2019 | 2021 | 3.73  (3.07 to 4.39) | 0 |
| Andorra | 1990 | 2018 | -0.18  (-0.27 to -0.08) | 0.001 |
| Andorra | 2018 | 2021 | 9.34  (5.82 to 12.97) | 0 |
| Angola | 1990 | 2019 | -0.14  (-0.17 to -0.12) | 0 |
| Angola | 2019 | 2021 | 7.05  (4.95 to 9.19) | 0 |
| Antigua and Barbuda | 1990 | 1994 | -1.1  (-1.21 to -0.98) | 0 |
| Antigua and Barbuda | 1994 | 2005 | -0.03  (-0.06 to 0) | 0.036 |
| Antigua and Barbuda | 2005 | 2011 | -0.7  (-0.78 to -0.62) | 0 |
| Antigua and Barbuda | 2011 | 2019 | 1.29  (1.24 to 1.34) | 0 |
| Antigua and Barbuda | 2019 | 2021 | 10.44  (9.99 to 10.89) | 0 |
| Argentina | 1990 | 2006 | 0.08  (0.04 to 0.12) | 0 |
| Argentina | 2006 | 2009 | -3.21  (-4.03 to -2.39) | 0 |
| Argentina | 2009 | 2019 | 0.06  (-0.01 to 0.13) | 0.073 |
| Argentina | 2019 | 2021 | 15.8  (14.61 to 17) | 0 |
| Armenia | 1990 | 2011 | -0.11  (-0.15 to -0.07) | 0 |
| Armenia | 2011 | 2019 | 0.68  (0.48 to 0.88) | 0 |
| Armenia | 2019 | 2021 | 12.98  (11.02 to 14.97) | 0 |
| Australia | 1990 | 2000 | 0.68  (0.62 to 0.74) | 0 |
| Australia | 2000 | 2005 | 1.84  (1.61 to 2.06) | 0 |
| Australia | 2005 | 2019 | -0.89  (-0.93 to -0.84) | 0 |
| Australia | 2019 | 2021 | 5.13  (3.88 to 6.39) | 0 |
| Austria | 1990 | 1995 | -0.26  (-0.59 to 0.06) | 0.105 |
| Austria | 1995 | 1999 | -2.09  (-2.77 to -1.4) | 0 |
| Austria | 1999 | 2011 | -0.88  (-0.98 to -0.79) | 0 |
| Austria | 2011 | 2019 | 0.5  (0.31 to 0.68) | 0 |
| Austria | 2019 | 2021 | 10.44  (8.43 to 12.48) | 0 |
| Azerbaijan | 1990 | 1994 | -0.77  (-0.98 to -0.57) | 0 |
| Azerbaijan | 1994 | 2013 | -0.11  (-0.13 to -0.09) | 0 |
| Azerbaijan | 2013 | 2019 | 1  (0.86 to 1.15) | 0 |
| Azerbaijan | 2019 | 2021 | 11.1  (10.29 to 11.92) | 0 |
| Bahamas | 1990 | 2012 | -0.22  (-0.27 to -0.17) | 0 |
| Bahamas | 2012 | 2019 | 1.16  (0.81 to 1.52) | 0 |
| Bahamas | 2019 | 2021 | 12.54  (9.74 to 15.42) | 0 |
| Bahrain | 1990 | 2000 | 0.09  (0.04 to 0.15) | 0.002 |
| Bahrain | 2000 | 2014 | -0.95  (-0.98 to -0.91) | 0 |
| Bahrain | 2014 | 2019 | 0.34  (0.09 to 0.58) | 0.009 |
| Bahrain | 2019 | 2021 | 9.6  (8.61 to 10.6) | 0 |
| Bangladesh | 1990 | 2019 | -0.2  (-0.21 to -0.18) | 0 |
| Bangladesh | 2019 | 2021 | 9.17  (8.04 to 10.32) | 0 |
| Barbados | 1990 | 1995 | -0.75  (-0.87 to -0.62) | 0 |
| Barbados | 1995 | 2006 | 0.07  (0.03 to 0.12) | 0.002 |
| Barbados | 2006 | 2010 | -0.43  (-0.69 to -0.16) | 0.003 |
| Barbados | 2010 | 2019 | 0.68  (0.62 to 0.74) | 0 |
| Barbados | 2019 | 2021 | 12.59  (11.84 to 13.33) | 0 |
| Belarus | 1990 | 1995 | 0.59  (0.5 to 0.68) | 0 |
| Belarus | 1995 | 2004 | 0.22  (0.18 to 0.26) | 0 |
| Belarus | 2004 | 2019 | -0.4  (-0.41 to -0.38) | 0 |
| Belarus | 2019 | 2021 | 19  (18.42 to 19.58) | 0 |
| Belgium | 1990 | 2001 | -0.11  (-0.41 to 0.2) | 0.477 |
| Belgium | 2001 | 2010 | 1.78  (1.19 to 2.39) | 0 |
| Belgium | 2010 | 2018 | -0.95  (-1.86 to -0.04) | 0.042 |
| Belgium | 2018 | 2021 | 8.04  (3.59 to 12.68) | 0.001 |
| Belize | 1990 | 2019 | 0.12  (0.08 to 0.16) | 0 |
| Belize | 2019 | 2021 | 11.84  (8.66 to 15.12) | 0 |
| Benin | 1990 | 2003 | 0.06  (0.03 to 0.09) | 0 |
| Benin | 2003 | 2012 | -0.12  (-0.18 to -0.07) | 0 |
| Benin | 2012 | 2019 | 0.17  (0.09 to 0.26) | 0 |
| Benin | 2019 | 2021 | 3.58  (2.97 to 4.19) | 0 |
| Bermuda | 1990 | 2002 | -0.66  (-0.68 to -0.63) | 0 |
| Bermuda | 2002 | 2010 | -1.09  (-1.14 to -1.03) | 0 |
| Bermuda | 2010 | 2019 | 0.35  (0.3 to 0.4) | 0 |
| Bermuda | 2019 | 2021 | 11.88  (11.29 to 12.47) | 0 |
| Bhutan | 1990 | 2013 | -0.51  (-0.57 to -0.44) | 0 |
| Bhutan | 2013 | 2021 | 0.86  (0.51 to 1.21) | 0 |
| Bolivia (Plurinational State of) | 1990 | 2019 | -0.35  (-0.38 to -0.33) | 0 |
| Bolivia (Plurinational State of) | 2019 | 2021 | 22.64  (20.13 to 25.21) | 0 |
| Bosnia and Herzegovina | 1990 | 2001 | -0.21  (-0.26 to -0.16) | 0 |
| Bosnia and Herzegovina | 2001 | 2013 | -1.5  (-1.55 to -1.45) | 0 |
| Bosnia and Herzegovina | 2013 | 2019 | 0.19  (0.01 to 0.38) | 0.039 |
| Bosnia and Herzegovina | 2019 | 2021 | 13.4  (12.29 to 14.52) | 0 |
| Botswana | 1990 | 2002 | 0.48  (0.41 to 0.54) | 0 |
| Botswana | 2002 | 2019 | -0.31  (-0.35 to -0.27) | 0 |
| Botswana | 2019 | 2021 | 10.2  (8.76 to 11.66) | 0 |
| Brazil | 1990 | 2005 | 1.25  (1.06 to 1.44) | 0 |
| Brazil | 2005 | 2010 | -4.44  (-5.63 to -3.23) | 0 |
| Brazil | 2010 | 2019 | -0.61  (-1.02 to -0.2) | 0.005 |
| Brazil | 2019 | 2021 | 15.21  (10.06 to 20.6) | 0 |
| Brunei Darussalam | 1990 | 1994 | -1.06  (-1.25 to -0.86) | 0 |
| Brunei Darussalam | 1994 | 2005 | -0.05  (-0.1 to 0) | 0.046 |
| Brunei Darussalam | 2005 | 2011 | -0.68  (-0.81 to -0.54) | 0 |
| Brunei Darussalam | 2011 | 2019 | 1.23  (1.14 to 1.31) | 0 |
| Brunei Darussalam | 2019 | 2021 | 6.76  (5.97 to 7.55) | 0 |
| Bulgaria | 1990 | 1998 | -0.16  (-0.21 to -0.12) | 0 |
| Bulgaria | 1998 | 2003 | -0.63  (-0.76 to -0.5) | 0 |
| Bulgaria | 2003 | 2010 | -1.2  (-1.27 to -1.13) | 0 |
| Bulgaria | 2010 | 2019 | 0.04  (0 to 0.09) | 0.068 |
| Bulgaria | 2019 | 2021 | 17.82  (17.2 to 18.44) | 0 |
| Burkina Faso | 1990 | 1999 | -0.69  (-0.99 to -0.4) | 0 |
| Burkina Faso | 1999 | 2005 | 1.25  (0.55 to 1.94) | 0.001 |
| Burkina Faso | 2005 | 2010 | -0.45  (-1.39 to 0.49) | 0.323 |
| Burkina Faso | 2010 | 2014 | 1.78  (0.22 to 3.36) | 0.027 |
| Burkina Faso | 2014 | 2021 | -0.69  (-1.18 to -0.21) | 0.008 |
| Burundi | 1990 | 1996 | -0.1  (-0.18 to -0.02) | 0.018 |
| Burundi | 1996 | 2011 | -1.03  (-1.05 to -1.01) | 0 |
| Burundi | 2011 | 2019 | 0.01  (-0.05 to 0.07) | 0.76 |
| Burundi | 2019 | 2021 | 7.09  (6.49 to 7.7) | 0 |
| Cabo Verde | 1990 | 2019 | 0.15  (0.12 to 0.18) | 0 |
| Cabo Verde | 2019 | 2021 | 10.12  (7.44 to 12.86) | 0 |
| Cambodia | 1990 | 1996 | 0.12  (0.01 to 0.22) | 0.03 |
| Cambodia | 1996 | 2010 | -0.97  (-1.01 to -0.94) | 0 |
| Cambodia | 2010 | 2019 | -0.25  (-0.32 to -0.19) | 0 |
| Cambodia | 2019 | 2021 | 7.72  (7.02 to 8.42) | 0 |
| Cameroon | 1990 | 2004 | 0.13  (0.12 to 0.14) | 0 |
| Cameroon | 2004 | 2015 | -0.19  (-0.21 to -0.17) | 0 |
| Cameroon | 2015 | 2019 | 0.29  (0.18 to 0.4) | 0 |
| Cameroon | 2019 | 2021 | 3.23  (2.96 to 3.5) | 0 |
| Canada | 1990 | 2000 | 0.99  (0.92 to 1.05) | 0 |
| Canada | 2000 | 2009 | -1.48  (-1.57 to -1.4) | 0 |
| Canada | 2009 | 2019 | 0.73  (0.65 to 0.81) | 0 |
| Canada | 2019 | 2021 | 12.67  (11.29 to 14.07) | 0 |
| Central African Republic | 1990 | 2018 | -0.08  (-0.16 to -0.01) | 0.026 |
| Central African Republic | 2018 | 2021 | 2.95  (0.47 to 5.5) | 0.021 |
| Chad | 1990 | 2004 | 0.27  (0.13 to 0.4) | 0 |
| Chad | 2004 | 2017 | -0.24  (-0.41 to -0.08) | 0.006 |
| Chad | 2017 | 2021 | 1.58  (0.48 to 2.69) | 0.007 |
| Chile | 1990 | 2005 | -0.32  (-0.35 to -0.28) | 0 |
| Chile | 2005 | 2010 | -2.13  (-2.45 to -1.8) | 0 |
| Chile | 2010 | 2019 | 0.19  (0.06 to 0.33) | 0.007 |
| Chile | 2019 | 2021 | 14.18  (12.47 to 15.93) | 0 |
| China | 1990 | 1995 | -0.76  (-1.11 to -0.41) | 0 |
| China | 1995 | 2000 | -2.48  (-2.95 to -2.02) | 0 |
| China | 2000 | 2005 | -0.8  (-1.26 to -0.33) | 0.002 |
| China | 2005 | 2010 | -1.77  (-2.24 to -1.3) | 0 |
| China | 2010 | 2021 | 0.41  (0.3 to 0.51) | 0 |
| Colombia | 1990 | 2002 | 1.2  (0.91 to 1.5) | 0 |
| Colombia | 2002 | 2006 | -0.87  (-2.87 to 1.17) | 0.379 |
| Colombia | 2006 | 2010 | -5.52  (-7.25 to -3.77) | 0 |
| Colombia | 2010 | 2017 | -0.84  (-1.49 to -0.19) | 0.015 |
| Colombia | 2017 | 2021 | 3.71  (2.13 to 5.31) | 0 |
| Comoros | 1990 | 2003 | -0.12  (-0.15 to -0.09) | 0 |
| Comoros | 2003 | 2012 | -0.38  (-0.43 to -0.32) | 0 |
| Comoros | 2012 | 2019 | 0.26  (0.17 to 0.35) | 0 |
| Comoros | 2019 | 2021 | 6.75  (6.09 to 7.42) | 0 |
| Congo | 1990 | 1995 | -0.16  (-0.95 to 0.64) | 0.679 |
| Congo | 1995 | 2000 | 1.61  (0.59 to 2.65) | 0.004 |
| Congo | 2000 | 2008 | -0.39  (-0.78 to 0) | 0.05 |
| Congo | 2008 | 2018 | -1.14  (-1.43 to -0.85) | 0 |
| Congo | 2018 | 2021 | 5.02  (3.02 to 7.06) | 0 |
| Cook Islands | 1990 | 2019 | 0.01  (0 to 0.02) | 0.216 |
| Cook Islands | 2019 | 2021 | 4.16  (3.4 to 4.93) | 0 |
| Costa Rica | 1990 | 2019 | 0.2  (0.18 to 0.22) | 0 |
| Costa Rica | 2019 | 2021 | 11.78  (9.85 to 13.75) | 0 |
| Coted'Ivoire | 1990 | 2012 | -0.19  (-0.23 to -0.15) | 0 |
| Coted'Ivoire | 2012 | 2021 | 0.66  (0.49 to 0.83) | 0 |
| Croatia | 1990 | 1995 | -0.22  (-0.32 to -0.13) | 0 |
| Croatia | 1995 | 2009 | -1.23  (-1.25 to -1.21) | 0 |
| Croatia | 2009 | 2019 | -0.01  (-0.05 to 0.03) | 0.466 |
| Croatia | 2019 | 2021 | 10.73  (10.16 to 11.3) | 0 |
| Cuba | 1990 | 1996 | -1.4  (-1.53 to -1.26) | 0 |
| Cuba | 1996 | 2009 | -2.74  (-2.79 to -2.69) | 0 |
| Cuba | 2009 | 2012 | -0.71  (-1.56 to 0.15) | 0.099 |
| Cuba | 2012 | 2019 | 0.08  (-0.06 to 0.22) | 0.254 |
| Cuba | 2019 | 2021 | 12.45  (11.25 to 13.65) | 0 |
| Cyprus | 1990 | 1994 | -0.94  (-1.14 to -0.75) | 0 |
| Cyprus | 1994 | 2005 | -0.05  (-0.1 to 0) | 0.057 |
| Cyprus | 2005 | 2011 | -0.65  (-0.79 to -0.51) | 0 |
| Cyprus | 2011 | 2019 | 1.11  (1.03 to 1.2) | 0 |
| Cyprus | 2019 | 2021 | 8.14  (7.34 to 8.95) | 0 |
| Czechia | 1990 | 1995 | 0  (-0.11 to 0.11) | 0.996 |
| Czechia | 1995 | 2003 | -1.16  (-1.22 to -1.1) | 0 |
| Czechia | 2003 | 2014 | -0.7  (-0.74 to -0.66) | 0 |
| Czechia | 2014 | 2019 | 0.34  (0.17 to 0.51) | 0 |
| Czechia | 2019 | 2021 | 11.33  (10.64 to 12.03) | 0 |
| Democratic People's Republic of Korea | 1990 | 1995 | -0.16  (-0.18 to -0.13) | 0 |
| Democratic People's Republic of Korea | 1995 | 2005 | -0.47  (-0.48 to -0.45) | 0 |
| Democratic People's Republic of Korea | 2005 | 2010 | -0.58  (-0.62 to -0.54) | 0 |
| Democratic People's Republic of Korea | 2010 | 2019 | -0.02  (-0.03 to -0.01) | 0.007 |
| Democratic People's Republic of Korea | 2019 | 2021 | 2.15  (2.02 to 2.29) | 0 |
| Democratic Republic of the Congo | 1990 | 2018 | -0.14  (-0.19 to -0.08) | 0 |
| Democratic Republic of the Congo | 2018 | 2021 | 3.19  (1.31 to 5.11) | 0.002 |
| Denmark | 1990 | 1995 | -0.91  (-1.03 to -0.8) | 0 |
| Denmark | 1995 | 2001 | -2.46  (-2.57 to -2.35) | 0 |
| Denmark | 2001 | 2010 | -1.05  (-1.11 to -1) | 0 |
| Denmark | 2010 | 2019 | 0.22  (0.16 to 0.27) | 0 |
| Denmark | 2019 | 2021 | 10.04  (9.33 to 10.75) | 0 |
| Djibouti | 1990 | 2018 | -0.04  (-0.08 to -0.01) | 0.022 |
| Djibouti | 2018 | 2021 | 4.03  (2.87 to 5.22) | 0 |
| Dominica | 1990 | 1995 | -0.81  (-0.92 to -0.71) | 0 |
| Dominica | 1995 | 2005 | 0.05  (0 to 0.09) | 0.032 |
| Dominica | 2005 | 2011 | -0.6  (-0.7 to -0.5) | 0 |
| Dominica | 2011 | 2019 | 1.04  (0.98 to 1.1) | 0 |
| Dominica | 2019 | 2021 | 11.26  (10.66 to 11.87) | 0 |
| Dominican Republic | 1990 | 2004 | 0.33  (0.07 to 0.6) | 0.016 |
| Dominican Republic | 2004 | 2018 | -0.45  (-0.74 to -0.16) | 0.004 |
| Dominican Republic | 2018 | 2021 | 7.09  (3.54 to 10.77) | 0 |
| Ecuador | 1990 | 2019 | -0.08  (-0.09 to -0.06) | 0 |
| Ecuador | 2019 | 2021 | 17.67  (16.09 to 19.27) | 0 |
| Egypt | 1990 | 2000 | -0.41  (-0.49 to -0.34) | 0 |
| Egypt | 2000 | 2011 | 0.01  (-0.04 to 0.06) | 0.715 |
| Egypt | 2011 | 2019 | 0.71  (0.57 to 0.85) | 0 |
| Egypt | 2019 | 2021 | 8.92  (7.33 to 10.54) | 0 |
| El Salvador | 1990 | 2018 | -0.64  (-0.73 to -0.55) | 0 |
| El Salvador | 2018 | 2021 | 7.96  (4.64 to 11.38) | 0 |
| Equatorial Guinea | 1990 | 2003 | -0.31  (-0.47 to -0.14) | 0.001 |
| Equatorial Guinea | 2003 | 2018 | 0.05  (-0.1 to 0.19) | 0.511 |
| Equatorial Guinea | 2018 | 2021 | 3.48  (1.71 to 5.27) | 0 |
| Eritrea | 1990 | 1994 | -0.42  (-0.45 to -0.39) | 0 |
| Eritrea | 1994 | 2000 | -0.24  (-0.26 to -0.22) | 0 |
| Eritrea | 2000 | 2015 | -0.14  (-0.14 to -0.13) | 0 |
| Eritrea | 2015 | 2019 | 0.29  (0.25 to 0.34) | 0 |
| Eritrea | 2019 | 2021 | 4.2  (4.09 to 4.32) | 0 |
| Estonia | 1990 | 2000 | -0.26  (-0.31 to -0.21) | 0 |
| Estonia | 2000 | 2013 | -1.94  (-1.98 to -1.91) | 0 |
| Estonia | 2013 | 2019 | -0.28  (-0.44 to -0.12) | 0.002 |
| Estonia | 2019 | 2021 | 13.54  (12.55 to 14.53) | 0 |
| Eswatini | 1990 | 2001 | 0.07  (0.02 to 0.12) | 0.006 |
| Eswatini | 2001 | 2011 | 0.26  (0.19 to 0.32) | 0 |
| Eswatini | 2011 | 2015 | 0.74  (0.38 to 1.1) | 0 |
| Eswatini | 2015 | 2019 | -0.2  (-0.59 to 0.19) | 0.291 |
| Eswatini | 2019 | 2021 | 16.1  (14.99 to 17.21) | 0 |
| Ethiopia | 1990 | 2011 | -0.69  (-0.73 to -0.65) | 0 |
| Ethiopia | 2011 | 2019 | 0.39  (0.17 to 0.61) | 0.001 |
| Ethiopia | 2019 | 2021 | 5.48  (3.67 to 7.32) | 0 |
| Fiji | 1990 | 2005 | 0.02  (0.02 to 0.03) | 0 |
| Fiji | 2005 | 2010 | -0.5  (-0.56 to -0.44) | 0 |
| Fiji | 2010 | 2019 | 0.03  (0.01 to 0.05) | 0.002 |
| Fiji | 2019 | 2021 | 6.63  (6.4 to 6.86) | 0 |
| Finland | 1990 | 2001 | 0.52  (0.21 to 0.83) | 0.003 |
| Finland | 2001 | 2005 | -1.96  (-3.82 to -0.07) | 0.043 |
| Finland | 2005 | 2009 | 0.17  (-1.72 to 2.09) | 0.854 |
| Finland | 2009 | 2018 | -1.59  (-2.04 to -1.13) | 0 |
| Finland | 2018 | 2021 | 8.42  (5.31 to 11.63) | 0 |
| France | 1990 | 1995 | -0.83  (-1.52 to -0.15) | 0.02 |
| France | 1995 | 2000 | 1.1  (0.09 to 2.12) | 0.034 |
| France | 2000 | 2018 | -0.95  (-1.1 to -0.8) | 0 |
| France | 2018 | 2021 | 7.97  (4.76 to 11.28) | 0 |
| Gabon | 1990 | 1993 | -0.25  (-0.72 to 0.22) | 0.275 |
| Gabon | 1993 | 2005 | 0.33  (0.26 to 0.39) | 0 |
| Gabon | 2005 | 2010 | -1.3  (-1.58 to -1.01) | 0 |
| Gabon | 2010 | 2019 | -0.07  (-0.17 to 0.03) | 0.154 |
| Gabon | 2019 | 2021 | 7.18  (6.02 to 8.35) | 0 |
| Gambia | 1990 | 1994 | 1.82  (1.69 to 1.95) | 0 |
| Gambia | 1994 | 2005 | -0.33  (-0.36 to -0.3) | 0 |
| Gambia | 2005 | 2010 | -2.16  (-2.28 to -2.04) | 0 |
| Gambia | 2010 | 2019 | 0.14  (0.1 to 0.19) | 0 |
| Gambia | 2019 | 2021 | 5.97  (5.44 to 6.5) | 0 |
| Georgia | 1990 | 1994 | -0.43  (-0.5 to -0.35) | 0 |
| Georgia | 1994 | 2004 | -0.2  (-0.22 to -0.18) | 0 |
| Georgia | 2004 | 2011 | -0.07  (-0.11 to -0.03) | 0.001 |
| Georgia | 2011 | 2019 | 0.43  (0.4 to 0.47) | 0 |
| Georgia | 2019 | 2021 | 11.54  (11.22 to 11.85) | 0 |
| Germany | 1990 | 2001 | 0.01  (-0.08 to 0.09) | 0.875 |
| Germany | 2001 | 2004 | 4.18  (2.95 to 5.42) | 0 |
| Germany | 2004 | 2016 | 0.72  (0.62 to 0.81) | 0 |
| Germany | 2016 | 2019 | -2.62  (-4.18 to -1.04) | 0.003 |
| Germany | 2019 | 2021 | 8.94  (6.6 to 11.33) | 0 |
| Ghana | 1990 | 2003 | 0.17  (0.07 to 0.26) | 0.002 |
| Ghana | 2003 | 2018 | -0.35  (-0.43 to -0.26) | 0 |
| Ghana | 2018 | 2021 | 3.61  (2.49 to 4.75) | 0 |
| Greece | 1990 | 1996 | 0.2  (-0.89 to 1.29) | 0.711 |
| Greece | 1996 | 1999 | 6.8  (0.42 to 13.58) | 0.038 |
| Greece | 1999 | 2011 | -0.95  (-1.33 to -0.58) | 0 |
| Greece | 2011 | 2015 | -4.71  (-7.01 to -2.36) | 0.001 |
| Greece | 2015 | 2021 | 4.8  (3.69 to 5.91) | 0 |
| Greenland | 1990 | 1998 | 0.26  (0.11 to 0.42) | 0.002 |
| Greenland | 1998 | 2019 | -0.73  (-0.77 to -0.69) | 0 |
| Greenland | 2019 | 2021 | 15.1  (13.14 to 17.08) | 0 |
| Grenada | 1990 | 1995 | -0.46  (-0.54 to -0.38) | 0 |
| Grenada | 1995 | 2005 | 0.16  (0.13 to 0.19) | 0 |
| Grenada | 2005 | 2010 | -0.64  (-0.75 to -0.54) | 0 |
| Grenada | 2010 | 2019 | 0.56  (0.52 to 0.59) | 0 |
| Grenada | 2019 | 2021 | 10.1  (9.65 to 10.55) | 0 |
| Guam | 1990 | 2000 | -0.36  (-0.41 to -0.31) | 0 |
| Guam | 2000 | 2019 | 0.12  (0.1 to 0.14) | 0 |
| Guam | 2019 | 2021 | 8.21  (7.39 to 9.03) | 0 |
| Guatemala | 1990 | 2004 | 0.87  (0.66 to 1.08) | 0 |
| Guatemala | 2004 | 2018 | -1.12  (-1.35 to -0.89) | 0 |
| Guatemala | 2018 | 2021 | 8.29  (5.24 to 11.42) | 0 |
| Guinea | 1990 | 2018 | -0.02  (-0.05 to 0.01) | 0.126 |
| Guinea | 2018 | 2021 | 3.45  (2.46 to 4.45) | 0 |
| Guinea-Bissau | 1990 | 2018 | 0.02  (-0.02 to 0.05) | 0.372 |
| Guinea-Bissau | 2018 | 2021 | 3.12  (1.86 to 4.4) | 0 |
| Guyana | 1990 | 1995 | 0.29  (0.19 to 0.39) | 0 |
| Guyana | 1995 | 2005 | 0.88  (0.84 to 0.92) | 0 |
| Guyana | 2005 | 2015 | 0.06  (0.01 to 0.1) | 0.01 |
| Guyana | 2015 | 2019 | -1.07  (-1.29 to -0.84) | 0 |
| Guyana | 2019 | 2021 | 16  (15.35 to 16.66) | 0 |
| Haiti | 1990 | 2011 | -0.27  (-0.3 to -0.24) | 0 |
| Haiti | 2011 | 2019 | 0.12  (-0.02 to 0.26) | 0.1 |
| Haiti | 2019 | 2021 | 9.63  (8.24 to 11.04) | 0 |
| Honduras | 1990 | 2019 | 0.07  (0.05 to 0.08) | 0 |
| Honduras | 2019 | 2021 | 15.49  (13.94 to 17.05) | 0 |
| Hungary | 1990 | 1995 | -0.73  (-0.79 to -0.68) | 0 |
| Hungary | 1995 | 2008 | -1.26  (-1.28 to -1.25) | 0 |
| Hungary | 2008 | 2014 | -0.94  (-1 to -0.88) | 0 |
| Hungary | 2014 | 2019 | 0.1  (0.01 to 0.18) | 0.036 |
| Hungary | 2019 | 2021 | 9.81  (9.46 to 10.16) | 0 |
| Iceland | 1990 | 2011 | -0.72  (-0.8 to -0.65) | 0 |
| Iceland | 2011 | 2018 | 0.26  (-0.24 to 0.77) | 0.293 |
| Iceland | 2018 | 2021 | 3.96  (2.22 to 5.74) | 0 |
| India | 1990 | 1994 | 2.42  (2 to 2.84) | 0 |
| India | 1994 | 2006 | -0.36  (-0.44 to -0.27) | 0 |
| India | 2006 | 2010 | -5.5  (-6.07 to -4.93) | 0 |
| India | 2010 | 2019 | -0.05  (-0.18 to 0.08) | 0.435 |
| India | 2019 | 2021 | 10.6  (9.17 to 12.06) | 0 |
| Indonesia | 1990 | 1996 | -0.36  (-0.42 to -0.29) | 0 |
| Indonesia | 1996 | 2005 | -0.03  (-0.07 to 0.01) | 0.171 |
| Indonesia | 2005 | 2011 | -0.38  (-0.47 to -0.29) | 0 |
| Indonesia | 2011 | 2019 | 0.63  (0.57 to 0.68) | 0 |
| Indonesia | 2019 | 2021 | 8.08  (7.64 to 8.53) | 0 |
| Iran (Islamic Republic of) | 1990 | 2002 | -0.8  (-0.93 to -0.67) | 0 |
| Iran (Islamic Republic of) | 2002 | 2015 | 1.29  (1.16 to 1.42) | 0 |
| Iran (Islamic Republic of) | 2015 | 2019 | -0.43  (-1.6 to 0.75) | 0.457 |
| Iran (Islamic Republic of) | 2019 | 2021 | 6.8  (4.27 to 9.39) | 0 |
| Iraq | 1990 | 1996 | -0.18  (-1.62 to 1.28) | 0.794 |
| Iraq | 1996 | 1999 | -5.44  (-12.2 to 1.85) | 0.131 |
| Iraq | 1999 | 2010 | -0.29  (-0.82 to 0.24) | 0.262 |
| Iraq | 2010 | 2014 | 4.38  (0.76 to 8.14) | 0.02 |
| Iraq | 2014 | 2021 | 1.58  (0.34 to 2.83) | 0.015 |
| Ireland | 1990 | 2008 | 0.38  (0.34 to 0.43) | 0 |
| Ireland | 2008 | 2015 | -0.88  (-1.1 to -0.65) | 0 |
| Ireland | 2015 | 2019 | 2.26  (1.22 to 3.3) | 0 |
| Ireland | 2019 | 2021 | 12.89  (9.8 to 16.07) | 0 |
| Israel | 1990 | 1994 | 2.66  (2.43 to 2.89) | 0 |
| Israel | 1994 | 2010 | -0.3  (-0.32 to -0.27) | 0 |
| Israel | 2010 | 2015 | -2.41  (-2.62 to -2.21) | 0 |
| Israel | 2015 | 2019 | 0.98  (0.58 to 1.37) | 0 |
| Israel | 2019 | 2021 | 8.18  (7.2 to 9.17) | 0 |
| Italy | 1990 | 1994 | 2.31  (2 to 2.62) | 0 |
| Italy | 1994 | 2005 | -0.56  (-0.63 to -0.49) | 0 |
| Italy | 2005 | 2010 | -2.71  (-3 to -2.42) | 0 |
| Italy | 2010 | 2019 | 1.19  (1.09 to 1.29) | 0 |
| Italy | 2019 | 2021 | 13.25  (12.14 to 14.37) | 0 |
| Jamaica | 1990 | 1994 | -1.18  (-1.3 to -1.06) | 0 |
| Jamaica | 1994 | 2006 | -0.01  (-0.03 to 0.02) | 0.632 |
| Jamaica | 2006 | 2010 | -1.07  (-1.25 to -0.89) | 0 |
| Jamaica | 2010 | 2019 | 1.02  (0.98 to 1.07) | 0 |
| Jamaica | 2019 | 2021 | 11.9  (11.39 to 12.41) | 0 |
| Japan | 1990 | 1995 | -0.69  (-1.16 to -0.22) | 0.007 |
| Japan | 1995 | 2000 | 3.38  (2.69 to 4.06) | 0 |
| Japan | 2000 | 2005 | 0.56  (-0.1 to 1.23) | 0.091 |
| Japan | 2005 | 2019 | -0.95  (-1.06 to -0.84) | 0 |
| Japan | 2019 | 2021 | 12.73  (10.07 to 15.45) | 0 |
| Jordan | 1990 | 2001 | -0.16  (-0.21 to -0.11) | 0 |
| Jordan | 2001 | 2011 | -0.99  (-1.05 to -0.92) | 0 |
| Jordan | 2011 | 2019 | 0.36  (0.26 to 0.46) | 0 |
| Jordan | 2019 | 2021 | 6.2  (5.27 to 7.14) | 0 |
| Kazakhstan | 1990 | 1994 | -1.36  (-2.13 to -0.59) | 0.002 |
| Kazakhstan | 1994 | 2002 | 0.51  (0.2 to 0.82) | 0.003 |
| Kazakhstan | 2002 | 2009 | -0.24  (-0.61 to 0.14) | 0.203 |
| Kazakhstan | 2009 | 2019 | 0.73  (0.51 to 0.96) | 0 |
| Kazakhstan | 2019 | 2021 | 6.89  (3.62 to 10.27) | 0 |
| Kenya | 1990 | 2002 | 0.16  (-0.05 to 0.37) | 0.128 |
| Kenya | 2002 | 2017 | -0.74  (-0.91 to -0.58) | 0 |
| Kenya | 2017 | 2021 | 3.19  (2.01 to 4.39) | 0 |
| Kiribati | 1990 | 1999 | -0.51  (-0.54 to -0.48) | 0 |
| Kiribati | 1999 | 2012 | -0.28  (-0.3 to -0.26) | 0 |
| Kiribati | 2012 | 2019 | 0.1  (0.05 to 0.16) | 0.001 |
| Kiribati | 2019 | 2021 | 3.71  (3.33 to 4.08) | 0 |
| Kuwait | 1990 | 2001 | 0.12  (-0.16 to 0.39) | 0.389 |
| Kuwait | 2001 | 2016 | -0.45  (-0.64 to -0.26) | 0 |
| Kuwait | 2016 | 2021 | 1.51  (0.47 to 2.56) | 0.006 |
| Kyrgyzstan | 1990 | 2019 | -0.17  (-0.19 to -0.15) | 0 |
| Kyrgyzstan | 2019 | 2021 | 12.2  (10.52 to 13.9) | 0 |
| Lao People's Democratic Republic | 1990 | 1996 | -0.33  (-0.84 to 0.18) | 0.185 |
| Lao People's Democratic Republic | 1996 | 1999 | -3.02  (-5.84 to -0.12) | 0.042 |
| Lao People's Democratic Republic | 1999 | 2010 | -0.37  (-0.6 to -0.14) | 0.003 |
| Lao People's Democratic Republic | 2010 | 2019 | 0.52  (0.2 to 0.85) | 0.003 |
| Lao People's Democratic Republic | 2019 | 2021 | 3.85  (0.45 to 7.36) | 0.028 |
| Latvia | 1990 | 2006 | -0.57  (-0.64 to -0.51) | 0 |
| Latvia | 2006 | 2011 | -1.22  (-1.67 to -0.76) | 0 |
| Latvia | 2011 | 2019 | 0.13  (-0.08 to 0.34) | 0.204 |
| Latvia | 2019 | 2021 | 12.85  (10.33 to 15.43) | 0 |
| Lebanon | 1990 | 1998 | 1.95  (1.84 to 2.06) | 0 |
| Lebanon | 1998 | 2004 | 0.32  (0.14 to 0.51) | 0.002 |
| Lebanon | 2004 | 2015 | -0.25  (-0.31 to -0.19) | 0 |
| Lebanon | 2015 | 2019 | -1.86  (-2.36 to -1.35) | 0 |
| Lebanon | 2019 | 2021 | 16.84  (15.27 to 18.42) | 0 |
| Lesotho | 1990 | 1994 | -2.81  (-2.95 to -2.67) | 0 |
| Lesotho | 1994 | 2010 | 0.36  (0.34 to 0.38) | 0 |
| Lesotho | 2010 | 2019 | 0.1  (0.05 to 0.15) | 0 |
| Lesotho | 2019 | 2021 | 14.08  (13.46 to 14.71) | 0 |
| Liberia | 1990 | 2001 | -0.03  (-0.05 to -0.01) | 0.001 |
| Liberia | 2001 | 2004 | 4.77  (4.56 to 4.97) | 0 |
| Liberia | 2004 | 2015 | 0.07  (0.06 to 0.08) | 0 |
| Liberia | 2015 | 2019 | -3.7  (-3.8 to -3.6) | 0 |
| Liberia | 2019 | 2021 | 3.01  (2.71 to 3.31) | 0 |
| Libya | 1990 | 2018 | -0.07  (-0.13 to -0.01) | 0.023 |
| Libya | 2018 | 2021 | 5.03  (2.92 to 7.18) | 0 |
| Lithuania | 1990 | 2000 | 0.24  (0.21 to 0.27) | 0 |
| Lithuania | 2000 | 2008 | -0.89  (-0.94 to -0.84) | 0 |
| Lithuania | 2008 | 2015 | -0.66  (-0.72 to -0.59) | 0 |
| Lithuania | 2015 | 2019 | -0.11  (-0.31 to 0.08) | 0.234 |
| Lithuania | 2019 | 2021 | 17.95  (17.39 to 18.51) | 0 |
| Luxembourg | 1990 | 2012 | -1.1  (-1.15 to -1.05) | 0 |
| Luxembourg | 2012 | 2019 | 0.23  (-0.12 to 0.6) | 0.19 |
| Luxembourg | 2019 | 2021 | 10.21  (7.31 to 13.19) | 0 |
| Madagascar | 1990 | 2014 | -0.21  (-0.23 to -0.19) | 0 |
| Madagascar | 2014 | 2019 | 0.35  (0.1 to 0.61) | 0.008 |
| Madagascar | 2019 | 2021 | 6.46  (5.46 to 7.47) | 0 |
| Malawi | 1990 | 1995 | 0.06  (0.01 to 0.1) | 0.019 |
| Malawi | 1995 | 2004 | -0.43  (-0.45 to -0.41) | 0 |
| Malawi | 2004 | 2010 | -0.76  (-0.8 to -0.72) | 0 |
| Malawi | 2010 | 2019 | 0.11  (0.08 to 0.13) | 0 |
| Malawi | 2019 | 2021 | 6.62  (6.37 to 6.88) | 0 |
| Malaysia | 1990 | 2001 | -0.94  (-1.27 to -0.62) | 0 |
| Malaysia | 2001 | 2004 | 5.74  (1.16 to 10.52) | 0.016 |
| Malaysia | 2004 | 2010 | -0.08  (-1.03 to 0.89) | 0.868 |
| Malaysia | 2010 | 2016 | 1.47  (0.5 to 2.44) | 0.005 |
| Malaysia | 2016 | 2021 | -1.18  (-2.3 to -0.04) | 0.043 |
| Maldives | 1990 | 1995 | -0.42  (-0.52 to -0.31) | 0 |
| Maldives | 1995 | 2009 | -1.39  (-1.41 to -1.37) | 0 |
| Maldives | 2009 | 2015 | -0.63  (-0.74 to -0.52) | 0 |
| Maldives | 2015 | 2019 | -0.11  (-0.35 to 0.14) | 0.382 |
| Maldives | 2019 | 2021 | 7.13  (6.54 to 7.72) | 0 |
| Mali | 1990 | 2010 | -0.38  (-0.39 to -0.36) | 0 |
| Mali | 2010 | 2019 | 0.46  (0.4 to 0.53) | 0 |
| Mali | 2019 | 2021 | 3.25  (2.53 to 3.98) | 0 |
| Malta | 1990 | 2012 | -0.28  (-0.34 to -0.23) | 0 |
| Malta | 2012 | 2019 | 1.33  (0.92 to 1.75) | 0 |
| Malta | 2019 | 2021 | 7.75  (4.73 to 10.87) | 0 |
| Marshall Islands | 1990 | 2006 | 0.02  (0 to 0.03) | 0.035 |
| Marshall Islands | 2006 | 2010 | -0.7  (-0.9 to -0.51) | 0 |
| Marshall Islands | 2010 | 2019 | -0.04  (-0.09 to 0) | 0.054 |
| Marshall Islands | 2019 | 2021 | 3.79  (3.34 to 4.25) | 0 |
| Mauritania | 1990 | 2011 | -0.33  (-0.35 to -0.31) | 0 |
| Mauritania | 2011 | 2019 | 0.47  (0.37 to 0.57) | 0 |
| Mauritania | 2019 | 2021 | 3.95  (3.06 to 4.84) | 0 |
| Mauritius | 1990 | 2001 | 0.36  (0.25 to 0.48) | 0 |
| Mauritius | 2001 | 2010 | -0.93  (-1.11 to -0.76) | 0 |
| Mauritius | 2010 | 2015 | -1.67  (-2.21 to -1.12) | 0 |
| Mauritius | 2015 | 2019 | 0.19  (-0.75 to 1.13) | 0.678 |
| Mauritius | 2019 | 2021 | 10.21  (7.81 to 12.67) | 0 |
| Mexico | 1990 | 2005 | 0.24  (0.18 to 0.3) | 0 |
| Mexico | 2005 | 2010 | 4.15  (3.69 to 4.62) | 0 |
| Mexico | 2010 | 2019 | 0.73  (0.57 to 0.89) | 0 |
| Mexico | 2019 | 2021 | 14.33  (12.57 to 16.11) | 0 |
| Micronesia (Federated States of) | 1990 | 1996 | -0.36  (-0.43 to -0.3) | 0 |
| Micronesia (Federated States of) | 1996 | 2005 | -0.13  (-0.17 to -0.09) | 0 |
| Micronesia (Federated States of) | 2005 | 2013 | -0.33  (-0.38 to -0.28) | 0 |
| Micronesia (Federated States of) | 2013 | 2019 | 0.16  (0.08 to 0.25) | 0.001 |
| Micronesia (Federated States of) | 2019 | 2021 | 3.43  (2.98 to 3.87) | 0 |
| Monaco | 1990 | 2019 | 0.02  (0.01 to 0.02) | 0 |
| Monaco | 2019 | 2021 | 11.99  (11.5 to 12.49) | 0 |
| Mongolia | 1990 | 1995 | 0.24  (-0.02 to 0.51) | 0.07 |
| Mongolia | 1995 | 2019 | -0.31  (-0.33 to -0.28) | 0 |
| Mongolia | 2019 | 2021 | 2.27  (0.93 to 3.64) | 0.002 |
| Montenegro | 1990 | 2003 | 0.05  (0.03 to 0.07) | 0 |
| Montenegro | 2003 | 2010 | -0.48  (-0.55 to -0.4) | 0 |
| Montenegro | 2010 | 2019 | -0.04  (-0.09 to 0.01) | 0.097 |
| Montenegro | 2019 | 2021 | 14.52  (13.91 to 15.13) | 0 |
| Morocco | 1990 | 2018 | -0.23  (-0.32 to -0.14) | 0 |
| Morocco | 2018 | 2021 | 7.5  (4.21 to 10.9) | 0 |
| Mozambique | 1990 | 1994 | -0.16  (-0.21 to -0.1) | 0 |
| Mozambique | 1994 | 2005 | 0.09  (0.08 to 0.11) | 0 |
| Mozambique | 2005 | 2015 | -0.09  (-0.1 to -0.07) | 0 |
| Mozambique | 2015 | 2019 | 0.14  (0.06 to 0.23) | 0.003 |
| Mozambique | 2019 | 2021 | 7.17  (6.94 to 7.39) | 0 |
| Myanmar | 1990 | 1995 | -0.23  (-0.52 to 0.07) | 0.123 |
| Myanmar | 1995 | 1999 | -1.28  (-1.93 to -0.64) | 0.001 |
| Myanmar | 1999 | 2010 | -0.12  (-0.22 to -0.02) | 0.023 |
| Myanmar | 2010 | 2019 | 0.92  (0.77 to 1.07) | 0 |
| Myanmar | 2019 | 2021 | 7.42  (5.92 to 8.95) | 0 |
| Namibia | 1990 | 1995 | 0.54  (0.02 to 1.06) | 0.044 |
| Namibia | 1995 | 1999 | -1.7  (-2.77 to -0.61) | 0.004 |
| Namibia | 1999 | 2010 | -0.44  (-0.61 to -0.28) | 0 |
| Namibia | 2010 | 2019 | 0.84  (0.59 to 1.09) | 0 |
| Namibia | 2019 | 2021 | 11.61  (8.58 to 14.73) | 0 |
| Nauru | 1990 | 2019 | 0.02  (0.01 to 0.03) | 0.002 |
| Nauru | 2019 | 2021 | 4.03  (3.17 to 4.9) | 0 |
| Nepal | 1990 | 1995 | -0.32  (-1.42 to 0.8) | 0.556 |
| Nepal | 1995 | 2000 | 2.26  (0.8 to 3.74) | 0.004 |
| Nepal | 2000 | 2010 | 0.42  (0.04 to 0.8) | 0.032 |
| Nepal | 2010 | 2018 | -1.33  (-1.9 to -0.76) | 0 |
| Nepal | 2018 | 2021 | 9.29  (6.29 to 12.38) | 0 |
| Netherlands | 1990 | 2010 | -0.09  (-0.13 to -0.05) | 0 |
| Netherlands | 2010 | 2015 | -2.08  (-2.6 to -1.56) | 0 |
| Netherlands | 2015 | 2019 | 2.33  (1.27 to 3.4) | 0 |
| Netherlands | 2019 | 2021 | 12.34  (9.17 to 15.6) | 0 |
| New Zealand | 1990 | 1993 | 1.03  (-0.04 to 2.1) | 0.059 |
| New Zealand | 1993 | 2011 | -0.04  (-0.1 to 0.03) | 0.23 |
| New Zealand | 2011 | 2021 | 0.44  (0.29 to 0.59) | 0 |
| Nicaragua | 1990 | 1999 | 0.63  (0.48 to 0.77) | 0 |
| Nicaragua | 1999 | 2019 | -0.34  (-0.39 to -0.3) | 0 |
| Nicaragua | 2019 | 2021 | 12.41  (10.31 to 14.55) | 0 |
| Niger | 1990 | 2021 | -0.05  (-0.09 to -0.01) | 0.008 |
| Nigeria | 1990 | 2004 | 0.16  (-0.02 to 0.35) | 0.085 |
| Nigeria | 2004 | 2014 | -1.65  (-1.99 to -1.3) | 0 |
| Nigeria | 2014 | 2021 | 0.66  (0.15 to 1.17) | 0.013 |
| Niue | 1990 | 2019 | 0  (-0.01 to 0.02) | 0.5 |
| Niue | 2019 | 2021 | 4.16  (3.25 to 5.09) | 0 |
| North Macedonia | 1990 | 2012 | -0.45  (-0.47 to -0.42) | 0 |
| North Macedonia | 2012 | 2019 | 0.44  (0.26 to 0.62) | 0 |
| North Macedonia | 2019 | 2021 | 16.76  (15.28 to 18.26) | 0 |
| Northern Mariana Islands | 1990 | 1995 | -0.5  (-0.61 to -0.38) | 0 |
| Northern Mariana Islands | 1995 | 2005 | 0.24  (0.2 to 0.29) | 0 |
| Northern Mariana Islands | 2005 | 2011 | -0.39  (-0.5 to -0.28) | 0 |
| Northern Mariana Islands | 2011 | 2019 | 0.41  (0.35 to 0.48) | 0 |
| Northern Mariana Islands | 2019 | 2021 | 7.67  (7.07 to 8.27) | 0 |
| Norway | 1990 | 1995 | 0.71  (0.02 to 1.41) | 0.043 |
| Norway | 1995 | 2000 | -1.13  (-2.08 to -0.18) | 0.023 |
| Norway | 2000 | 2004 | 1.14  (-0.4 to 2.7) | 0.138 |
| Norway | 2004 | 2016 | -0.23  (-0.45 to -0.02) | 0.037 |
| Norway | 2016 | 2021 | 3.66  (2.89 to 4.44) | 0 |
| Oman | 1990 | 2012 | -0.18  (-0.19 to -0.16) | 0 |
| Oman | 2012 | 2019 | 0.8  (0.69 to 0.92) | 0 |
| Oman | 2019 | 2021 | 8.86  (8.03 to 9.71) | 0 |
| Pakistan | 1990 | 2006 | 0.34  (0.14 to 0.55) | 0.002 |
| Pakistan | 2006 | 2016 | -1.06  (-1.55 to -0.58) | 0 |
| Pakistan | 2016 | 2021 | 3.33  (2.06 to 4.62) | 0 |
| Palau | 1990 | 2019 | 0.01  (0 to 0.02) | 0.146 |
| Palau | 2019 | 2021 | 4.19  (3.34 to 5.04) | 0 |
| Palestine | 1990 | 1995 | -0.13  (-0.41 to 0.15) | 0.332 |
| Palestine | 1995 | 2000 | 1.77  (1.42 to 2.12) | 0 |
| Palestine | 2000 | 2009 | -0.26  (-0.36 to -0.15) | 0 |
| Palestine | 2009 | 2019 | -1.04  (-1.14 to -0.94) | 0 |
| Palestine | 2019 | 2021 | 10.32  (8.73 to 11.92) | 0 |
| Panama | 1990 | 2019 | 0  (-0.02 to 0.01) | 0.668 |
| Panama | 2019 | 2021 | 14.82  (13.51 to 16.15) | 0 |
| Papua New Guinea | 1990 | 2006 | -0.09  (-0.11 to -0.08) | 0 |
| Papua New Guinea | 2006 | 2010 | -0.48  (-0.73 to -0.23) | 0.001 |
| Papua New Guinea | 2010 | 2019 | -0.11  (-0.16 to -0.05) | 0.001 |
| Papua New Guinea | 2019 | 2021 | 1.71  (1.15 to 2.28) | 0 |
| Paraguay | 1990 | 1997 | 0  (-0.05 to 0.04) | 0.892 |
| Paraguay | 1997 | 2005 | 0.3  (0.26 to 0.34) | 0 |
| Paraguay | 2005 | 2019 | 0.14  (0.12 to 0.15) | 0 |
| Paraguay | 2019 | 2021 | 13.63  (13.17 to 14.08) | 0 |
| Peru | 1990 | 2018 | -0.57  (-0.7 to -0.43) | 0 |
| Peru | 2018 | 2021 | 13.24  (7.97 to 18.78) | 0 |
| Philippines | 1990 | 2005 | -0.6  (-0.64 to -0.57) | 0 |
| Philippines | 2005 | 2019 | 0.11  (0.06 to 0.15) | 0 |
| Philippines | 2019 | 2021 | 9.91  (8.92 to 10.9) | 0 |
| Poland | 1990 | 2000 | 0.04  (0 to 0.08) | 0.03 |
| Poland | 2000 | 2006 | -1.6  (-1.71 to -1.5) | 0 |
| Poland | 2006 | 2015 | -0.17  (-0.22 to -0.12) | 0 |
| Poland | 2015 | 2019 | 1.95  (1.7 to 2.2) | 0 |
| Poland | 2019 | 2021 | 8.99  (8.44 to 9.55) | 0 |
| Portugal | 1990 | 1995 | -0.7  (-2.56 to 1.2) | 0.448 |
| Portugal | 1995 | 1999 | 3.26  (-0.94 to 7.65) | 0.123 |
| Portugal | 1999 | 2018 | -1.52  (-1.74 to -1.3) | 0 |
| Portugal | 2018 | 2021 | 11.42  (5.95 to 17.18) | 0 |
| Puerto Rico | 1990 | 2012 | -0.29  (-0.36 to -0.23) | 0 |
| Puerto Rico | 2012 | 2019 | 1.07  (0.57 to 1.56) | 0 |
| Puerto Rico | 2019 | 2021 | 8.63  (4.9 to 12.5) | 0 |
| Qatar | 1990 | 2001 | -0.16  (-0.34 to 0.03) | 0.098 |
| Qatar | 2001 | 2010 | -0.89  (-1.19 to -0.58) | 0 |
| Qatar | 2010 | 2018 | 0.08  (-0.31 to 0.46) | 0.683 |
| Qatar | 2018 | 2021 | 3.93  (2.26 to 5.62) | 0 |
| Republic of Korea | 1990 | 2005 | -0.14  (-0.18 to -0.1) | 0 |
| Republic of Korea | 2005 | 2015 | 0.59  (0.52 to 0.66) | 0 |
| Republic of Korea | 2015 | 2019 | -0.04  (-0.48 to 0.4) | 0.845 |
| Republic of Korea | 2019 | 2021 | 7.13  (5.75 to 8.52) | 0 |
| Republic of Moldova | 1990 | 1995 | -0.31  (-0.49 to -0.13) | 0.002 |
| Republic of Moldova | 1995 | 2007 | -1.1  (-1.15 to -1.04) | 0 |
| Republic of Moldova | 2007 | 2019 | -0.09  (-0.15 to -0.04) | 0.002 |
| Republic of Moldova | 2019 | 2021 | 13.01  (11.86 to 14.17) | 0 |
| Romania | 1990 | 2012 | -0.3  (-0.32 to -0.28) | 0 |
| Romania | 2012 | 2019 | 0.35  (0.21 to 0.49) | 0 |
| Romania | 2019 | 2021 | 13.73  (12.59 to 14.89) | 0 |
| Russian Federation | 1990 | 1993 | 0.45  (-1.01 to 1.93) | 0.531 |
| Russian Federation | 1993 | 2010 | -0.72  (-0.83 to -0.61) | 0 |
| Russian Federation | 2010 | 2014 | 2.37  (0.91 to 3.86) | 0.003 |
| Russian Federation | 2014 | 2019 | -0.08  (-0.99 to 0.85) | 0.863 |
| Russian Federation | 2019 | 2021 | 12.39  (9 to 15.88) | 0 |
| Rwanda | 1990 | 1995 | 0.74  (0.56 to 0.92) | 0 |
| Rwanda | 1995 | 2003 | -0.74  (-0.84 to -0.64) | 0 |
| Rwanda | 2003 | 2010 | -1.85  (-1.98 to -1.72) | 0 |
| Rwanda | 2010 | 2019 | 0.11  (0.02 to 0.2) | 0.018 |
| Rwanda | 2019 | 2021 | 6.77  (5.75 to 7.81) | 0 |
| Saint Kitts and Nevis | 1990 | 2019 | 0.03  (0.02 to 0.04) | 0 |
| Saint Kitts and Nevis | 2019 | 2021 | 10.11  (8.96 to 11.27) | 0 |
| Saint Lucia | 1990 | 1996 | -0.64  (-0.73 to -0.56) | 0 |
| Saint Lucia | 1996 | 2006 | 0.03  (-0.02 to 0.07) | 0.27 |
| Saint Lucia | 2006 | 2010 | -0.48  (-0.74 to -0.23) | 0.001 |
| Saint Lucia | 2010 | 2019 | 0.7  (0.65 to 0.76) | 0 |
| Saint Lucia | 2019 | 2021 | 13.76  (13.06 to 14.46) | 0 |
| Saint Vincent and the Grenadines | 1990 | 1994 | -0.83  (-0.94 to -0.72) | 0 |
| Saint Vincent and the Grenadines | 1994 | 2005 | 0.06  (0.03 to 0.08) | 0 |
| Saint Vincent and the Grenadines | 2005 | 2010 | -0.47  (-0.57 to -0.36) | 0 |
| Saint Vincent and the Grenadines | 2010 | 2019 | 0.72  (0.68 to 0.76) | 0 |
| Saint Vincent and the Grenadines | 2019 | 2021 | 11.28  (10.83 to 11.74) | 0 |
| Samoa | 1990 | 2000 | -0.31  (-0.34 to -0.28) | 0 |
| Samoa | 2000 | 2005 | -0.04  (-0.17 to 0.09) | 0.515 |
| Samoa | 2005 | 2010 | -0.55  (-0.68 to -0.43) | 0 |
| Samoa | 2010 | 2019 | 0.1  (0.06 to 0.15) | 0 |
| Samoa | 2019 | 2021 | 3.9  (3.44 to 4.36) | 0 |
| San Marino | 1990 | 2019 | 0.04  (0.03 to 0.05) | 0 |
| San Marino | 2019 | 2021 | 14.54  (13.79 to 15.29) | 0 |
| Sao Tome and Principe | 1990 | 2016 | -0.07  (-0.11 to -0.02) | 0.004 |
| Sao Tome and Principe | 2016 | 2021 | 1.84  (1.25 to 2.43) | 0 |
| Saudi Arabia | 1990 | 2018 | 0.11  (0.02 to 0.21) | 0.021 |
| Saudi Arabia | 2018 | 2021 | 4.9  (1.74 to 8.15) | 0.003 |
| Senegal | 1990 | 2010 | -0.22  (-0.26 to -0.19) | 0 |
| Senegal | 2010 | 2019 | 0.62  (0.47 to 0.77) | 0 |
| Senegal | 2019 | 2021 | 6.86  (5.13 to 8.61) | 0 |
| Serbia | 1990 | 1996 | -0.23  (-0.33 to -0.13) | 0 |
| Serbia | 1996 | 2011 | -0.77  (-0.8 to -0.75) | 0 |
| Serbia | 2011 | 2019 | 0.11  (0.03 to 0.19) | 0.011 |
| Serbia | 2019 | 2021 | 10.73  (9.94 to 11.53) | 0 |
| Seychelles | 1990 | 2011 | -0.25  (-0.27 to -0.24) | 0 |
| Seychelles | 2011 | 2019 | 0.47  (0.39 to 0.55) | 0 |
| Seychelles | 2019 | 2021 | 8.74  (7.99 to 9.48) | 0 |
| Sierra Leone | 1990 | 2021 | 0.26  (0.21 to 0.31) | 0 |
| Singapore | 1990 | 2000 | -0.68  (-0.77 to -0.6) | 0 |
| Singapore | 2000 | 2005 | -6.37  (-6.75 to -5.99) | 0 |
| Singapore | 2005 | 2014 | -0.95  (-1.09 to -0.81) | 0 |
| Singapore | 2014 | 2019 | 0.53  (0.09 to 0.97) | 0.021 |
| Singapore | 2019 | 2021 | 5.17  (3.33 to 7.05) | 0 |
| Slovakia | 1990 | 1996 | -0.32  (-0.56 to -0.08) | 0.012 |
| Slovakia | 1996 | 1999 | -2.02  (-3.32 to -0.71) | 0.005 |
| Slovakia | 1999 | 2010 | -0.51  (-0.61 to -0.41) | 0 |
| Slovakia | 2010 | 2019 | 0.74  (0.58 to 0.89) | 0 |
| Slovakia | 2019 | 2021 | 12.4  (10.42 to 14.42) | 0 |
| Slovenia | 1990 | 2001 | -0.53  (-0.61 to -0.44) | 0 |
| Slovenia | 2001 | 2012 | -1.47  (-1.57 to -1.38) | 0 |
| Slovenia | 2012 | 2019 | 0.01  (-0.2 to 0.23) | 0.91 |
| Slovenia | 2019 | 2021 | 10.96  (9.2 to 12.76) | 0 |
| Solomon Islands | 1990 | 2006 | -0.16  (-0.18 to -0.14) | 0 |
| Solomon Islands | 2006 | 2011 | -0.44  (-0.61 to -0.27) | 0 |
| Solomon Islands | 2011 | 2019 | -0.05  (-0.12 to 0.02) | 0.146 |
| Solomon Islands | 2019 | 2021 | 4.05  (3.43 to 4.68) | 0 |
| Somalia | 1990 | 2004 | 0.02  (0 to 0.04) | 0.047 |
| Somalia | 2004 | 2010 | -0.34  (-0.44 to -0.23) | 0 |
| Somalia | 2010 | 2015 | 0.12  (-0.03 to 0.26) | 0.107 |
| Somalia | 2015 | 2019 | -0.14  (-0.37 to 0.08) | 0.202 |
| Somalia | 2019 | 2021 | 9.43  (8.82 to 10.04) | 0 |
| South Africa | 1990 | 2005 | -0.53  (-0.68 to -0.38) | 0 |
| South Africa | 2005 | 2019 | 0.76  (0.57 to 0.95) | 0 |
| South Africa | 2019 | 2021 | 11.37  (6.94 to 15.97) | 0 |
| South Sudan | 1990 | 2018 | -0.04  (-0.08 to 0) | 0.034 |
| South Sudan | 2018 | 2021 | 3  (1.72 to 4.31) | 0 |
| Spain | 1990 | 2005 | -0.92  (-1.03 to -0.82) | 0 |
| Spain | 2005 | 2011 | 4.26  (3.59 to 4.95) | 0 |
| Spain | 2011 | 2019 | 1.24  (0.88 to 1.6) | 0 |
| Spain | 2019 | 2021 | 10.91  (6.16 to 15.88) | 0 |
| Sri Lanka | 1990 | 1996 | -0.39  (-0.59 to -0.2) | 0 |
| Sri Lanka | 1996 | 2000 | -2.74  (-3.27 to -2.21) | 0 |
| Sri Lanka | 2000 | 2009 | -1.32  (-1.44 to -1.2) | 0 |
| Sri Lanka | 2009 | 2019 | 0.39  (0.29 to 0.5) | 0 |
| Sri Lanka | 2019 | 2021 | 8.6  (7.16 to 10.07) | 0 |
| Sudan | 1990 | 2018 | -0.34  (-0.42 to -0.27) | 0 |
| Sudan | 2018 | 2021 | 5.61  (2.98 to 8.3) | 0 |
| Suriname | 1990 | 2000 | 0.18  (0.12 to 0.24) | 0 |
| Suriname | 2000 | 2005 | 0.57  (0.32 to 0.82) | 0 |
| Suriname | 2005 | 2009 | -0.41  (-0.81 to -0.02) | 0.04 |
| Suriname | 2009 | 2019 | -0.03  (-0.1 to 0.05) | 0.464 |
| Suriname | 2019 | 2021 | 16.72  (15.55 to 17.91) | 0 |
| Sweden | 1990 | 2009 | 0.21  (0.07 to 0.36) | 0.006 |
| Sweden | 2009 | 2018 | -0.9  (-1.44 to -0.36) | 0.002 |
| Sweden | 2018 | 2021 | 7.11  (4.06 to 10.26) | 0 |
| Switzerland | 1990 | 2000 | -0.54  (-0.62 to -0.47) | 0 |
| Switzerland | 2000 | 2005 | -6.68  (-6.94 to -6.41) | 0 |
| Switzerland | 2005 | 2009 | 4.83  (4.4 to 5.26) | 0 |
| Switzerland | 2009 | 2019 | -0.11  (-0.19 to -0.03) | 0.01 |
| Switzerland | 2019 | 2021 | 11.38  (10.07 to 12.7) | 0 |
| Syrian Arab Republic | 1990 | 2012 | -0.23  (-0.26 to -0.2) | 0 |
| Syrian Arab Republic | 2012 | 2019 | 0.9  (0.7 to 1.09) | 0 |
| Syrian Arab Republic | 2019 | 2021 | 7.96  (6.58 to 9.35) | 0 |
| Taiwan (Province of China) | 1990 | 1998 | 0.2  (0.17 to 0.22) | 0 |
| Taiwan (Province of China) | 1998 | 2005 | 0.6  (0.56 to 0.64) | 0 |
| Taiwan (Province of China) | 2005 | 2010 | -0.01  (-0.08 to 0.07) | 0.859 |
| Taiwan (Province of China) | 2010 | 2019 | 0.15  (0.13 to 0.18) | 0 |
| Taiwan (Province of China) | 2019 | 2021 | 1.67  (1.42 to 1.93) | 0 |
| Tajikistan | 1990 | 2002 | -0.5  (-0.53 to -0.46) | 0 |
| Tajikistan | 2002 | 2013 | -0.28  (-0.33 to -0.23) | 0 |
| Tajikistan | 2013 | 2019 | 0.45  (0.3 to 0.6) | 0 |
| Tajikistan | 2019 | 2021 | 9.91  (9.06 to 10.76) | 0 |
| Thailand | 1990 | 1997 | 0.07  (0.05 to 0.1) | 0 |
| Thailand | 1997 | 2005 | -0.11  (-0.14 to -0.09) | 0 |
| Thailand | 2005 | 2010 | -0.74  (-0.8 to -0.69) | 0 |
| Thailand | 2010 | 2019 | -0.2  (-0.22 to -0.18) | 0 |
| Thailand | 2019 | 2021 | 5.08  (4.86 to 5.29) | 0 |
| Timor-Leste | 1990 | 1996 | -0.4  (-0.46 to -0.33) | 0 |
| Timor-Leste | 1996 | 2000 | -1.17  (-1.35 to -1) | 0 |
| Timor-Leste | 2000 | 2009 | -0.82  (-0.86 to -0.79) | 0 |
| Timor-Leste | 2009 | 2019 | 0.06  (0.03 to 0.1) | 0.001 |
| Timor-Leste | 2019 | 2021 | 6.39  (5.94 to 6.85) | 0 |
| Togo | 1990 | 2018 | -0.07  (-0.1 to -0.03) | 0 |
| Togo | 2018 | 2021 | 2.3  (1.15 to 3.46) | 0 |
| Tokelau | 1990 | 2019 | 0.01  (0 to 0.02) | 0.29 |
| Tokelau | 2019 | 2021 | 4.14  (3.39 to 4.89) | 0 |
| Tonga | 1990 | 1997 | -0.25  (-0.3 to -0.2) | 0 |
| Tonga | 1997 | 2005 | -0.06  (-0.11 to -0.02) | 0.012 |
| Tonga | 2005 | 2011 | -0.39  (-0.47 to -0.31) | 0 |
| Tonga | 2011 | 2019 | 0.17  (0.13 to 0.22) | 0 |
| Tonga | 2019 | 2021 | 3.72  (3.32 to 4.11) | 0 |
| Trinidad and Tobago | 1990 | 2008 | -0.65  (-0.68 to -0.61) | 0 |
| Trinidad and Tobago | 2008 | 2019 | 0.12  (0.04 to 0.2) | 0.007 |
| Trinidad and Tobago | 2019 | 2021 | 15.89  (14.27 to 17.53) | 0 |
| Tunisia | 1990 | 2003 | -0.51  (-0.55 to -0.48) | 0 |
| Tunisia | 2003 | 2011 | -0.09  (-0.17 to -0.01) | 0.028 |
| Tunisia | 2011 | 2019 | 0.56  (0.47 to 0.65) | 0 |
| Tunisia | 2019 | 2021 | 14.6  (13.62 to 15.58) | 0 |
| Turkey | 1990 | 1998 | 0.11  (-0.06 to 0.28) | 0.194 |
| Turkey | 1998 | 2005 | 1.37  (1.09 to 1.65) | 0 |
| Turkey | 2005 | 2009 | -1.31  (-2.26 to -0.34) | 0.011 |
| Turkey | 2009 | 2019 | -0.48  (-0.67 to -0.29) | 0 |
| Turkey | 2019 | 2021 | 8.99  (5.79 to 12.3) | 0 |
| Turkmenistan | 1990 | 2013 | -0.3  (-0.31 to -0.29) | 0 |
| Turkmenistan | 2013 | 2019 | 0.22  (0.13 to 0.31) | 0 |
| Turkmenistan | 2019 | 2021 | 8.87  (8.35 to 9.39) | 0 |
| Tuvalu | 1990 | 2019 | 0.01  (0 to 0.02) | 0.045 |
| Tuvalu | 2019 | 2021 | 3.9  (3.27 to 4.54) | 0 |
| Uganda | 1990 | 2000 | 1.58  (1.49 to 1.67) | 0 |
| Uganda | 2000 | 2007 | -0.55  (-0.72 to -0.38) | 0 |
| Uganda | 2007 | 2015 | -2.38  (-2.53 to -2.23) | 0 |
| Uganda | 2015 | 2019 | 0.08  (-0.58 to 0.74) | 0.8 |
| Uganda | 2019 | 2021 | 7.37  (5.68 to 9.1) | 0 |
| Ukraine | 1990 | 1996 | 1.31  (1.02 to 1.6) | 0 |
| Ukraine | 1996 | 2008 | -0.5  (-0.61 to -0.39) | 0 |
| Ukraine | 2008 | 2015 | -1.62  (-1.9 to -1.35) | 0 |
| Ukraine | 2015 | 2019 | 0.04  (-0.76 to 0.84) | 0.922 |
| Ukraine | 2019 | 2021 | 13.05  (10.85 to 15.29) | 0 |
| United Arab Emirates | 1990 | 2001 | -0.16  (-0.18 to -0.13) | 0 |
| United Arab Emirates | 2001 | 2010 | -1.11  (-1.15 to -1.07) | 0 |
| United Arab Emirates | 2010 | 2019 | 0.41  (0.37 to 0.45) | 0 |
| United Arab Emirates | 2019 | 2021 | 8.86  (8.36 to 9.36) | 0 |
| United Kingdom | 1990 | 2009 | -0.96  (-1.09 to -0.82) | 0 |
| United Kingdom | 2009 | 2019 | 1.07  (0.65 to 1.49) | 0 |
| United Kingdom | 2019 | 2021 | 12.27  (6.94 to 17.86) | 0 |
| United Republic of Tanzania | 1990 | 2001 | -0.11  (-0.13 to -0.09) | 0 |
| United Republic of Tanzania | 2001 | 2011 | -0.38  (-0.41 to -0.36) | 0 |
| United Republic of Tanzania | 2011 | 2019 | 0.12  (0.08 to 0.15) | 0 |
| United Republic of Tanzania | 2019 | 2021 | 5.44  (5.13 to 5.75) | 0 |
| United States Virgin Islands | 1990 | 1994 | -0.63  (-1.02 to -0.24) | 0.003 |
| United States Virgin Islands | 1994 | 2011 | -0.02  (-0.07 to 0.02) | 0.295 |
| United States Virgin Islands | 2011 | 2019 | 0.59  (0.42 to 0.75) | 0 |
| United States Virgin Islands | 2019 | 2021 | 9.97  (8.38 to 11.57) | 0 |
| United States of America | 1990 | 1997 | 3  (2.67 to 3.33) | 0 |
| United States of America | 1997 | 2019 | -0.14  (-0.2 to -0.08) | 0 |
| United States of America | 2019 | 2021 | 14.32  (11.55 to 17.16) | 0 |
| Uruguay | 1990 | 1994 | -1.85  (-2.69 to -1.01) | 0 |
| Uruguay | 1994 | 2011 | 0.59  (0.5 to 0.69) | 0 |
| Uruguay | 2011 | 2015 | 2.16  (0.83 to 3.51) | 0.003 |
| Uruguay | 2015 | 2019 | -0.67  (-2.05 to 0.73) | 0.325 |
| Uruguay | 2019 | 2021 | 13.69  (9.85 to 17.67) | 0 |
| Uzbekistan | 1990 | 2019 | -0.24  (-0.26 to -0.22) | 0 |
| Uzbekistan | 2019 | 2021 | 8.19  (6.39 to 10.02) | 0 |
| Vanuatu | 1990 | 2006 | -0.19  (-0.21 to -0.17) | 0 |
| Vanuatu | 2006 | 2010 | -0.62  (-0.87 to -0.38) | 0 |
| Vanuatu | 2010 | 2019 | 0.05  (-0.01 to 0.1) | 0.093 |
| Vanuatu | 2019 | 2021 | 3.95  (3.37 to 4.54) | 0 |
| Venezuela (Bolivarian Republic of) | 1990 | 2018 | -0.08  (-0.12 to -0.04) | 0 |
| Venezuela (Bolivarian Republic of) | 2018 | 2021 | 3.99  (2.6 to 5.39) | 0 |
| Viet Nam | 1990 | 2003 | 0.02  (-0.06 to 0.11) | 0.591 |
| Viet Nam | 2003 | 2019 | -0.58  (-0.65 to -0.51) | 0 |
| Viet Nam | 2019 | 2021 | 6.9  (4.94 to 8.89) | 0 |
| Yemen | 1990 | 2021 | -0.13  (-0.18 to -0.08) | 0 |
| Zambia | 1990 | 2001 | -0.06  (-0.1 to -0.02) | 0.004 |
| Zambia | 2001 | 2010 | -0.52  (-0.58 to -0.47) | 0 |
| Zambia | 2010 | 2019 | 0.2  (0.14 to 0.27) | 0 |
| Zambia | 2019 | 2021 | 6.35  (5.63 to 7.06) | 0 |
| Zimbabwe | 1990 | 2001 | -0.33  (-0.4 to -0.25) | 0 |
| Zimbabwe | 2001 | 2005 | 0.26  (-0.31 to 0.83) | 0.352 |
| Zimbabwe | 2005 | 2009 | -0.48  (-1.05 to 0.09) | 0.094 |
| Zimbabwe | 2009 | 2019 | 0.47  (0.36 to 0.58) | 0 |
| Zimbabwe | 2019 | 2021 | 6.17  (4.76 to 7.61) | 0 |

Supplementary Table 9. APCs of 204 countries in ASDR of depressive disorders in WCBA. Abbreviations: WCBA, women of childbearing age; APC, annual percentage change; CI, confidence interval; ASDR, age-standardized DALYs rate.

| Location | Year. Start | Year. End | APC (95% CI) | p value |
| --- | --- | --- | --- | --- |
| Afghanistan | 1990 | 2019 | -0.04  (-0.05 to -0.03) | 0 |
| Afghanistan | 2019 | 2021 | 10.77  (10.01 to 11.53) | 0 |
| Albania | 1990 | 2002 | 0.36  (0.28 to 0.45) | 0 |
| Albania | 2002 | 2019 | -0.07  (-0.13 to -0.02) | 0.011 |
| Albania | 2019 | 2021 | 17.05  (15.14 to 19) | 0 |
| Algeria | 1990 | 2017 | -0.21  (-0.3 to -0.13) | 0 |
| Algeria | 2017 | 2021 | 4.52  (2.75 to 6.33) | 0 |
| American Samoa | 1990 | 1994 | -0.53  (-0.79 to -0.26) | 0 |
| American Samoa | 1994 | 2014 | -0.12  (-0.15 to -0.1) | 0 |
| American Samoa | 2014 | 2019 | 0.52  (0.25 to 0.79) | 0.001 |
| American Samoa | 2019 | 2021 | 5.08  (4.16 to 6.01) | 0 |
| Andorra | 1990 | 2018 | -0.21  (-0.32 to -0.09) | 0.001 |
| Andorra | 2018 | 2021 | 10.87  (6.73 to 15.17) | 0 |
| Angola | 1990 | 2019 | -0.14  (-0.16 to -0.11) | 0 |
| Angola | 2019 | 2021 | 7.99  (5.99 to 10.03) | 0 |
| Antigua and Barbuda | 1990 | 1994 | -1.26  (-1.39 to -1.13) | 0 |
| Antigua and Barbuda | 1994 | 2005 | -0.04  (-0.07 to 0) | 0.036 |
| Antigua and Barbuda | 2005 | 2011 | -0.84  (-0.93 to -0.75) | 0 |
| Antigua and Barbuda | 2011 | 2019 | 1.55  (1.49 to 1.61) | 0 |
| Antigua and Barbuda | 2019 | 2021 | 12.11  (11.58 to 12.64) | 0 |
| Argentina | 1990 | 2006 | 0.08  (0.04 to 0.11) | 0 |
| Argentina | 2006 | 2010 | -2.32  (-2.72 to -1.91) | 0 |
| Argentina | 2010 | 2019 | 0.26  (0.17 to 0.35) | 0 |
| Argentina | 2019 | 2021 | 16.02  (14.96 to 17.08) | 0 |
| Armenia | 1990 | 2011 | -0.13  (-0.18 to -0.08) | 0 |
| Armenia | 2011 | 2019 | 0.91  (0.65 to 1.17) | 0 |
| Armenia | 2019 | 2021 | 16.28  (13.77 to 18.83) | 0 |
| Australia | 1990 | 2000 | 0.75  (0.69 to 0.82) | 0 |
| Australia | 2000 | 2005 | 2.03  (1.77 to 2.29) | 0 |
| Australia | 2005 | 2019 | -0.96  (-1.01 to -0.92) | 0 |
| Australia | 2019 | 2021 | 5.67  (4.59 to 6.76) | 0 |
| Austria | 1990 | 1995 | -0.26  (-0.67 to 0.14) | 0.187 |
| Austria | 1995 | 1999 | -2.47  (-3.32 to -1.6) | 0 |
| Austria | 1999 | 2011 | -1.07  (-1.19 to -0.96) | 0 |
| Austria | 2011 | 2019 | 0.62  (0.38 to 0.86) | 0 |
| Austria | 2019 | 2021 | 12.24  (9.97 to 14.55) | 0 |
| Azerbaijan | 1990 | 1994 | -1.01  (-1.3 to -0.73) | 0 |
| Azerbaijan | 1994 | 2013 | -0.13  (-0.16 to -0.11) | 0 |
| Azerbaijan | 2013 | 2019 | 1.32  (1.12 to 1.52) | 0 |
| Azerbaijan | 2019 | 2021 | 14.09  (12.96 to 15.23) | 0 |
| Bahamas | 1990 | 2012 | -0.26  (-0.32 to -0.2) | 0 |
| Bahamas | 2012 | 2019 | 1.4  (0.98 to 1.83) | 0 |
| Bahamas | 2019 | 2021 | 14.28  (11.16 to 17.48) | 0 |
| Bahrain | 1990 | 2000 | 0.11  (0.05 to 0.18) | 0.001 |
| Bahrain | 2000 | 2014 | -1.07  (-1.11 to -1.03) | 0 |
| Bahrain | 2014 | 2019 | 0.43  (0.16 to 0.7) | 0.003 |
| Bahrain | 2019 | 2021 | 10.61  (9.61 to 11.63) | 0 |
| Bangladesh | 1990 | 2019 | -0.21  (-0.22 to -0.19) | 0 |
| Bangladesh | 2019 | 2021 | 10.37  (9.07 to 11.68) | 0 |
| Barbados | 1990 | 1995 | -0.91  (-1.08 to -0.75) | 0 |
| Barbados | 1995 | 2006 | 0.1  (0.05 to 0.16) | 0.001 |
| Barbados | 2006 | 2010 | -0.57  (-0.93 to -0.22) | 0.003 |
| Barbados | 2010 | 2019 | 0.83  (0.75 to 0.91) | 0 |
| Barbados | 2019 | 2021 | 14.5  (13.58 to 15.44) | 0 |
| Belarus | 1990 | 1995 | 0.72  (0.61 to 0.82) | 0 |
| Belarus | 1995 | 2004 | 0.26  (0.21 to 0.31) | 0 |
| Belarus | 2004 | 2019 | -0.45  (-0.47 to -0.43) | 0 |
| Belarus | 2019 | 2021 | 22.05  (21.41 to 22.68) | 0 |
| Belgium | 1990 | 2001 | 0.13  (-0.37 to 0.64) | 0.6 |
| Belgium | 2001 | 2010 | 1.8  (0.91 to 2.7) | 0 |
| Belgium | 2010 | 2018 | -1.09  (-2.24 to 0.08) | 0.066 |
| Belgium | 2018 | 2021 | 9.2  (4.08 to 14.57) | 0.001 |
| Belize | 1990 | 2018 | 0.1  (0.01 to 0.19) | 0.033 |
| Belize | 2018 | 2021 | 8.35  (5.3 to 11.48) | 0 |
| Benin | 1990 | 2003 | 0.09  (0.06 to 0.11) | 0 |
| Benin | 2003 | 2011 | -0.15  (-0.22 to -0.08) | 0 |
| Benin | 2011 | 2019 | 0.23  (0.16 to 0.3) | 0 |
| Benin | 2019 | 2021 | 4.32  (3.71 to 4.92) | 0 |
| Bermuda | 1990 | 2002 | -0.76  (-0.78 to -0.73) | 0 |
| Bermuda | 2002 | 2010 | -1.28  (-1.34 to -1.22) | 0 |
| Bermuda | 2010 | 2019 | 0.42  (0.37 to 0.47) | 0 |
| Bermuda | 2019 | 2021 | 13.77  (13.17 to 14.38) | 0 |
| Bhutan | 1990 | 2013 | -0.59  (-0.67 to -0.51) | 0 |
| Bhutan | 2013 | 2021 | 1.08  (0.66 to 1.51) | 0 |
| Bolivia (Plurinational State of) | 1990 | 2018 | -0.46  (-0.62 to -0.29) | 0 |
| Bolivia (Plurinational State of) | 2018 | 2021 | 15.26  (9.18 to 21.67) | 0 |
| Bosnia and Herzegovina | 1990 | 2001 | -0.25  (-0.31 to -0.19) | 0 |
| Bosnia and Herzegovina | 2001 | 2013 | -1.85  (-1.91 to -1.79) | 0 |
| Bosnia and Herzegovina | 2013 | 2019 | 0.24  (0.04 to 0.45) | 0.024 |
| Bosnia and Herzegovina | 2019 | 2021 | 16.61  (15.4 to 17.84) | 0 |
| Botswana | 1990 | 2001 | 0.52  (0.43 to 0.61) | 0 |
| Botswana | 2001 | 2019 | -0.31  (-0.35 to -0.26) | 0 |
| Botswana | 2019 | 2021 | 12.27  (10.66 to 13.92) | 0 |
| Brazil | 1990 | 2005 | 1.36  (1.15 to 1.56) | 0 |
| Brazil | 2005 | 2010 | -4.85  (-6.23 to -3.45) | 0 |
| Brazil | 2010 | 2019 | -0.66  (-1.15 to -0.16) | 0.012 |
| Brazil | 2019 | 2021 | 16.59  (11.07 to 22.39) | 0 |
| Brunei Darussalam | 1990 | 1994 | -1.32  (-1.54 to -1.1) | 0 |
| Brunei Darussalam | 1994 | 2005 | -0.06  (-0.11 to 0) | 0.035 |
| Brunei Darussalam | 2005 | 2011 | -0.86  (-1.01 to -0.71) | 0 |
| Brunei Darussalam | 2011 | 2019 | 1.53  (1.44 to 1.63) | 0 |
| Brunei Darussalam | 2019 | 2021 | 8.25  (7.4 to 9.11) | 0 |
| Bulgaria | 1990 | 1997 | -0.16  (-0.24 to -0.08) | 0.001 |
| Bulgaria | 1997 | 2003 | -0.71  (-0.84 to -0.58) | 0 |
| Bulgaria | 2003 | 2010 | -1.55  (-1.65 to -1.45) | 0 |
| Bulgaria | 2010 | 2019 | 0.07  (0 to 0.14) | 0.043 |
| Bulgaria | 2019 | 2021 | 22.47  (21.64 to 23.31) | 0 |
| Burkina Faso | 1990 | 1999 | -0.88  (-1.24 to -0.52) | 0 |
| Burkina Faso | 1999 | 2005 | 1.59  (0.72 to 2.47) | 0.001 |
| Burkina Faso | 2005 | 2011 | -0.26  (-1.12 to 0.6) | 0.529 |
| Burkina Faso | 2011 | 2014 | 2.89  (-1.05 to 6.99) | 0.143 |
| Burkina Faso | 2014 | 2021 | -0.9  (-1.48 to -0.32) | 0.004 |
| Burundi | 1990 | 1996 | -0.18  (-0.28 to -0.09) | 0.001 |
| Burundi | 1996 | 2011 | -1.23  (-1.26 to -1.21) | 0 |
| Burundi | 2011 | 2019 | 0.07  (-0.01 to 0.14) | 0.069 |
| Burundi | 2019 | 2021 | 8.67  (8.01 to 9.34) | 0 |
| Cabo Verde | 1990 | 2019 | 0.2  (0.16 to 0.24) | 0 |
| Cabo Verde | 2019 | 2021 | 12.08  (9.04 to 15.21) | 0 |
| Cambodia | 1990 | 1996 | 0.14  (0 to 0.29) | 0.053 |
| Cambodia | 1996 | 2010 | -1.22  (-1.26 to -1.17) | 0 |
| Cambodia | 2010 | 2019 | -0.31  (-0.4 to -0.22) | 0 |
| Cambodia | 2019 | 2021 | 10.15  (9.17 to 11.14) | 0 |
| Cameroon | 1990 | 2003 | 0.15  (0.09 to 0.22) | 0 |
| Cameroon | 2003 | 2018 | -0.12  (-0.18 to -0.07) | 0 |
| Cameroon | 2018 | 2021 | 2.86  (2.21 to 3.52) | 0 |
| Canada | 1990 | 2000 | 1.21  (1.13 to 1.28) | 0 |
| Canada | 2000 | 2009 | -1.79  (-1.89 to -1.69) | 0 |
| Canada | 2009 | 2019 | 0.89  (0.8 to 0.98) | 0 |
| Canada | 2019 | 2021 | 14.81  (13.45 to 16.18) | 0 |
| Central African Republic | 1990 | 2021 | 0.04  (-0.05 to 0.14) | 0.367 |
| Chad | 1990 | 2004 | 0.3  (0.13 to 0.48) | 0.001 |
| Chad | 2004 | 2017 | -0.26  (-0.47 to -0.05) | 0.018 |
| Chad | 2017 | 2021 | 1.91  (0.62 to 3.22) | 0.005 |
| Chile | 1990 | 2005 | -0.35  (-0.39 to -0.3) | 0 |
| Chile | 2005 | 2010 | -2.26  (-2.64 to -1.88) | 0 |
| Chile | 2010 | 2019 | 0.23  (0.09 to 0.38) | 0.003 |
| Chile | 2019 | 2021 | 14.88  (13.27 to 16.51) | 0 |
| China | 1990 | 1995 | -0.96  (-1.56 to -0.35) | 0.004 |
| China | 1995 | 2000 | -3  (-3.82 to -2.18) | 0 |
| China | 2000 | 2006 | -1.14  (-1.72 to -0.55) | 0.001 |
| China | 2006 | 2009 | -3  (-5.54 to -0.4) | 0.027 |
| China | 2009 | 2021 | 0.44  (0.28 to 0.6) | 0 |
| Colombia | 1990 | 2005 | 1.12  (0.86 to 1.37) | 0 |
| Colombia | 2005 | 2011 | -5.66  (-6.8 to -4.51) | 0 |
| Colombia | 2011 | 2018 | -0.37  (-1.3 to 0.57) | 0.421 |
| Colombia | 2018 | 2021 | 5.9  (2.7 to 9.2) | 0.001 |
| Comoros | 1990 | 2003 | -0.14  (-0.17 to -0.1) | 0 |
| Comoros | 2003 | 2012 | -0.44  (-0.51 to -0.37) | 0 |
| Comoros | 2012 | 2019 | 0.34  (0.23 to 0.45) | 0 |
| Comoros | 2019 | 2021 | 8.52  (7.69 to 9.35) | 0 |
| Congo | 1990 | 1995 | -0.16  (-1.07 to 0.77) | 0.725 |
| Congo | 1995 | 2000 | 1.78  (0.53 to 3.05) | 0.008 |
| Congo | 2000 | 2008 | -0.42  (-0.92 to 0.09) | 0.099 |
| Congo | 2008 | 2018 | -1.25  (-1.61 to -0.9) | 0 |
| Congo | 2018 | 2021 | 5.74  (3.44 to 8.1) | 0 |
| Cook Islands | 1990 | 2019 | 0  (-0.01 to 0.02) | 0.457 |
| Cook Islands | 2019 | 2021 | 5.37  (4.41 to 6.35) | 0 |
| Costa Rica | 1990 | 2019 | 0.23  (0.2 to 0.26) | 0 |
| Costa Rica | 2019 | 2021 | 13.26  (11.19 to 15.37) | 0 |
| Coted'Ivoire | 1990 | 2012 | -0.19  (-0.24 to -0.15) | 0 |
| Coted'Ivoire | 2012 | 2021 | 0.9  (0.7 to 1.1) | 0 |
| Croatia | 1990 | 1996 | -0.46  (-0.54 to -0.37) | 0 |
| Croatia | 1996 | 2009 | -1.56  (-1.59 to -1.53) | 0 |
| Croatia | 2009 | 2019 | 0.01  (-0.04 to 0.05) | 0.729 |
| Croatia | 2019 | 2021 | 13.29  (12.65 to 13.93) | 0 |
| Cuba | 1990 | 1996 | -1.52  (-1.68 to -1.36) | 0 |
| Cuba | 1996 | 2010 | -3.02  (-3.07 to -2.97) | 0 |
| Cuba | 2010 | 2019 | 0.07  (-0.04 to 0.18) | 0.227 |
| Cuba | 2019 | 2021 | 14.17  (12.89 to 15.47) | 0 |
| Cyprus | 1990 | 1994 | -1.13  (-1.4 to -0.85) | 0 |
| Cyprus | 1994 | 2005 | -0.05  (-0.12 to 0.02) | 0.154 |
| Cyprus | 2005 | 2011 | -0.78  (-0.97 to -0.58) | 0 |
| Cyprus | 2011 | 2019 | 1.32  (1.21 to 1.44) | 0 |
| Cyprus | 2019 | 2021 | 9.55  (8.51 to 10.6) | 0 |
| Czechia | 1990 | 1995 | 0  (-0.16 to 0.16) | 0.98 |
| Czechia | 1995 | 2004 | -1.39  (-1.46 to -1.31) | 0 |
| Czechia | 2004 | 2015 | -0.79  (-0.85 to -0.74) | 0 |
| Czechia | 2015 | 2019 | 0.73  (0.37 to 1.1) | 0.001 |
| Czechia | 2019 | 2021 | 13.87  (12.96 to 14.79) | 0 |
| Democratic People's Republic of Korea | 1990 | 1995 | -0.23  (-0.27 to -0.18) | 0 |
| Democratic People's Republic of Korea | 1995 | 2005 | -0.61  (-0.63 to -0.59) | 0 |
| Democratic People's Republic of Korea | 2005 | 2009 | -0.9  (-0.99 to -0.81) | 0 |
| Democratic People's Republic of Korea | 2009 | 2019 | -0.09  (-0.1 to -0.07) | 0 |
| Democratic People's Republic of Korea | 2019 | 2021 | 2.98  (2.78 to 3.19) | 0 |
| Democratic Republic of the Congo | 1990 | 2018 | -0.1  (-0.17 to -0.03) | 0.006 |
| Democratic Republic of the Congo | 2018 | 2021 | 3.66  (1.39 to 5.98) | 0.003 |
| Denmark | 1990 | 1995 | -1.06  (-1.18 to -0.93) | 0 |
| Denmark | 1995 | 2001 | -2.79  (-2.91 to -2.67) | 0 |
| Denmark | 2001 | 2010 | -1.24  (-1.31 to -1.18) | 0 |
| Denmark | 2010 | 2019 | 0.29  (0.23 to 0.36) | 0 |
| Denmark | 2019 | 2021 | 11.5  (10.78 to 12.22) | 0 |
| Djibouti | 1990 | 2018 | -0.04  (-0.08 to 0.01) | 0.083 |
| Djibouti | 2018 | 2021 | 5.05  (3.57 to 6.55) | 0 |
| Dominica | 1990 | 1995 | -0.95  (-1.08 to -0.83) | 0 |
| Dominica | 1995 | 2006 | 0.05  (0 to 0.09) | 0.041 |
| Dominica | 2006 | 2010 | -1.2  (-1.48 to -0.92) | 0 |
| Dominica | 2010 | 2019 | 1.15  (1.09 to 1.21) | 0 |
| Dominica | 2019 | 2021 | 13.24  (12.51 to 13.98) | 0 |
| Dominican Republic | 1990 | 2004 | 0.36  (0.03 to 0.69) | 0.032 |
| Dominican Republic | 2004 | 2018 | -0.5  (-0.88 to -0.13) | 0.01 |
| Dominican Republic | 2018 | 2021 | 8.16  (4.01 to 12.48) | 0 |
| Ecuador | 1990 | 2018 | -0.14  (-0.29 to 0) | 0.045 |
| Ecuador | 2018 | 2021 | 12.15  (7.07 to 17.46) | 0 |
| Egypt | 1990 | 2010 | -0.2  (-0.26 to -0.14) | 0 |
| Egypt | 2010 | 2019 | 0.89  (0.64 to 1.15) | 0 |
| Egypt | 2019 | 2021 | 10.19  (7.14 to 13.33) | 0 |
| El Salvador | 1990 | 2018 | -0.7  (-0.8 to -0.59) | 0 |
| El Salvador | 2018 | 2021 | 9.03  (5.21 to 12.98) | 0 |
| Equatorial Guinea | 1990 | 2018 | -0.08  (-0.15 to -0.02) | 0.018 |
| Equatorial Guinea | 2018 | 2021 | 4.37  (2.12 to 6.68) | 0 |
| Eritrea | 1990 | 1996 | -0.46  (-0.51 to -0.42) | 0 |
| Eritrea | 1996 | 2009 | -0.18  (-0.19 to -0.16) | 0 |
| Eritrea | 2009 | 2015 | -0.08  (-0.14 to -0.02) | 0.014 |
| Eritrea | 2015 | 2019 | 0.42  (0.28 to 0.55) | 0 |
| Eritrea | 2019 | 2021 | 5.22  (4.91 to 5.53) | 0 |
| Estonia | 1990 | 2001 | -0.43  (-0.48 to -0.38) | 0 |
| Estonia | 2001 | 2013 | -2.37  (-2.42 to -2.32) | 0 |
| Estonia | 2013 | 2019 | -0.32  (-0.5 to -0.14) | 0.002 |
| Estonia | 2019 | 2021 | 16.17  (15.14 to 17.21) | 0 |
| Eswatini | 1990 | 2001 | -0.02  (-0.1 to 0.05) | 0.529 |
| Eswatini | 2001 | 2011 | 0.21  (0.11 to 0.31) | 0 |
| Eswatini | 2011 | 2014 | 1.16  (0.06 to 2.26) | 0.04 |
| Eswatini | 2014 | 2019 | -0.07  (-0.43 to 0.3) | 0.698 |
| Eswatini | 2019 | 2021 | 19.2  (17.61 to 20.82) | 0 |
| Ethiopia | 1990 | 2011 | -0.75  (-0.8 to -0.7) | 0 |
| Ethiopia | 2011 | 2019 | 0.5  (0.24 to 0.76) | 0.001 |
| Ethiopia | 2019 | 2021 | 6.78  (4.67 to 8.93) | 0 |
| Fiji | 1990 | 2005 | 0.04  (0.02 to 0.05) | 0 |
| Fiji | 2005 | 2010 | -0.66  (-0.76 to -0.56) | 0 |
| Fiji | 2010 | 2019 | 0.05  (0.01 to 0.08) | 0.013 |
| Fiji | 2019 | 2021 | 8.87  (8.47 to 9.26) | 0 |
| Finland | 1990 | 2001 | 0.46  (0.05 to 0.87) | 0.03 |
| Finland | 2001 | 2018 | -1.33  (-1.55 to -1.11) | 0 |
| Finland | 2018 | 2021 | 9.2  (5.64 to 12.87) | 0 |
| France | 1990 | 2001 | 0.22  (-0.18 to 0.62) | 0.271 |
| France | 2001 | 2018 | -1.03  (-1.29 to -0.78) | 0 |
| France | 2018 | 2021 | 9.05  (4.93 to 13.34) | 0 |
| Gabon | 1990 | 1994 | -0.14  (-0.47 to 0.18) | 0.356 |
| Gabon | 1994 | 2005 | 0.38  (0.3 to 0.45) | 0 |
| Gabon | 2005 | 2010 | -1.48  (-1.78 to -1.17) | 0 |
| Gabon | 2010 | 2019 | -0.04  (-0.15 to 0.07) | 0.462 |
| Gabon | 2019 | 2021 | 8.26  (7.05 to 9.48) | 0 |
| Gambia | 1990 | 1994 | 2.01  (1.87 to 2.15) | 0 |
| Gambia | 1994 | 2005 | -0.36  (-0.4 to -0.33) | 0 |
| Gambia | 2005 | 2010 | -2.45  (-2.58 to -2.31) | 0 |
| Gambia | 2010 | 2019 | 0.18  (0.13 to 0.23) | 0 |
| Gambia | 2019 | 2021 | 6.79  (6.26 to 7.33) | 0 |
| Georgia | 1990 | 1997 | -0.44  (-0.48 to -0.39) | 0 |
| Georgia | 1997 | 2010 | -0.18  (-0.2 to -0.16) | 0 |
| Georgia | 2010 | 2015 | 0.42  (0.31 to 0.52) | 0 |
| Georgia | 2015 | 2019 | 0.68  (0.51 to 0.85) | 0 |
| Georgia | 2019 | 2021 | 14.07  (13.66 to 14.48) | 0 |
| Germany | 1990 | 2000 | 0.03  (-0.15 to 0.21) | 0.761 |
| Germany | 2000 | 2004 | 4.24  (3.05 to 5.45) | 0 |
| Germany | 2004 | 2016 | 0.63  (0.47 to 0.8) | 0 |
| Germany | 2016 | 2019 | -2.76  (-5.33 to -0.12) | 0.042 |
| Germany | 2019 | 2021 | 10.04  (6.81 to 13.36) | 0 |
| Ghana | 1990 | 2003 | 0.21  (0.08 to 0.33) | 0.003 |
| Ghana | 2003 | 2018 | -0.39  (-0.5 to -0.28) | 0 |
| Ghana | 2018 | 2021 | 4.39  (3.01 to 5.78) | 0 |
| Greece | 1990 | 1996 | 0.19  (-0.97 to 1.36) | 0.735 |
| Greece | 1996 | 1999 | 7.53  (0.56 to 14.99) | 0.035 |
| Greece | 1999 | 2011 | -1.07  (-1.48 to -0.65) | 0 |
| Greece | 2011 | 2015 | -5.23  (-8 to -2.39) | 0.001 |
| Greece | 2015 | 2021 | 5.41  (4.21 to 6.63) | 0 |
| Greenland | 1990 | 1998 | 0.29  (0.12 to 0.46) | 0.002 |
| Greenland | 1998 | 2019 | -0.77  (-0.81 to -0.73) | 0 |
| Greenland | 2019 | 2021 | 16.28  (14.22 to 18.37) | 0 |
| Grenada | 1990 | 1995 | -0.56  (-0.67 to -0.44) | 0 |
| Grenada | 1995 | 2005 | 0.21  (0.16 to 0.25) | 0 |
| Grenada | 2005 | 2010 | -0.78  (-0.94 to -0.62) | 0 |
| Grenada | 2010 | 2019 | 0.7  (0.64 to 0.75) | 0 |
| Grenada | 2019 | 2021 | 11.65  (11.01 to 12.29) | 0 |
| Guam | 1990 | 2000 | -0.49  (-0.55 to -0.43) | 0 |
| Guam | 2000 | 2019 | 0.17  (0.14 to 0.19) | 0 |
| Guam | 2019 | 2021 | 10.79  (9.85 to 11.75) | 0 |
| Guatemala | 1990 | 2004 | 0.98  (0.72 to 1.24) | 0 |
| Guatemala | 2004 | 2018 | -1.22  (-1.5 to -0.94) | 0 |
| Guatemala | 2018 | 2021 | 9.28  (5.89 to 12.78) | 0 |
| Guinea | 1990 | 2018 | -0.01  (-0.05 to 0.03) | 0.581 |
| Guinea | 2018 | 2021 | 4.21  (2.98 to 5.44) | 0 |
| Guinea-Bissau | 1990 | 2018 | 0.03  (-0.01 to 0.08) | 0.155 |
| Guinea-Bissau | 2018 | 2021 | 3.74  (2.2 to 5.31) | 0 |
| Guyana | 1990 | 1995 | 0.31  (0.17 to 0.45) | 0 |
| Guyana | 1995 | 2004 | 1.03  (0.97 to 1.1) | 0 |
| Guyana | 2004 | 2014 | 0.17  (0.11 to 0.22) | 0 |
| Guyana | 2014 | 2019 | -0.97  (-1.17 to -0.78) | 0 |
| Guyana | 2019 | 2021 | 17.05  (16.24 to 17.88) | 0 |
| Haiti | 1990 | 2019 | -0.2  (-0.23 to -0.17) | 0 |
| Haiti | 2019 | 2021 | 11.87  (9.78 to 14) | 0 |
| Honduras | 1990 | 2019 | 0.08  (0.06 to 0.1) | 0 |
| Honduras | 2019 | 2021 | 17.53  (15.99 to 19.09) | 0 |
| Hungary | 1990 | 1995 | -0.83  (-0.92 to -0.75) | 0 |
| Hungary | 1995 | 2008 | -1.56  (-1.59 to -1.54) | 0 |
| Hungary | 2008 | 2014 | -1.2  (-1.29 to -1.12) | 0 |
| Hungary | 2014 | 2019 | 0.15  (0.02 to 0.28) | 0.023 |
| Hungary | 2019 | 2021 | 12.47  (11.98 to 12.97) | 0 |
| Iceland | 1990 | 2011 | -0.86  (-0.96 to -0.77) | 0 |
| Iceland | 2011 | 2018 | 0.32  (-0.32 to 0.97) | 0.31 |
| Iceland | 2018 | 2021 | 4.78  (2.67 to 6.94) | 0 |
| India | 1990 | 1994 | 2.87  (2.41 to 3.32) | 0 |
| India | 1994 | 2006 | -0.39  (-0.48 to -0.29) | 0 |
| India | 2006 | 2010 | -6.59  (-7.22 to -5.96) | 0 |
| India | 2010 | 2019 | -0.05  (-0.2 to 0.09) | 0.459 |
| India | 2019 | 2021 | 12.77  (11.22 to 14.33) | 0 |
| Indonesia | 1990 | 1996 | -0.49  (-0.58 to -0.39) | 0 |
| Indonesia | 1996 | 2005 | -0.02  (-0.09 to 0.04) | 0.45 |
| Indonesia | 2005 | 2011 | -0.53  (-0.66 to -0.4) | 0 |
| Indonesia | 2011 | 2019 | 0.92  (0.84 to 1) | 0 |
| Indonesia | 2019 | 2021 | 11.07  (10.42 to 11.73) | 0 |
| Iran (Islamic Republic of) | 1990 | 2001 | -0.89  (-1.15 to -0.63) | 0 |
| Iran (Islamic Republic of) | 2001 | 2019 | 1.07  (0.93 to 1.21) | 0 |
| Iran (Islamic Republic of) | 2019 | 2021 | 5.42  (1.19 to 9.82) | 0.014 |
| Iraq | 1990 | 1996 | -0.18  (-2.08 to 1.75) | 0.844 |
| Iraq | 1996 | 1999 | -6.48  (-15.93 to 4.03) | 0.205 |
| Iraq | 1999 | 2009 | -0.27  (-1.2 to 0.66) | 0.553 |
| Iraq | 2009 | 2021 | 3.06  (2.38 to 3.75) | 0 |
| Ireland | 1990 | 2008 | 0.43  (0.38 to 0.48) | 0 |
| Ireland | 2008 | 2015 | -1.04  (-1.29 to -0.79) | 0 |
| Ireland | 2015 | 2019 | 2.75  (1.84 to 3.66) | 0 |
| Ireland | 2019 | 2021 | 13.99  (11.7 to 16.33) | 0 |
| Israel | 1990 | 1994 | 3.03  (2.75 to 3.31) | 0 |
| Israel | 1994 | 2010 | -0.34  (-0.38 to -0.31) | 0 |
| Israel | 2010 | 2015 | -2.75  (-3.01 to -2.49) | 0 |
| Israel | 2015 | 2019 | 1.14  (0.68 to 1.6) | 0 |
| Israel | 2019 | 2021 | 9.38  (8.26 to 10.52) | 0 |
| Italy | 1990 | 1994 | 2.63  (2.27 to 3) | 0 |
| Italy | 1994 | 2005 | -0.62  (-0.71 to -0.53) | 0 |
| Italy | 2005 | 2010 | -3.12  (-3.46 to -2.77) | 0 |
| Italy | 2010 | 2019 | 1.41  (1.28 to 1.54) | 0 |
| Italy | 2019 | 2021 | 14.9  (13.6 to 16.21) | 0 |
| Jamaica | 1990 | 1994 | -1.39  (-1.56 to -1.22) | 0 |
| Jamaica | 1994 | 2006 | 0  (-0.04 to 0.04) | 0.938 |
| Jamaica | 2006 | 2010 | -1.27  (-1.53 to -1) | 0 |
| Jamaica | 2010 | 2019 | 1.22  (1.16 to 1.28) | 0 |
| Jamaica | 2019 | 2021 | 13.74  (13.05 to 14.43) | 0 |
| Japan | 1990 | 1995 | -0.79  (-1.27 to -0.3) | 0.003 |
| Japan | 1995 | 2000 | 3.88  (3.17 to 4.6) | 0 |
| Japan | 2000 | 2005 | 0.78  (0.09 to 1.48) | 0.028 |
| Japan | 2005 | 2019 | -0.92  (-1.03 to -0.81) | 0 |
| Japan | 2019 | 2021 | 13.39  (10.85 to 15.98) | 0 |
| Jordan | 1990 | 2002 | -0.23  (-0.28 to -0.19) | 0 |
| Jordan | 2002 | 2011 | -1.2  (-1.28 to -1.11) | 0 |
| Jordan | 2011 | 2019 | 0.46  (0.35 to 0.56) | 0 |
| Jordan | 2019 | 2021 | 6.87  (5.93 to 7.81) | 0 |
| Kazakhstan | 1990 | 1994 | -1.73  (-2.68 to -0.76) | 0.001 |
| Kazakhstan | 1994 | 2002 | 0.62  (0.21 to 1.02) | 0.005 |
| Kazakhstan | 2002 | 2009 | -0.24  (-0.74 to 0.26) | 0.318 |
| Kazakhstan | 2009 | 2019 | 0.92  (0.63 to 1.21) | 0 |
| Kazakhstan | 2019 | 2021 | 8.31  (4.63 to 12.12) | 0 |
| Kenya | 1990 | 2002 | 0.16  (-0.1 to 0.43) | 0.217 |
| Kenya | 2002 | 2017 | -0.86  (-1.06 to -0.65) | 0 |
| Kenya | 2017 | 2021 | 3.9  (2.42 to 5.39) | 0 |
| Kiribati | 1990 | 1999 | -0.66  (-0.7 to -0.62) | 0 |
| Kiribati | 1999 | 2012 | -0.38  (-0.41 to -0.35) | 0 |
| Kiribati | 2012 | 2019 | 0.13  (0.05 to 0.21) | 0.003 |
| Kiribati | 2019 | 2021 | 4.97  (4.44 to 5.5) | 0 |
| Kuwait | 1990 | 2001 | 0.12  (-0.22 to 0.46) | 0.472 |
| Kuwait | 2001 | 2016 | -0.52  (-0.75 to -0.29) | 0 |
| Kuwait | 2016 | 2021 | 1.76  (0.55 to 2.98) | 0.006 |
| Kyrgyzstan | 1990 | 2019 | -0.19  (-0.21 to -0.16) | 0 |
| Kyrgyzstan | 2019 | 2021 | 14.79  (12.81 to 16.8) | 0 |
| Lao People's Democratic Republic | 1990 | 1996 | -0.41  (-1.12 to 0.32) | 0.254 |
| Lao People's Democratic Republic | 1996 | 1999 | -4.22  (-8.15 to -0.12) | 0.044 |
| Lao People's Democratic Republic | 1999 | 2010 | -0.49  (-0.81 to -0.17) | 0.005 |
| Lao People's Democratic Republic | 2010 | 2019 | 0.77  (0.31 to 1.24) | 0.003 |
| Lao People's Democratic Republic | 2019 | 2021 | 5.36  (0.56 to 10.39) | 0.03 |
| Latvia | 1990 | 2014 | -0.79  (-0.85 to -0.73) | 0 |
| Latvia | 2014 | 2019 | 0.41  (-0.48 to 1.3) | 0.349 |
| Latvia | 2019 | 2021 | 14.94  (11.22 to 18.78) | 0 |
| Lebanon | 1990 | 1994 | 2.76  (2.44 to 3.08) | 0 |
| Lebanon | 1994 | 2000 | 1.66  (1.44 to 1.87) | 0 |
| Lebanon | 2000 | 2015 | -0.17  (-0.21 to -0.13) | 0 |
| Lebanon | 2015 | 2019 | -2.19  (-2.67 to -1.72) | 0 |
| Lebanon | 2019 | 2021 | 18.89  (17.55 to 20.25) | 0 |
| Lesotho | 1990 | 1994 | -3.23  (-3.57 to -2.89) | 0 |
| Lesotho | 1994 | 2019 | 0.26  (0.24 to 0.29) | 0 |
| Lesotho | 2019 | 2021 | 15.55  (14.25 to 16.86) | 0 |
| Liberia | 1990 | 2001 | -0.05  (-0.06 to -0.03) | 0 |
| Liberia | 2001 | 2004 | 5.68  (5.46 to 5.89) | 0 |
| Liberia | 2004 | 2015 | 0.11  (0.1 to 0.13) | 0 |
| Liberia | 2015 | 2019 | -4.28  (-4.38 to -4.18) | 0 |
| Liberia | 2019 | 2021 | 3.61  (3.34 to 3.88) | 0 |
| Libya | 1990 | 2018 | -0.09  (-0.16 to -0.01) | 0.026 |
| Libya | 2018 | 2021 | 5.7  (3.17 to 8.29) | 0 |
| Lithuania | 1990 | 2000 | 0.29  (0.25 to 0.33) | 0 |
| Lithuania | 2000 | 2008 | -1.04  (-1.1 to -0.98) | 0 |
| Lithuania | 2008 | 2015 | -0.75  (-0.83 to -0.67) | 0 |
| Lithuania | 2015 | 2019 | -0.1  (-0.33 to 0.13) | 0.375 |
| Lithuania | 2019 | 2021 | 20.89  (20.27 to 21.51) | 0 |
| Luxembourg | 1990 | 2013 | -1.26  (-1.32 to -1.2) | 0 |
| Luxembourg | 2013 | 2019 | 0.6  (0.01 to 1.19) | 0.047 |
| Luxembourg | 2019 | 2021 | 11.4  (8.2 to 14.68) | 0 |
| Madagascar | 1990 | 2014 | -0.23  (-0.25 to -0.21) | 0 |
| Madagascar | 2014 | 2019 | 0.49  (0.17 to 0.81) | 0.004 |
| Madagascar | 2019 | 2021 | 7.84  (6.61 to 9.09) | 0 |
| Malawi | 1990 | 1995 | 0  (-0.07 to 0.08) | 0.925 |
| Malawi | 1995 | 2005 | -0.44  (-0.47 to -0.41) | 0 |
| Malawi | 2005 | 2010 | -0.94  (-1.04 to -0.84) | 0 |
| Malawi | 2010 | 2019 | 0.2  (0.16 to 0.24) | 0 |
| Malawi | 2019 | 2021 | 8.19  (7.78 to 8.6) | 0 |
| Malaysia | 1990 | 2001 | -1.26  (-1.69 to -0.83) | 0 |
| Malaysia | 2001 | 2004 | 7.64  (1.41 to 14.26) | 0.018 |
| Malaysia | 2004 | 2010 | -0.1  (-1.4 to 1.22) | 0.876 |
| Malaysia | 2010 | 2016 | 1.91  (0.56 to 3.28) | 0.008 |
| Malaysia | 2016 | 2021 | -1.51  (-2.97 to -0.02) | 0.047 |
| Maldives | 1990 | 1995 | -0.47  (-0.62 to -0.31) | 0 |
| Maldives | 1995 | 2010 | -1.71  (-1.74 to -1.68) | 0 |
| Maldives | 2010 | 2019 | -0.53  (-0.6 to -0.45) | 0 |
| Maldives | 2019 | 2021 | 9.66  (8.82 to 10.5) | 0 |
| Mali | 1990 | 2010 | -0.47  (-0.49 to -0.45) | 0 |
| Mali | 2010 | 2019 | 0.65  (0.56 to 0.73) | 0 |
| Mali | 2019 | 2021 | 3.98  (3.09 to 4.88) | 0 |
| Malta | 1990 | 2012 | -0.35  (-0.42 to -0.28) | 0 |
| Malta | 2012 | 2019 | 1.63  (1.13 to 2.13) | 0 |
| Malta | 2019 | 2021 | 8.93  (5.55 to 12.41) | 0 |
| Marshall Islands | 1990 | 2005 | 0.02  (-0.01 to 0.04) | 0.209 |
| Marshall Islands | 2005 | 2012 | -0.66  (-0.76 to -0.56) | 0 |
| Marshall Islands | 2012 | 2019 | 0.05  (-0.05 to 0.15) | 0.326 |
| Marshall Islands | 2019 | 2021 | 4.95  (4.3 to 5.6) | 0 |
| Mauritania | 1990 | 2011 | -0.42  (-0.44 to -0.39) | 0 |
| Mauritania | 2011 | 2019 | 0.65  (0.52 to 0.78) | 0 |
| Mauritania | 2019 | 2021 | 5.1  (3.94 to 6.27) | 0 |
| Mauritius | 1990 | 2001 | 0.42  (0.29 to 0.55) | 0 |
| Mauritius | 2001 | 2010 | -1.1  (-1.3 to -0.89) | 0 |
| Mauritius | 2010 | 2015 | -2  (-2.6 to -1.39) | 0 |
| Mauritius | 2015 | 2019 | 0.28  (-0.73 to 1.31) | 0.564 |
| Mauritius | 2019 | 2021 | 12.13  (9.59 to 14.73) | 0 |
| Mexico | 1990 | 2005 | 0.3  (0.23 to 0.37) | 0 |
| Mexico | 2005 | 2010 | 4.67  (4.15 to 5.2) | 0 |
| Mexico | 2010 | 2019 | 0.86  (0.69 to 1.04) | 0 |
| Mexico | 2019 | 2021 | 15.66  (13.81 to 17.54) | 0 |
| Micronesia (Federated States of) | 1990 | 1997 | -0.46  (-0.52 to -0.39) | 0 |
| Micronesia (Federated States of) | 1997 | 2005 | -0.13  (-0.2 to -0.07) | 0 |
| Micronesia (Federated States of) | 2005 | 2012 | -0.54  (-0.63 to -0.46) | 0 |
| Micronesia (Federated States of) | 2012 | 2019 | 0.18  (0.1 to 0.27) | 0 |
| Micronesia (Federated States of) | 2019 | 2021 | 4.7  (4.15 to 5.26) | 0 |
| Monaco | 1990 | 2019 | 0.02  (0.01 to 0.02) | 0.001 |
| Monaco | 2019 | 2021 | 13.46  (12.84 to 14.08) | 0 |
| Mongolia | 1990 | 1995 | 0.27  (-0.02 to 0.57) | 0.069 |
| Mongolia | 1995 | 2019 | -0.35  (-0.37 to -0.32) | 0 |
| Mongolia | 2019 | 2021 | 2.73  (1.26 to 4.23) | 0.001 |
| Montenegro | 1990 | 2000 | 0.06  (-0.05 to 0.18) | 0.258 |
| Montenegro | 2000 | 2019 | -0.28  (-0.33 to -0.24) | 0 |
| Montenegro | 2019 | 2021 | 18.53  (16.63 to 20.46) | 0 |
| Morocco | 1990 | 2018 | -0.27  (-0.38 to -0.16) | 0 |
| Morocco | 2018 | 2021 | 8.49  (4.74 to 12.37) | 0 |
| Mozambique | 1990 | 1997 | -0.12  (-0.15 to -0.08) | 0 |
| Mozambique | 1997 | 2007 | 0.11  (0.09 to 0.13) | 0 |
| Mozambique | 2007 | 2015 | -0.14  (-0.18 to -0.11) | 0 |
| Mozambique | 2015 | 2019 | 0.17  (0.04 to 0.3) | 0.015 |
| Mozambique | 2019 | 2021 | 8.56  (8.24 to 8.88) | 0 |
| Myanmar | 1990 | 1995 | -0.35  (-0.85 to 0.15) | 0.161 |
| Myanmar | 1995 | 1999 | -2.02  (-3.09 to -0.94) | 0.001 |
| Myanmar | 1999 | 2010 | -0.16  (-0.33 to 0.01) | 0.059 |
| Myanmar | 2010 | 2019 | 1.47  (1.22 to 1.71) | 0 |
| Myanmar | 2019 | 2021 | 11.11  (8.55 to 13.72) | 0 |
| Namibia | 1990 | 1995 | 0.62  (-0.05 to 1.3) | 0.069 |
| Namibia | 1995 | 1999 | -2.22  (-3.64 to -0.79) | 0.005 |
| Namibia | 1999 | 2010 | -0.57  (-0.79 to -0.36) | 0 |
| Namibia | 2010 | 2019 | 1.13  (0.81 to 1.46) | 0 |
| Namibia | 2019 | 2021 | 14.67  (10.8 to 18.67) | 0 |
| Nauru | 1990 | 2019 | 0.02  (0.01 to 0.04) | 0.005 |
| Nauru | 2019 | 2021 | 5.26  (4.17 to 6.36) | 0 |
| Nepal | 1990 | 1995 | -0.27  (-1.59 to 1.07) | 0.673 |
| Nepal | 1995 | 2000 | 2.65  (0.83 to 4.51) | 0.006 |
| Nepal | 2000 | 2009 | 0.63  (0.03 to 1.23) | 0.039 |
| Nepal | 2009 | 2018 | -1.32  (-1.93 to -0.72) | 0 |
| Nepal | 2018 | 2021 | 10.44  (7.03 to 13.96) | 0 |
| Netherlands | 1990 | 2010 | -0.11  (-0.16 to -0.07) | 0 |
| Netherlands | 2010 | 2015 | -2.49  (-3.01 to -1.97) | 0 |
| Netherlands | 2015 | 2019 | 2.86  (1.93 to 3.81) | 0 |
| Netherlands | 2019 | 2021 | 13.9  (11.42 to 16.43) | 0 |
| New Zealand | 1990 | 1993 | 1.31  (0.16 to 2.48) | 0.027 |
| New Zealand | 1993 | 2012 | 0.01  (-0.05 to 0.08) | 0.676 |
| New Zealand | 2012 | 2021 | 0.57  (0.36 to 0.77) | 0 |
| Nicaragua | 1990 | 2001 | 0.59  (0.16 to 1.01) | 0.009 |
| Nicaragua | 2001 | 2018 | -0.53  (-0.77 to -0.29) | 0 |
| Nicaragua | 2018 | 2021 | 8.67  (5.13 to 12.34) | 0 |
| Niger | 1990 | 2021 | -0.03  (-0.08 to 0.01) | 0.158 |
| Nigeria | 1990 | 2004 | 0.24  (0.01 to 0.47) | 0.043 |
| Nigeria | 2004 | 2014 | -2.02  (-2.45 to -1.58) | 0 |
| Nigeria | 2014 | 2021 | 0.82  (0.18 to 1.47) | 0.014 |
| Niue | 1990 | 2019 | 0  (-0.02 to 0.02) | 0.874 |
| Niue | 2019 | 2021 | 5.31  (4.18 to 6.45) | 0 |
| North Macedonia | 1990 | 2012 | -0.56  (-0.6 to -0.53) | 0 |
| North Macedonia | 2012 | 2019 | 0.6  (0.35 to 0.85) | 0 |
| North Macedonia | 2019 | 2021 | 21.07  (19.11 to 23.07) | 0 |
| Northern Mariana Islands | 1990 | 1995 | -0.69  (-0.87 to -0.5) | 0 |
| Northern Mariana Islands | 1995 | 2005 | 0.34  (0.27 to 0.42) | 0 |
| Northern Mariana Islands | 2005 | 2011 | -0.56  (-0.74 to -0.38) | 0 |
| Northern Mariana Islands | 2011 | 2019 | 0.59  (0.48 to 0.7) | 0 |
| Northern Mariana Islands | 2019 | 2021 | 10.68  (9.7 to 11.67) | 0 |
| Norway | 1990 | 1995 | 0.82  (0 to 1.64) | 0.051 |
| Norway | 1995 | 2000 | -1.31  (-2.43 to -0.17) | 0.027 |
| Norway | 2000 | 2004 | 1.4  (-0.41 to 3.25) | 0.123 |
| Norway | 2004 | 2016 | -0.25  (-0.5 to 0) | 0.053 |
| Norway | 2016 | 2021 | 4.13  (3.25 to 5.01) | 0 |
| Oman | 1990 | 2012 | -0.2  (-0.22 to -0.18) | 0 |
| Oman | 2012 | 2019 | 0.95  (0.82 to 1.08) | 0 |
| Oman | 2019 | 2021 | 9.99  (9.04 to 10.94) | 0 |
| Pakistan | 1990 | 2006 | 0.43  (0.18 to 0.67) | 0.002 |
| Pakistan | 2006 | 2016 | -1.27  (-1.85 to -0.68) | 0 |
| Pakistan | 2016 | 2021 | 3.96  (2.44 to 5.5) | 0 |
| Palau | 1990 | 2019 | 0  (-0.01 to 0.02) | 0.522 |
| Palau | 2019 | 2021 | 5.45  (4.34 to 6.57) | 0 |
| Palestine | 1990 | 1995 | -0.12  (-0.42 to 0.19) | 0.43 |
| Palestine | 1995 | 2000 | 1.91  (1.51 to 2.32) | 0 |
| Palestine | 2000 | 2008 | -0.22  (-0.38 to -0.06) | 0.011 |
| Palestine | 2008 | 2019 | -1.06  (-1.16 to -0.96) | 0 |
| Palestine | 2019 | 2021 | 11.14  (9.51 to 12.8) | 0 |
| Panama | 1990 | 2018 | -0.06  (-0.19 to 0.07) | 0.374 |
| Panama | 2018 | 2021 | 10.44  (5.87 to 15.21) | 0 |
| Papua New Guinea | 1990 | 2006 | -0.09  (-0.11 to -0.06) | 0 |
| Papua New Guinea | 2006 | 2010 | -0.65  (-0.97 to -0.34) | 0 |
| Papua New Guinea | 2010 | 2019 | -0.13  (-0.2 to -0.06) | 0.001 |
| Papua New Guinea | 2019 | 2021 | 2.1  (1.4 to 2.8) | 0 |
| Paraguay | 1990 | 2019 | 0.21  (0.19 to 0.22) | 0 |
| Paraguay | 2019 | 2021 | 15.11  (13.67 to 16.58) | 0 |
| Peru | 1990 | 2018 | -0.7  (-0.87 to -0.52) | 0 |
| Peru | 2018 | 2021 | 16.54  (9.9 to 23.58) | 0 |
| Philippines | 1990 | 2005 | -0.8  (-0.85 to -0.75) | 0 |
| Philippines | 2005 | 2019 | 0.16  (0.1 to 0.22) | 0 |
| Philippines | 2019 | 2021 | 13.1  (11.75 to 14.47) | 0 |
| Poland | 1990 | 2000 | 0.09  (0.04 to 0.14) | 0.003 |
| Poland | 2000 | 2006 | -2.23  (-2.38 to -2.07) | 0 |
| Poland | 2006 | 2015 | -0.22  (-0.29 to -0.14) | 0 |
| Poland | 2015 | 2019 | 2.8  (2.44 to 3.16) | 0 |
| Poland | 2019 | 2021 | 12.27  (11.48 to 13.07) | 0 |
| Portugal | 1990 | 1995 | -0.79  (-2.72 to 1.18) | 0.412 |
| Portugal | 1995 | 1999 | 3.6  (-0.82 to 8.22) | 0.106 |
| Portugal | 1999 | 2018 | -1.67  (-1.92 to -1.42) | 0 |
| Portugal | 2018 | 2021 | 12.68  (7.38 to 18.25) | 0 |
| Puerto Rico | 1990 | 2013 | -0.34  (-0.42 to -0.26) | 0 |
| Puerto Rico | 2013 | 2019 | 1.66  (0.84 to 2.48) | 0 |
| Puerto Rico | 2019 | 2021 | 9.77  (5.57 to 14.14) | 0 |
| Qatar | 1990 | 2001 | -0.17  (-0.39 to 0.06) | 0.146 |
| Qatar | 2001 | 2010 | -1.01  (-1.38 to -0.65) | 0 |
| Qatar | 2010 | 2018 | 0.09  (-0.37 to 0.55) | 0.695 |
| Qatar | 2018 | 2021 | 4.5  (2.59 to 6.44) | 0 |
| Republic of Korea | 1990 | 2004 | -0.15  (-0.23 to -0.07) | 0.001 |
| Republic of Korea | 2004 | 2019 | 0.55  (0.47 to 0.63) | 0 |
| Republic of Korea | 2019 | 2021 | 7.41  (5.24 to 9.63) | 0 |
| Republic of Moldova | 1990 | 1995 | -0.39  (-0.66 to -0.13) | 0.006 |
| Republic of Moldova | 1995 | 2008 | -1.28  (-1.35 to -1.21) | 0 |
| Republic of Moldova | 2008 | 2019 | 0  (-0.09 to 0.09) | 0.992 |
| Republic of Moldova | 2019 | 2021 | 15.6  (14.01 to 17.21) | 0 |
| Romania | 1990 | 2012 | -0.37  (-0.4 to -0.35) | 0 |
| Romania | 2012 | 2019 | 0.48  (0.29 to 0.66) | 0 |
| Romania | 2019 | 2021 | 17.34  (15.92 to 18.78) | 0 |
| Russian Federation | 1990 | 1993 | 0.56  (-1.31 to 2.46) | 0.542 |
| Russian Federation | 1993 | 2010 | -0.9  (-1.04 to -0.76) | 0 |
| Russian Federation | 2010 | 2014 | 3.06  (1.15 to 5.01) | 0.003 |
| Russian Federation | 2014 | 2019 | -0.07  (-1.26 to 1.13) | 0.9 |
| Russian Federation | 2019 | 2021 | 15.11  (10.79 to 19.6) | 0 |
| Rwanda | 1990 | 1994 | 0.99  (0.66 to 1.32) | 0 |
| Rwanda | 1994 | 2003 | -0.8  (-0.9 to -0.69) | 0 |
| Rwanda | 2003 | 2010 | -2.18  (-2.35 to -2.01) | 0 |
| Rwanda | 2010 | 2019 | 0.21  (0.09 to 0.32) | 0.001 |
| Rwanda | 2019 | 2021 | 8.17  (6.9 to 9.46) | 0 |
| Saint Kitts and Nevis | 1990 | 2019 | 0.06  (0.04 to 0.08) | 0 |
| Saint Kitts and Nevis | 2019 | 2021 | 11.22  (10.01 to 12.45) | 0 |
| Saint Lucia | 1990 | 1996 | -0.76  (-0.86 to -0.65) | 0 |
| Saint Lucia | 1996 | 2006 | 0.05  (-0.01 to 0.1) | 0.098 |
| Saint Lucia | 2006 | 2010 | -0.58  (-0.88 to -0.27) | 0.001 |
| Saint Lucia | 2010 | 2019 | 0.83  (0.76 to 0.9) | 0 |
| Saint Lucia | 2019 | 2021 | 15.97  (15.16 to 16.79) | 0 |
| Saint Vincent and the Grenadines | 1990 | 1994 | -0.98  (-1.1 to -0.86) | 0 |
| Saint Vincent and the Grenadines | 1994 | 2006 | 0.04  (0.01 to 0.07) | 0.004 |
| Saint Vincent and the Grenadines | 2006 | 2010 | -0.69  (-0.88 to -0.49) | 0 |
| Saint Vincent and the Grenadines | 2010 | 2019 | 0.88  (0.83 to 0.92) | 0 |
| Saint Vincent and the Grenadines | 2019 | 2021 | 12.97  (12.49 to 13.46) | 0 |
| Samoa | 1990 | 2000 | -0.42  (-0.46 to -0.38) | 0 |
| Samoa | 2000 | 2006 | -0.14  (-0.26 to -0.02) | 0.029 |
| Samoa | 2006 | 2010 | -0.89  (-1.16 to -0.63) | 0 |
| Samoa | 2010 | 2019 | 0.14  (0.08 to 0.2) | 0 |
| Samoa | 2019 | 2021 | 5.4  (4.76 to 6.04) | 0 |
| San Marino | 1990 | 2019 | 0.05  (0.04 to 0.06) | 0 |
| San Marino | 2019 | 2021 | 16.29  (15.36 to 17.22) | 0 |
| Sao Tome and Principe | 1990 | 2016 | -0.08  (-0.14 to -0.02) | 0.009 |
| Sao Tome and Principe | 2016 | 2021 | 2.35  (1.62 to 3.1) | 0 |
| Saudi Arabia | 1990 | 2018 | 0.13  (0.02 to 0.25) | 0.024 |
| Saudi Arabia | 2018 | 2021 | 5.77  (2.08 to 9.6) | 0.003 |
| Senegal | 1990 | 2010 | -0.26  (-0.31 to -0.22) | 0 |
| Senegal | 2010 | 2019 | 0.84  (0.65 to 1.03) | 0 |
| Senegal | 2019 | 2021 | 8.39  (6.25 to 10.56) | 0 |
| Serbia | 1990 | 1997 | -0.37  (-0.47 to -0.26) | 0 |
| Serbia | 1997 | 2011 | -0.99  (-1.03 to -0.95) | 0 |
| Serbia | 2011 | 2019 | 0.16  (0.05 to 0.27) | 0.005 |
| Serbia | 2019 | 2021 | 13.44  (12.41 to 14.47) | 0 |
| Seychelles | 1990 | 1998 | -0.42  (-0.47 to -0.37) | 0 |
| Seychelles | 1998 | 2004 | -0.2  (-0.3 to -0.1) | 0.001 |
| Seychelles | 2004 | 2011 | -0.5  (-0.57 to -0.42) | 0 |
| Seychelles | 2011 | 2019 | 0.74  (0.68 to 0.8) | 0 |
| Seychelles | 2019 | 2021 | 11.98  (11.42 to 12.55) | 0 |
| Sierra Leone | 1990 | 2021 | 0.34  (0.28 to 0.4) | 0 |
| Singapore | 1990 | 2000 | -0.75  (-0.94 to -0.55) | 0 |
| Singapore | 2000 | 2005 | -6.9  (-7.7 to -6.1) | 0 |
| Singapore | 2005 | 2009 | -1.69  (-3.07 to -0.3) | 0.02 |
| Singapore | 2009 | 2018 | -0.35  (-0.65 to -0.05) | 0.026 |
| Singapore | 2018 | 2021 | 4.44  (2.89 to 6.02) | 0 |
| Slovakia | 1990 | 1996 | -0.38  (-0.71 to -0.05) | 0.027 |
| Slovakia | 1996 | 1999 | -2.58  (-4.38 to -0.75) | 0.009 |
| Slovakia | 1999 | 2010 | -0.65  (-0.8 to -0.51) | 0 |
| Slovakia | 2010 | 2019 | 0.97  (0.76 to 1.18) | 0 |
| Slovakia | 2019 | 2021 | 15.69  (13.12 to 18.32) | 0 |
| Slovenia | 1990 | 2001 | -0.61  (-0.71 to -0.51) | 0 |
| Slovenia | 2001 | 2012 | -1.79  (-1.91 to -1.68) | 0 |
| Slovenia | 2012 | 2019 | 0.06  (-0.19 to 0.31) | 0.63 |
| Slovenia | 2019 | 2021 | 13.24  (11.35 to 15.16) | 0 |
| Solomon Islands | 1990 | 2006 | -0.21  (-0.24 to -0.18) | 0 |
| Solomon Islands | 2006 | 2010 | -0.67  (-1.04 to -0.3) | 0.001 |
| Solomon Islands | 2010 | 2019 | -0.11  (-0.19 to -0.02) | 0.013 |
| Solomon Islands | 2019 | 2021 | 5.42  (4.58 to 6.27) | 0 |
| Somalia | 1990 | 2004 | 0.07  (0.04 to 0.1) | 0 |
| Somalia | 2004 | 2010 | -0.38  (-0.51 to -0.25) | 0 |
| Somalia | 2010 | 2015 | 0.17  (-0.02 to 0.35) | 0.073 |
| Somalia | 2015 | 2019 | -0.13  (-0.42 to 0.16) | 0.344 |
| Somalia | 2019 | 2021 | 11.28  (10.55 to 12.01) | 0 |
| South Africa | 1990 | 2005 | -0.75  (-0.92 to -0.57) | 0 |
| South Africa | 2005 | 2019 | 0.94  (0.72 to 1.16) | 0 |
| South Africa | 2019 | 2021 | 13.41  (8.51 to 18.53) | 0 |
| South Sudan | 1990 | 2018 | -0.03  (-0.08 to 0.01) | 0.173 |
| South Sudan | 2018 | 2021 | 3.74  (2.14 to 5.37) | 0 |
| Spain | 1990 | 2005 | -1  (-1.13 to -0.88) | 0 |
| Spain | 2005 | 2011 | 4.82  (4.1 to 5.54) | 0 |
| Spain | 2011 | 2019 | 1.47  (1.07 to 1.87) | 0 |
| Spain | 2019 | 2021 | 11.68  (7.61 to 15.9) | 0 |
| Sri Lanka | 1990 | 1996 | -0.46  (-0.75 to -0.18) | 0.003 |
| Sri Lanka | 1996 | 2000 | -3.5  (-4.28 to -2.72) | 0 |
| Sri Lanka | 2000 | 2009 | -1.75  (-1.92 to -1.57) | 0 |
| Sri Lanka | 2009 | 2019 | 0.54  (0.39 to 0.69) | 0 |
| Sri Lanka | 2019 | 2021 | 11.33  (9.29 to 13.41) | 0 |
| Sudan | 1990 | 2018 | -0.38  (-0.48 to -0.29) | 0 |
| Sudan | 2018 | 2021 | 6.5  (3.43 to 9.66) | 0 |
| Suriname | 1990 | 2001 | 0.24  (0.17 to 0.3) | 0 |
| Suriname | 2001 | 2005 | 0.7  (0.23 to 1.18) | 0.006 |
| Suriname | 2005 | 2009 | -0.48  (-0.94 to -0.01) | 0.046 |
| Suriname | 2009 | 2019 | -0.04  (-0.13 to 0.04) | 0.332 |
| Suriname | 2019 | 2021 | 18.31  (17.05 to 19.58) | 0 |
| Sweden | 1990 | 2009 | 0.24  (0.06 to 0.42) | 0.013 |
| Sweden | 2009 | 2018 | -1.02  (-1.69 to -0.34) | 0.005 |
| Sweden | 2018 | 2021 | 8.01  (4.53 to 11.61) | 0 |
| Switzerland | 1990 | 2000 | -0.62  (-0.7 to -0.54) | 0 |
| Switzerland | 2000 | 2005 | -7.79  (-8.08 to -7.49) | 0 |
| Switzerland | 2005 | 2009 | 5.63  (5.11 to 6.15) | 0 |
| Switzerland | 2009 | 2019 | -0.13  (-0.22 to -0.03) | 0.011 |
| Switzerland | 2019 | 2021 | 13.49  (12.18 to 14.82) | 0 |
| Syrian Arab Republic | 1990 | 2012 | -0.26  (-0.29 to -0.23) | 0 |
| Syrian Arab Republic | 2012 | 2019 | 0.97  (0.74 to 1.2) | 0 |
| Syrian Arab Republic | 2019 | 2021 | 9.24  (7.67 to 10.85) | 0 |
| Taiwan (Province of China) | 1990 | 1998 | 0.31  (0.26 to 0.36) | 0 |
| Taiwan (Province of China) | 1998 | 2005 | 0.85  (0.77 to 0.93) | 0 |
| Taiwan (Province of China) | 2005 | 2010 | 0  (-0.15 to 0.14) | 0.967 |
| Taiwan (Province of China) | 2010 | 2019 | 0.19  (0.14 to 0.24) | 0 |
| Taiwan (Province of China) | 2019 | 2021 | 2.47  (1.95 to 2.98) | 0 |
| Tajikistan | 1990 | 2002 | -0.63  (-0.68 to -0.58) | 0 |
| Tajikistan | 2002 | 2013 | -0.34  (-0.41 to -0.28) | 0 |
| Tajikistan | 2013 | 2019 | 0.61  (0.41 to 0.8) | 0 |
| Tajikistan | 2019 | 2021 | 12.42  (11.31 to 13.55) | 0 |
| Thailand | 1990 | 1996 | 0.13  (0.08 to 0.17) | 0 |
| Thailand | 1996 | 2005 | -0.11  (-0.14 to -0.08) | 0 |
| Thailand | 2005 | 2010 | -0.97  (-1.06 to -0.89) | 0 |
| Thailand | 2010 | 2019 | -0.28  (-0.31 to -0.25) | 0 |
| Thailand | 2019 | 2021 | 6.93  (6.6 to 7.25) | 0 |
| Timor-Leste | 1990 | 1995 | -0.38  (-0.51 to -0.24) | 0 |
| Timor-Leste | 1995 | 2000 | -1.53  (-1.71 to -1.34) | 0 |
| Timor-Leste | 2000 | 2009 | -1  (-1.06 to -0.94) | 0 |
| Timor-Leste | 2009 | 2019 | 0.09  (0.04 to 0.15) | 0.002 |
| Timor-Leste | 2019 | 2021 | 8.72  (7.99 to 9.45) | 0 |
| Togo | 1990 | 2018 | -0.06  (-0.11 to -0.02) | 0.007 |
| Togo | 2018 | 2021 | 2.82  (1.39 to 4.26) | 0 |
| Tokelau | 1990 | 2019 | 0.01  (0 to 0.03) | 0.054 |
| Tokelau | 2019 | 2021 | 5.35  (4.36 to 6.34) | 0 |
| Tonga | 1990 | 1995 | -0.45  (-0.56 to -0.33) | 0 |
| Tonga | 1995 | 2005 | -0.12  (-0.17 to -0.07) | 0 |
| Tonga | 2005 | 2011 | -0.53  (-0.65 to -0.42) | 0 |
| Tonga | 2011 | 2019 | 0.21  (0.15 to 0.28) | 0 |
| Tonga | 2019 | 2021 | 5.09  (4.51 to 5.67) | 0 |
| Trinidad and Tobago | 1990 | 2008 | -0.73  (-0.77 to -0.69) | 0 |
| Trinidad and Tobago | 2008 | 2019 | 0.14  (0.04 to 0.23) | 0.01 |
| Trinidad and Tobago | 2019 | 2021 | 17.93  (16.18 to 19.71) | 0 |
| Tunisia | 1990 | 2003 | -0.59  (-0.63 to -0.55) | 0 |
| Tunisia | 2003 | 2011 | -0.12  (-0.22 to -0.02) | 0.02 |
| Tunisia | 2011 | 2019 | 0.66  (0.56 to 0.77) | 0 |
| Tunisia | 2019 | 2021 | 16.16  (15.14 to 17.2) | 0 |
| Turkey | 1990 | 1999 | 0.23  (-0.01 to 0.46) | 0.058 |
| Turkey | 1999 | 2004 | 1.82  (0.98 to 2.67) | 0 |
| Turkey | 2004 | 2019 | -0.75  (-0.89 to -0.62) | 0 |
| Turkey | 2019 | 2021 | 10.76  (6.87 to 14.79) | 0 |
| Turkmenistan | 1990 | 2013 | -0.36  (-0.37 to -0.35) | 0 |
| Turkmenistan | 2013 | 2019 | 0.28  (0.15 to 0.4) | 0 |
| Turkmenistan | 2019 | 2021 | 11.02  (10.33 to 11.7) | 0 |
| Tuvalu | 1990 | 1994 | -0.35  (-0.58 to -0.12) | 0.004 |
| Tuvalu | 1994 | 2019 | 0.08  (0.06 to 0.09) | 0 |
| Tuvalu | 2019 | 2021 | 4.92  (4.12 to 5.73) | 0 |
| Uganda | 1990 | 1999 | 2  (1.87 to 2.13) | 0 |
| Uganda | 1999 | 2007 | -0.34  (-0.52 to -0.16) | 0.001 |
| Uganda | 2007 | 2015 | -2.76  (-2.94 to -2.57) | 0 |
| Uganda | 2015 | 2019 | 0.17  (-0.58 to 0.93) | 0.633 |
| Uganda | 2019 | 2021 | 8.52  (6.71 to 10.37) | 0 |
| Ukraine | 1990 | 1996 | 1.51  (1.19 to 1.84) | 0 |
| Ukraine | 1996 | 2008 | -0.57  (-0.69 to -0.44) | 0 |
| Ukraine | 2008 | 2015 | -1.9  (-2.2 to -1.59) | 0 |
| Ukraine | 2015 | 2019 | 0.05  (-0.88 to 0.99) | 0.903 |
| Ukraine | 2019 | 2021 | 15.29  (12.98 to 17.64) | 0 |
| United Arab Emirates | 1990 | 2001 | -0.17  (-0.21 to -0.14) | 0 |
| United Arab Emirates | 2001 | 2010 | -1.31  (-1.36 to -1.25) | 0 |
| United Arab Emirates | 2010 | 2019 | 0.5  (0.44 to 0.56) | 0 |
| United Arab Emirates | 2019 | 2021 | 10.31  (9.64 to 10.98) | 0 |
| United Kingdom | 1990 | 2009 | -1.11  (-1.26 to -0.96) | 0 |
| United Kingdom | 2009 | 2019 | 1.23  (0.75 to 1.72) | 0 |
| United Kingdom | 2019 | 2021 | 13.91  (7.93 to 20.22) | 0 |
| United Republic of Tanzania | 1990 | 2002 | -0.05  (-0.07 to -0.03) | 0 |
| United Republic of Tanzania | 2002 | 2011 | -0.42  (-0.45 to -0.38) | 0 |
| United Republic of Tanzania | 2011 | 2019 | 0.18  (0.14 to 0.22) | 0 |
| United Republic of Tanzania | 2019 | 2021 | 6.51  (6.18 to 6.85) | 0 |
| United States Virgin Islands | 1990 | 1994 | -0.78  (-1.25 to -0.3) | 0.003 |
| United States Virgin Islands | 1994 | 2011 | -0.03  (-0.09 to 0.03) | 0.27 |
| United States Virgin Islands | 2011 | 2019 | 0.71  (0.5 to 0.91) | 0 |
| United States Virgin Islands | 2019 | 2021 | 11.44  (9.64 to 13.28) | 0 |
| United States of America | 1990 | 1999 | 3.28  (2.84 to 3.73) | 0 |
| United States of America | 1999 | 2019 | -0.32  (-0.46 to -0.18) | 0 |
| United States of America | 2019 | 2021 | 15.88  (10.53 to 21.49) | 0 |
| Uruguay | 1990 | 1994 | -2.13  (-3.04 to -1.21) | 0 |
| Uruguay | 1994 | 2011 | 0.67  (0.57 to 0.78) | 0 |
| Uruguay | 2011 | 2015 | 2.44  (0.98 to 3.92) | 0.002 |
| Uruguay | 2015 | 2019 | -0.74  (-2.21 to 0.75) | 0.306 |
| Uruguay | 2019 | 2021 | 15.21  (11.3 to 19.25) | 0 |
| Uzbekistan | 1990 | 2019 | -0.28  (-0.31 to -0.25) | 0 |
| Uzbekistan | 2019 | 2021 | 10.13  (8.07 to 12.22) | 0 |
| Vanuatu | 1990 | 2006 | -0.26  (-0.29 to -0.24) | 0 |
| Vanuatu | 2006 | 2011 | -0.73  (-0.93 to -0.52) | 0 |
| Vanuatu | 2011 | 2019 | 0.13  (0.04 to 0.22) | 0.006 |
| Vanuatu | 2019 | 2021 | 5.05  (4.29 to 5.81) | 0 |
| Venezuela (Bolivarian Republic of) | 1990 | 2018 | -0.09  (-0.14 to -0.04) | 0.001 |
| Venezuela (Bolivarian Republic of) | 2018 | 2021 | 4.55  (2.95 to 6.18) | 0 |
| Viet Nam | 1990 | 2003 | 0.07  (-0.05 to 0.19) | 0.259 |
| Viet Nam | 2003 | 2019 | -0.8  (-0.9 to -0.71) | 0 |
| Viet Nam | 2019 | 2021 | 9.63  (6.88 to 12.44) | 0 |
| Yemen | 1990 | 2021 | -0.13  (-0.18 to -0.07) | 0 |
| Zambia | 1990 | 2002 | -0.14  (-0.18 to -0.1) | 0 |
| Zambia | 2002 | 2010 | -0.68  (-0.77 to -0.59) | 0 |
| Zambia | 2010 | 2019 | 0.34  (0.26 to 0.41) | 0 |
| Zambia | 2019 | 2021 | 7.99  (7.12 to 8.86) | 0 |
| Zimbabwe | 1990 | 2000 | -0.5  (-0.61 to -0.39) | 0 |
| Zimbabwe | 2000 | 2006 | 0.13  (-0.18 to 0.44) | 0.386 |
| Zimbabwe | 2006 | 2009 | -0.76  (-2.12 to 0.62) | 0.26 |
| Zimbabwe | 2009 | 2019 | 0.7  (0.57 to 0.83) | 0 |
| Zimbabwe | 2019 | 2021 | 8.02  (6.33 to 9.75) | 0 |

Supplementary Fig. 1 Joinpoint regression analysis of age-standardized prevalence rate of depression among women of childbearing age at the global and four regions (High income, Latin America and Caribbean, North Africa and Middle East, Sub-Saharan Africa) from 1990 to 2021. P-value *P < 0.05.


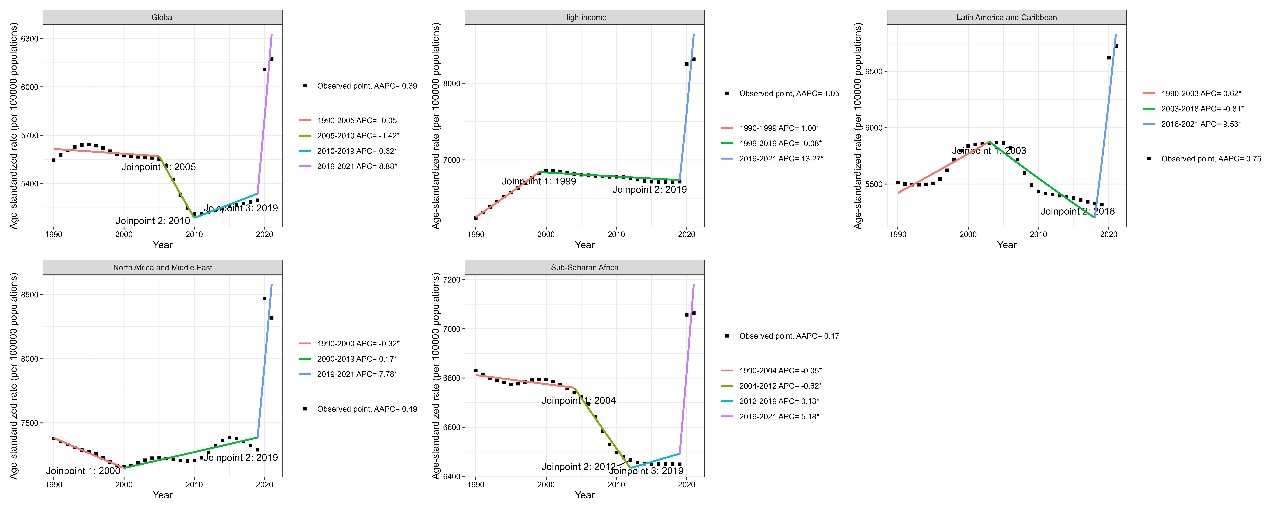


Supplementary Fig. 2 Joinpoint regression analysis of age-standardized DALYs rate of depression among women of childbearing age at the global and four regions (High income, Latin America and Caribbean, North Africa and Middle East, Sub-Saharan Africa) from 1990 to 2021. P-value *P < 0.05.


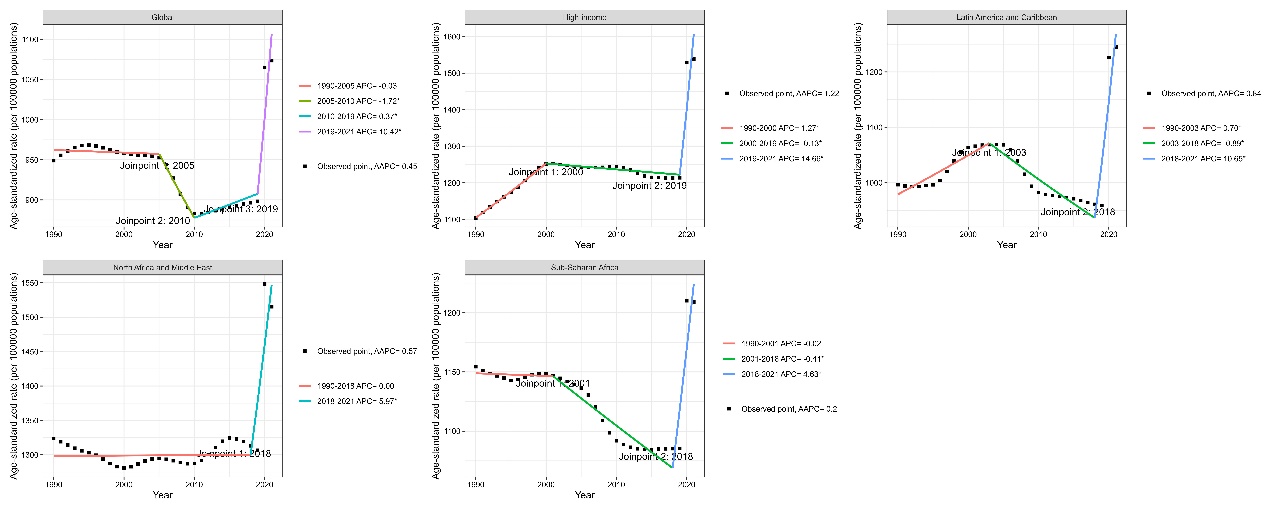

Supplement: Supplementary file 1 [file Data_Sheet_1.docx]
